# Psychometric Evaluation of the Borderline Personality Disorder Checklist

**DOI:** 10.1002/mpr.70029

**Published:** 2025-09-25

**Authors:** Philippa Lynn Mayer, Anna Lisa Westermair, Nele Assmann, Joos Bloo, Natalia Calvo, Chiara De Panfilis, Eva Fassbinder, Marc Ferrer, Gitta Jacob, Juliette Richetin, Anja Schaich, Emanuele Preti, Jan Philipp Klein, Arnoud Arntz

**Affiliations:** ^1^ Department of Psychiatry Psychosomatics and Psychotherapy Lübeck University Lübeck Germany; ^2^ Clinical Ethics Unit University Hospital Basel (USB) University Psychiatric Clinics (UPK) Basel University Children's Hospital Basel (UKBB) Geriatric University Medicine Felix Platter (UAFP) Basel Switzerland; ^3^ Institute of Biomedical Ethics and History of Medicine (IBME) University of Zurich (UZH) Zurich Switzerland; ^4^ Department of Psychiatry and Psychotherapy Lübeck University Lübeck Germany; ^5^ Department of Clinical Psychological Science Maastricht University Maastricht the Netherlands; ^6^ Psychiatry Department Hospital Universitari Vall D'Hebron Centro de Investigación Biomédica en Red de Salud Mental (CIBERSAM) Barcelona Spain; ^7^ Psychiatry Mental Health and Addictions Group Vall D'Hebron Research Institute (VHIR) Universitat Autònoma Barcelona (UAB) Barcelona Spain; ^8^ Unit of Neuroscience Department of Medicine and Surgery Personality Disorders Lab University of Parma Parma Italy; ^9^ Department of Psychiatry and Psychotherapy Christian‐Albrechts‐Universität zu Kiel Kiel Germany; ^10^ GAIA AG Hamburg Hamburg Germany; ^11^ Department of Psychology University of Milano‐Bicocca Milano Italy; ^12^ Department of Clinical Psychology University of Amsterdam Amsterdam the Netherlands

**Keywords:** borderline personality disorder, borderline personality disorder checklist, psychometric validation, reliability, validity

## Abstract

**Background:**

Borderline Personality Disorder (BPD) is a severe and disabling condition. The Borderline Personality Disorder Checklist (BPDCL) was designed to specifically assess the subjective burden of a patient due to BPD symptoms. Various translations have been developed, but an assessment of the psychometric properties of these translations is needed. The aim was to examine the psychometric qualities of the BPDCL across different languages (i.e., Italian, Dutch, German, Spanish, English, and Greek).

**Methods:**

Secondary data was used by reaching out to various researchers, who administered the BPDCL in previous studies. Five studies (*N* = 3199) conducted in Spain, Germany, Italy, the Netherlands, Australia, England, and Greece, were included in the current data set. The BPDCL was administered to BPD patients (*N* = 1131), Axis I disorder patients (*N* = 57), patients with other personality disorders (*N* = 225), and healthy controls (*N* = 1786). Item analyses and analyses assessing the known‐groups and convergent validity were performed to investigate the psychometric properties of the checklist.

**Results:**

Each version of the BPDCL, differing in language, demonstrated high‐reliability coefficients (Cronbach's Alpha ranged from 0.93 to 0.96 and was 0.96 for the entire sample). The correlations between the BPDCL and other instruments, used in the studies, were weak to strong. Correlations greater than 0.55 were observed between the BPDCL and the scales BPDSI, SCL‐90 and the BSI. In addition, the BPDCL seems to differentiate well between diagnostic groups. BPD patients scored the highest, followed by patients with other personality disorders, who in turn scored higher than Axis I disorder patients and healthy controls.

**Conclusions:**

In general, the BPDCL possesses good psychometric properties and seems to be an adequate self‐report instrument to measure the subjective burden of BPD patients.

**Abbreviations**
BPD: Borderline Personality Disorder
BPDCL: Borderline Personality Disorder Checklist
BPDSI: Borderline Personality Disorder Severity Index‐IV
BSI: Brief Symptom Inventory‐53
DSM: Diagnostic and Statistical Manual of Mental Disorders
EuroQoL: European Quality of Life Scale
HC: healthy control
IPO: Inventory of Personality Organization
Md: median
*N*
: frequency
*N*
: sample size
n.a.: not applicable/not assessed
OCPD: Obsessive‐compulsive Personality Disorder
PD: personality disorder
r.: relationships
*r*_tot_
: corrected item‐total correlations
SCID‐II: Structured Clinical Interview for DSM‐IV
SCL‐90: Symptom Checklist‐90 revised
SD: standard deviation
WCCL: Dialectical Behavior Therapy Ways of Coping Checklist
WHOQoL: World Health Organization Quality of Life Questionnaire
WSAS: Work and Social Adjustment Scale
*α*_if item was deleted_
: Cronbach's Alpha if item was deleted

## 1 Background

Borderline Personality Disorder (BPD) is characterized by disturbed self‐image, dysfunctional emotion regulation, and unstable interpersonal relationships (American Psychiatric Association 2013). Prevalence estimates of BPD vary greatly across epidemiological studies. According to a systematic review by Volkert et al. (Volkert et al. 2018), 1.9% of the general adult population suffers from BPD. The prevalence in out‐ and inpatient samples is much higher, ranging from 12% to 22%.

BPD shows high comorbidities with somatic and other mental disorders. The most frequent comorbidities are affective‐, anxiety‐, substance use disorders, as well as other personality disorders. BPD patients show an increased use of mental health services and are likely to be impaired in their social and occupational functioning (Ellison et al. 2018; Tomko et al. 2014; Volkert et al. 2018). BPD is diagnosed using semi‐structured clinical interviews. Particularly in clinical research, instruments are required to evalufic symptoms, but rather evaluate the patient's overall mental health (e.g., Symptom Checklist‐90, SCL‐90; (Derogatis 1992)). There is a sate the severity of the patient's symptoms to assess the effectiveness of treatment. Some measures often used do not focus on BPD‐speciignificant demand for BPD‐specific and time‐saving instruments in order to assess the effectiveness of BPD‐tailored treatments.

### 1.1 Borderline Personality Disorder Checklist

A self‐report measure that assesses the subjective burden of a patient due to BPD symptoms in a time‐saving manner is the Borderline Personality Disorder Checklist (BPDCL, see eAppendix 1 in Supporting Information S1). The original Dutch version, developed by Arntz and Dreessen in 1995 [as cited in (Giesen‐Bloo et al. 2006)], consisted of 47 items, which are supposed to closely reflect the diagnostic criteria of the Borderline Personality Disorder in the Diagnostic and Statistical Manual of Mental Disorders (DSM).

There are other BPD‐related self‐report questionnaires available, as for instance the BSL‐95 (Bohus et al. 2007), BSL‐23 (Wolf et al. 2009) and the BPQ (Poreh et al. 2006). The BPDCL was developed in connection with the *Borderline Personality Disorder Severity Index (BPDSI)*, a semi‐structured clinical interview. These two tools complement each other: the checklist captures subjective distress through self‐reporting, while the interview offers an objective evaluation based on observable symptom patterns (Giesen‐Bloo, Van Dyck, et al. 2006). Thus, the BPDCL offers the advantage of a co‐developed clinical interview. To our knowledge, the BPDCL is currently the only self‐report instrument that systematically assess each DSM‐IV criterion for borderline personality disorder using multiple items per criterion. The combination of the clinician rated interview and the self‐report instrument therefore enables the assessment of changes in the severity of BPD on the criterion level.

The BPDCL allows a detailed evaluation of the presence and severity of the patient's symptoms, while not being redundant or time‐consuming. A shorter questionnaire might miss important clinical aspects and therefore it might lack comprehensiveness.

So far, there have only been a few studies on the psychometric properties of the BPDCL. Bloo et al. (Giesen‐Bloo, Van Dyck, et al. 2006) found the Dutch BPDCL to have good reliability and convergent validity. The BPDCL seemed to discriminate well between BPD patients and other clinical groups. Similar results were found for the Spanish and Italian versions of the BPDCL (Calvo et al. 2018; Richetin et al. 2017). Bloo et al. (Giesen‐Bloo, Van Dyck, et al. 2006) found a nine‐factorial structure, based on the DSM criteria (Diagnostic and Statistical Manual of Mental Disorders), to fit best. The BPDCL has been translated into several languages and has already been used in research studies (Assmann et al. 2024), despite the small number of psychometric studies on the BPDCL.

### 1.2 Current Study

The current study aimed to psychometrically validate the BPDCL. Data on the German, Dutch, Spanish, English, Italian, and Greek versions of the BPDCL were collected.

To our knowledge, this is the first psychometric study that analyzes various translations of the BPDCL independently and combined. Some versions were psychometrically evaluated for the first time (German, English, and Greek). Psychometrically validating the BPDCL is necessary, as self‐report measures help diagnose a mental health disorder and evaluate its severity and subjective impact. The combination of clinician‐rated interviews and self‐report measures is considered superior to either alone (Hopwood et al. 2008).

## 2 Methods and Materials

### 2.1 Procedure

Data was obtained from five different studies, conducted in Spain (Calvo et al. 2018), the Netherlands (Giesen‐Bloo, Van Dyck, et al. 2006), Germany (Assmann et al. 2024), Italy (Richetin et al. 2017) as well as a final study, which was conducted in multiple countries, namely in Australia, Germany, Greece, the Netherlands, and the United Kingdom (Arntz et al. 2022). See eAppendix2 in Supporting Inforamation S1 for a detailed description of each study. The statistical analyses were performed on the total data and on the individual language subsamples to see whether individual translations deviate from the original with respect to their psychometric properties. Results of the total sample are reported in the current study. The individual results of the subsamples are only briefly presented, however, they can be found in the supplementary materials (Supporting Information S2–S7).

### 2.2 Sample

Our dataset included 1131 BPD patients, 1786 healthy controls, 57 Axis I disorder patients, and 225 Axis II disorder patients (non‐BPD). All participants were diagnosed by therapists or experts, by using a DSM‐oriented interview. Exclusion criteria for the healthy controls were any psychological complaints. Healthy controls were non‐help seekers, like for instance university students. Axis I patients did not meet the criteria for BPD or any other personality disorder. All participants were asked to sign an informed consent before the data collection. The studies from which these data have been drawn received ethical approval. The recruitment of participants happened via social networks, advertisements at universities, or participants were directly approached by their therapists or the researchers (description of each study is provided in eAppendix2 Supporting Information S1).

The current study required all respondents to be at least 18 years of age to be included in the analyses (a detailed overview of the excluded cases and plausibility checks is provided in eAppendix3 in Supporting Information S1). In total, 3199 out of 3347 provided cases were used for statistical analyses. The current study was preregistered on aspredicted.org with the following trial number: #103868 (https://aspredicted.org/u3b6h.pdf).

### 2.3 Instruments

Each participant completed the Borderline Personality Disorder Checklist (BPDCL; (Giesen‐Bloo, Van Dyck, et al. 2006)). The Spanish, Italian, German, English, and Greek versions were translated from the validated Dutch BPDCL, then back‐translated into Dutch, and revised if necessary. Additional instruments were administered to the participants, depending on the design of the original study (a detailed description of each instrument can be found in eAppendix4 in Supporting Information S1). Borderline‐specific instruments were the Borderline Personality Disorder Severity Index‐IV (BPDSI; (Arntz et al. 2003)) and the number of BPD criteria assessed through the Structured Clinical Interview for DSM‐IV (SCID‐II; (First 1997; Fydrich et al. 1997)). Additional non‐specific instruments were: Symptom Checklist 90‐Revised (SCL‐90; (Derogatis 1992)), Brief Symptom Inventory‐53 (BSI; (Derogatis 1993)), Work and Social Adjustment Scale (WSAS; (Mundt et al. 2002)), Inventory of Personality Organization (IPO (Kernberg and Clarkin 1995)), World Health Organization Quality of Life Questionnaire (WHOQoL‐BREF; (Group 1998)), the Dialectical Behavior Therapy Ways of Coping Checklist (DBT‐WCCL; (Neacsiu et al. 2010)) and the European Quality of Life Scale (EuroQoL; (Brooks and Group 1996)).

### 2.4 Statistical Analysis

Analyses were performed with the IBM SPSS Statistics program (version 28). Significance levels were set at *p* ≤ 0.05. No missing values were substituted. Due to various missing data and the different study designs, the sample size varied across the analyses.

#### 2.4.1 Internal Consistency and Item Analyses

The Shapiro‐Wilks test and a visual inspection of the data were used to check the assumption of normality of the data (Pallant 2020). Means and standard deviations of each item were reviewed. We looked at the mean‐inter‐item‐correlations, as they give a good indication of the homogeneity of the items. Mean inter‐item correlations between 0.20 and 0.40 were regarded as acceptable (Bühner 2011; Pallant 2020). In addition, we calculated the corrected item‐total‐correlations to see how strongly each item correlates with the total scale. Correlations below 0.30 indicated a weak association between the item and the scale (Bühner 2011; Coaley 2014; Pallant 2020). We calculated Cronbach's alpha if the item was deleted for all items. We did not expect any item to lower the overall reliability coefficient of the total scale. In addition, we estimated reliability coefficients for the total score and each subscale of the BPDCL. Cronbach's Alpha, Guttman's Lambda2 and McDonald's Omega were calculated as measures of internal consistency. A score of 0.70 or higher was regarded as acceptable (Bühner 2011; Trizano‐Hermosilla and Alvarado 2016).

#### 2.4.2 Validity

Convergent validity was analyzed by correlating the BPDCL with other questionnaires. Pearson's correlations (i.e., parametric) and Spearman's Rho (i.e., non‐parametric) were calculated, depending on the assumption of the normality of the data (Gibbons 1993). Correlations of 0.55 or higher were indicative of a strong correlation (i.e., measuring the same construct). Correlations between 0.45 and 0.54 were regarded as acceptable. However, correlations below 0.20 might indicate two separate constructs (Coaley 2014). In addition, we ran the Kruskal‐Wallis test to see whether the BPDCL distinguishes well between the diagnostic groups (i.e., BPD‐patients, other PD patients, Axis I disorder patients, and healthy controls). Bonferroni correction was applied, and median scores were explored (Pallant 2020).

#### 2.4.3 Factorial Analysis

Because the factorial structure of the BPDCL has already been examined in prior research (Giesen‐Bloo et al. 2006), we prioritized evaluating internal consistency and validity in this study. While we did perform an exploratory factor analysis (EFA) during the initial stages, we consider EFA less relevant given the existing findings and therefore report its results only in the supplementary materials (see Supporting Information S1: eAppendix10).

## 3 Results

### 3.1 Total Sample (*N* = 3199)

#### 3.1.1 Descriptives

The total sample consisted of 1131 BPD patients, 225 non‐BPD personality disorder patients, 57 Axis I disorder patients, and 1786 healthy controls. The age ranged from 18 to 69 (mean = 27.32; SD = 8.91). The majority was female (79%), single (58%), currently studying (59%), and came from Western Europe (97%) Demographics and clinical information of each diagnostic group can be seen in Table 1. Significant differences across the diagnostic and language groups with respect to demographics can be found in eAppendix5 in Supporting Information S1.

**TABLE 1:** Demographics and clinical information across diagnostic groups (*N* = 3199).

	BPD (*N* = 1131)	Other PD (*N* = 225)	Axis I (*N* = 57)	Healthy control (1786)
	*n* (%)	*n* (%)	*n* (%)	*n* (%)
Gender				
Female	959 (84.87)	134 (60.09)	33 (62.26)	1407 (78.78)
Male	169 (14.96)	89 (39.91)	20 (37.74)	379 (21.22)
Other	2 (0.18)	0 (0.00)	0 (0.00)	0 (0.00)
Marital status				
Single	668 (59.33)	134 (60.91)	19 (35.85)	34 (39.53)
Married or lasting relationship	359 (31.88)	61 (27.73)	28 (52.83)	45 (52.33)
Separated	78 (6.93)	23 (10.45)	4 (7.55)	5 (5.81)
Widowed	2 (0.18)	1 (0.45)	0 (0.00)	2 (2.33)
Other	19 (1.69)	1 (0.45)	2 (3.77)	0 (0.00)
Nationality				
Western europe	138 (100.00)	55 (100.00)	57 (100)	1720 (96.68)
Eastern europe	0 (0.00)	0 (0.00)	0 (0.00)	30 (1.69)
Latin America	0 (0.00)	0 (0.00)	0 (0.00)	19 (1.07)
Africa	0 (0.00)	0 (0.00)	0 (0.00)	6 (0.34)
Asia	0 (0.00)	0 (0.00)	0 (0.00)	4 (0.22)
Employment				
Homemaker	67 (6,04)	10 (4.67)	2 (3.77)	13 (0.73)
Student	117 (10.55)	24 (11.21)	5 (9.43)	1728 (96.81)
Sickness benefits	335 (30.21)	24 (11.21)	28 (52.83)	6 (0.34)
Working	266 (23.99)	88 (41.12)	14 (26.42)	30 (1.68)
Unemployed	223 (20.11)	63 (29.44)	0 (0.00)	3 (0.17)
Retired	9 (0.81)	3 (1.40)	4 (7.55)	4 (0.22)
No legal income	2 (0.18)	0 (0.00)	0 (0.00)	0 (0.00)
Other	90 (8.12)	2 (0.93)	0 (0.00)	1 (0.06)
Axis I disorder				
Affective	681 (67.36)	74 (37.95)	10 (40.00)	0 (0.00)
Anxiety	615 (71.51)	79 (40.72)	17 (68.00)	0 (0.00)
Substance use	453 (55.24)	65 (33.33)	2 (8.00)	0 (0.00)
Eating	275 (38.25)	19 (9.79)	0 (0.00)	0 (0.00)
Other	334 (42.12)	22 (11.34)	3 (12.00)	0 (0.00)
Axis II disorder				
Paranoid PD	248 (22.98)	26 (12.94)	0 (0.00)	0 (0.00)
Schizoid PD	15 (1.39)	6 (2.99)	0 (0.00)	0 (0.00)
Schizotypal PD	19 (1.76)	6 (2.99)	0 (0.00)	0 (0.00)
Antisocial PD	67 (6.64)	30 (14.93)	0 (0.00)	0 (0.00)
Narcissistic PD	49 (4.50)	17 (8.46)	0 (0.00)	0 (0.00)
Borderline PD	1144 (100.00)	0 (0.00)	0 (0.00)	0 (0.00)
Histrionic PD	59 (5.46)	19 (9.45)	0 (0.00)	0 (0.00)
Dependent PD	133 (12.34)	22 (10.95)	0 (0.00)	0 (0.00)
Avoidant PD	349 (32.26)	43 (21.39)	0 (0.00)	0 (0.00)
OCPD	232 (21.54)	67 (33.33)	0 (0.00)	0 (0.00)
Unspecified PD	188 (20.39)	83 (41.29)	0 (0.00)	0 (0.00)
Age	Mean (SD)	Mean (SD)	Mean (SD)	Mean (SD)
	31.91 (9.23)	35.03 (10.74)	34.13 (10.28)	23.24 (5.65)

*Note:* Due to the differing study designs, not all variables were applied in each study, resulting in many missing values. Only valid percentages are reported.

Abbreviations: *n* = frequency; *N* = sample size; OCPD = Obsessive‐compulsive personality disorder; PD = personality disorde; SD = standard deviation.

#### 3.1.2 Item Analyses and Reliability Measures

The assumption of normality of the data was not met (*p* < 0.001, see eAppendix6 in Supporting Information S1). The results of the item analyses are presented in Table 2. Item means ranged from 1.13 (item 25) to 3.34 (item 2) with an item means mean of 2.07. The total scale mean for the total sample was 97.05 (SD = 33.55), with individual scores ranging from 47 to 235. The corrected item total correlations lay between 0.16 (Item 8) and 0.74 (Item 11 and 30). All corrected item‐total correlations were above the predefined value of 0.30, except for items 8, 17 and 35.

**TABLE 2:** Item analyses and reliability measures of the BPDCL for the total dataset.

		Mean	SD	*r*_tot_	*α*_if item was deleted_
1	Money spending	2.24	1.24	0.43	0.96
2	Quick changes of mood	3.35	1.24	0.66	0.96
3	Tantrums	2.49	1.24	0.55	0.96
4	Depersonalization	2.18	1.29	0.55	0.96
5	Hitting others	1.32	0.78	0.41	0.96
6	Self‐mutilation	1.46	1.00	0.54	0.96
7	Attracted to women or men?	1.42	0.94	0.37	0.96
8	Gambling	1.16	0.57	0.16	0.96
9	Urge to kill yourself	1.60	1.10	0.63	0.96
10	Uncertainty who you are	2.57	1.37	0.70	0.96
11	Feeling empty inside	3.04	1.37	0.74	0.96
12	Drinking	1.69	1.07	0.31	0.96
13	Fear that others leave	2.83	1.40	0.63	0.96
14	Being different in various situations	2.17	1.29	0.69	0.96
15	Uncertainty about what should life look like	3.24	1.29	0.65	0.96
16	Convicted that others treat me unfairly	2.06	1.21	0.68	0.96
17	Drug use	1.36	0.87	0.27	0.96
18	Strong changes of feelings for others	2.44	1.29	0.70	0.96
19	Distrusting others	2.78	1.23	0.62	0.96
20	Not dare to recognize bad sides of yourself	2.06	1.13	0.46	0.96
21	If others get to know me, they will leave	2.35	1.40	0.69	0.96
22	Reckless driving	1.60	1.00	0.34	0.96
23	Derealization	1.57	0.99	0.45	0.96
24	Acts in life threatening ways	1.48	0.93	0.55	0.96
25	Feelings of despair	2.62	1.45	0.78	0.96
26	Trying to kill yourself	1.31	0.87	0.49	0.96
27	Losing senses, because you are convicted that others will leave you	1.74	1.26	0.64	0.96
28	Threat others that you will hurt/kill yourself	1.30	0.86	0.52	0.96
29	Binge eating	2.08	1.32	0.50	0.96
30	Finding yourself a bad and unacceptable person	2.22	1.37	0.74	0.96
31	Being convicted that others have it in for your	1.45	0.94	0.56	0.96
32	Not knowing what friends or loved ones you want to have	2.23	1.30	0.59	0.96
33	Feelings that are unacceptable to you	2.23	1.37	0.73	0.96
34	Not knowing what is actually important to you	2.54	1.32	0.68	0.96
35	Shoplifting	1.13	0.53	0.23	0.96
36	Sudden anxieties, depressions or irritability	2.98	1.41	0.75	0.96
37	Becoming so angry that you lose control and break things	1.60	1.09	0.54	0.96
38	Not being able to remember important things	2.13	1.23	0.57	0.96
39	Being very suspicious	2.47	1.29	0.65	0.96
40	Feeling terribly disappointed in someone you first admired or loved	2.73	1.44	0.60	0.96
41	Acting on an impulsive sexual contact you later regretted	1.43	0.95	0.35	0.96
42	Suddenly losing trust in other people	2.31	1.28	0.68	0.96
43	The conviction that you're not able to deal with life on your own	2.49	1.40	0.70	0.96
44	Hating yourself, everybody and the world	2.19	1.39	0.76	0.96
45	Frantically trying to prevent others from leaving you	1.84	1.25	0.61	0.96
46	Uncertainty about what your true standards and values are	2.24	1.29	0.66	0.96
47	Not knowing anymore what you have done or where you are	1.41	0.91	0.55	0.96

*Note: N* = 3199 for the mean and the SD of the BPDCL. *N* = 3182 for the corrected item‐total correlation and Cronbach's Alpha if item was deleted, as cases were excluded listwise for those calculations.

Abbreviations: *α*_if item was deleted_ = Cronbach's Alpha if ITEM was deleted; *r*_tot_ = corrected item‐total correlations; SD = standard deviation.

The mean inter‐item correlation of the total sample was 0.34, which was within the predefined range of 0.20–0.40. Cronbach's Alpha if item was deleted did not change the overall Cronbach's Alpha, which was 0.96 (*N* = 3182). The Guttman Lambda_2_ coefficient (0.97) and the McDonald's Omega coefficient (0.96) were equally high (see eAppendix7 in Supporting Information S1 for reliability coefficients of each subscale of the BPDCL).

#### 3.1.3 Convergent Validity

The Spearman's Rho correlations across the BPDCL total score and other instruments can be seen in Table 3, ranging from weak to strong correlations. For a detailed overview of subscale correlations across the instruments see eAppendix8 in Supporting Information S1.

**TABLE 3:** Spearman's Rho correlations of the BPDCL total score and other instruments.

	BPDSI	#BPD criteria	EuroQoL utility	WSAS	IPO^a^	WCCL^a^	SCL‐90	BSI	WHOQOL^a^
BPDCL	0.71	0.54	−0.25	0.26	0.54–0.58	−0.03–0.53	0.90	0.77	0.14–0.48

*Note:* Correlations across BPDCL total score and the total scores of other instruments or the range of correlations across the BPDCL total score and subscores of other instruments are depicted.

Abbreviations: #BPD criteria = number of SCID‐II criteria; BPDSI = borderline personality disorder severity index; BSI = brief symptom inventory‐53; EuroQoL utility = european quality of life questionnaire utility score; IPO = inventory of personality organization; SCL‐90 = symptom checklist‐90‐revised; WCCL = dialectical‐behavior therapy ways of coping checklist; WHOQOL = world health organization quality of life questionnaire; WSAS = work and social adjustment scale.

^a^ These instrument have multiple subscales, the range of correlations is given.

#### 3.1.4 Known‐Groups Validity

The Kruskal‐Wallis test revealed a statistically significant difference in the BPDCL total score across the four diagnostic groups (χ^2^(3, *n* = 3182) = 1105.13, *p* < 0.001). All groups differed significantly from one another, except Axis I disorder patients and healthy controls (*p* = 0.392; detailed overview in eAppendix9 in Supporting Information S1). When looking at the medians (Md) of the BPDCL total score, the BPD sample scored higher than the other groups (Md = 121, *n* = 1123). Other PD patients (Md = 94, *n* = 216) scored significantly lower than BPD patients and significantly higher than Axis I disorder patients (Md = 71, *n* = 57) and healthy controls (Md = 76, *n* = 1786).

### 3.2 Results of the Individual BPDCL Versions (Differing in Language)

We examined each version of the BPDCL on its own. Table 4 broadly displays the results of each BPDCL version (a detailed overview of each version is given in Supporting Information S1).

**TABLE 4:** Overview of the BPDCL versions and their psychometric properties.

Criterion	German	Italian	Dutch	Spanish	English	Greek
Sample	BPD	BPD, HC & other PD	BPD, other PD, axis I & HC	BPD & other PD	BPD	BPD
Item analysis						
Inter item correlation	0.23	0.29	0.32	0.25	0.27	0.28
*r*_tot_	0.01–0.63	0.28–0.68	0.03–0.81	0.09–0.68	−0.14–0.77	0.10–0.83
Cronbach's *α*	0.93	0.95	0.96	0.94	0.95	0.95
*α* range of subscales	0.64–0.83	0.67–0.85	0.65–0.90	0.63–0.81	0.66–0.84	0.63–87
Scales with *α* < 0.70	Interpersonal *r*.	Anger	Impulsivity	Interpersonal *r*. Impulsivity, anger	Impulsivity	Parasuicidal *b*.
	Impulsivity					
	Parasuicidal *b*.					
Guttman Lamda2	0.94	0.95	0.97	0.94	0.95	0.95
McDonald'sOmega	n.a.	0.95	0.97	0.94	0.96	0.94
Convergent validity						
BPDSI	0.56	n.a.	0.75	n.a.	0.66	0.78
BSI	0.74	n.a.	0.83	n.a.	0.83	0.59
SCL‐90	n.a.	0.90	0.90	n.a.	n.a.	n.a.
EuroQoL utility	−0.37	n.a	−0.43	n.a.	−0.24	n.a.
WSAS	0.33	n.a	0.25	n.a.	0.11	0.35
Known‐groups validity						
BPD > others	n.a.	*p* = 0.009	*p* < 0.001	*p* < 0.001	n.a	n.a.

*Note:* The adjusted significance is depicted with regards to known‐groups validity. Only a few mental health instruments are listed here. For more details see supplementary materials.

Abbreviations: *α* = cronbach's alpha; *b*. = behavior; BPD = borderline personality disorder; BPDSI = borderline personality disorder severity index total score; BSI = brief symptom inventory total score; EuroQoL utility = european quality of life index (the utility score for the pertinent country was used); HC = healthy control; n.a. = not applicable/not assessed; PD = personality disorder, *r*. = relationships; *r*_tot_ = corrected iter item correlation; SCL‐90 = symptom checklist total score; WSAS = work and social adjustment scale.

#### 3.2.1 Item Analyses and Reliability Meausres

Mean inter‐item correlations were within the predefined range of 0.20–0.40 for all versions. The corrected item‐total correlations were above 0.30 for most items, with a few exceptions in each dataset. Deleting any of those items (*r*_tot_ < 0.30) did not change the overall Cronbach's Alpha of the total scale. The total Cronbach's Alpha varied from 0.93 (German version) to 0.96 (Dutch version). Most BPDCL subscales reached reliability coefficients above 0.70. The subscales *Impulsivity, Interpersonal relationships* and *Parasuicidal behaviors* had reliability coefficients slightly below 0.70 across multiple versions.

#### 3.2.2 Validity

Convergent validity with the BPDSI, BSI, and the SCL‐90 was good (*ρ* > 0.55) for each version of the BPDCL. The EuroQoL utility score and the WSAS total score correlated weakly with individual BPDCL versions. Correlations across corresponding subscales were strong in each version of the BPDCL (e.g., *ρ* > 0.55 across BPDCL *Anger* and SCL‐90 *Hostility*). BPD‐specific instruments did not correlate stronger with the BPDCL compared to non‐specific instruments across all versions. The German BPDCL seemed to have the lowest correlations with the other instruments compared to the other BPDCL versions. One possible explanation is the restriction of range, as the German sample consisted of BPD patients only.

The known‐groups validity was good for the Italian, Spanish, and Dutch versions of the BPDCL. BPD patients scored significantly higher on the BPDCL total score compared to the other groups. The known‐groups validity for the German, English, and Greek versions could not be calculated, as there was only data on BPD patients.

## 4 Discussion

The current study aimed to psychometrically evaluate the BPDCL, a measure that assesses the subjective burden of BPD symptoms on BPD patients. Secondary data from 3199 participants and six different versions of the BPDCL (i.e., German, Dutch, Spanish, Italian, English, and Greek) were obtained.

The current study reports the reliability indices of multiple versions of the BPDCL, ranging from 0.93 to 0.96. The item analyses revealed adequate corrected item‐total correlations for the majority of the items, supporting the internal consistency of the scale. Interestingly, items relating to gambling (item 8), drug use (item 17) and shoplifting (item 35) showed relatively low corrected item‐total correlations (< 0.30). These behaviors may not represent core features of the borderline personality disorder, but rather reflect comorbid or externalizing tendencies such as impulsivity or antisocial behavior. These tendencies are als prevalent in other psychiatric disorders (Axis I and Axis II) and thus may not be unique to borderline personality traits (Sunderland et al. 2015).

Regarding the convergent validity, correlations with other mental health instruments were strong for the total sample. The BPDCL correlated strongly with certain subscales of the BPDSI, BSI‐53, SCL‐90, IPO, WCCL, and the WHOQoL. When looking at the individual BPDCL versions, correlations across the BPDCL and any other instrument were moderate to strong. Compared to the other versions of the BPDCL, the German version seems to demonstrate less strong, however still acceptable, convergent validity with other mental health instruments. One possible explanation might be the restriction of range, as the German sample consisted of BPD‐patients only. Interestingly, the BPDCL did not correlate stronger with BPD‐specific questionnaires (BPDSI & SCID‐II) compared to other mental health instruments. A possible explanation for the discrepancies may be the mode of evaluation. The BPDSI and the SCID‐II are clinician‐rated instruments, whereas the other instruments were self‐report measures. Cross‐informant measures do not always demonstrate good convergent validity, even though the measures were designed to assess construct‐specific symptoms (Bradley et al. 2007). In the total sample and all examined translations (Dutch, Italian, and Spanish), the BPDCL seems to discriminate well between patients with BPD and the other diagnostic groups. BPD patients scored significantly higher on the BPDCL than the other patient groups or healthy controls.

All in all, the current results concerning reliability, convergent, and known‐groups validity were in line with previous studies on the BPDCL (Calvo et al. 2018; Giesen‐Bloo et al. 2006; Richetin et al. 2017). In addition, we assessed the psychometric properties of BPDCL versions that had not been examined before (e.g., English, German, and Greek translations). Since we used secondary data, we could examine a relatively large sample compared to previous studies. The current study consisted of data on BPD patients and clinical and healthy controls, which allowed us to look at the known‐groups validity of the BPDCL. Due to the different study designs, the sample sizes across the clinical groups and statistical analyses varied greatly. Originally, the studies were not conducted to assess the psychometric properties of the BPDCL, which resulted in different study designs (e.g., different patient settings), which in turn might have reduced the comparability of the studies. We performed a duplicate analysis based on demographics and clinical information in order to avoid statistical bias and to prevent misclassification (e.g. one person appears in two diagnostic groups). As the data were pooled from different studies, it is possible ‐ though very unlikely ‐ that some participants were included more than once. We consider the likelihood of substantial participant overlap influencing our results as very low. When interpreting our findings, it should be considered that a small number of duplicates cannot be entirely excluded. Despite some limitations, the current study provides meaningful results with respect to the BPDCL.

We would suggest further research on the psychometric properties of the BPDCL. The known‐groups validity needs to be explored for the German, English, and Greek BPDCL versions. To our knowledge, so far only data on BPD patients are available. It is also advised to examine further the change sensitivity of the individual translations of the BPDCL. Other studies have already found the BPDCL sensitive to change (see (Arntz et al. 2022; Fassbinder et al. 2016; Giesen‐Bloo et al. 2006)).

Future research should explore the factorial structure of each BPDCL version independently to see whether they differ from one another concerning their factorial organization. Finally, one could further examine the factorial invariance of the BPDCL across languages.

## 5 Conclusion

Our findings are in line with previous research on the psychometric properties of the BPDCL. Thus, the BPDCL seems to be a suitable psychological instrument for clinical and research purposes.

## Author Contributions

**Philippa Lynn Mayer:** conceptualization (equal), formal analysis (lead), investigation (lead), methodology (lead), project administration, software (lead), visualization, writing – original draft preparation, writing – review and editing (lead). **Anna Lisa Westermair:** conceptualization (equal), investigation, formal analysis, methodology, software, data curation, writing – review and editing. **Nele Assmann:** resources, investigation, writing – review and editing. **Joos Bloo:** resources, investigation, writing – review and editing. **Natalia Calvo:** resources, investigation, writing – review and editing. **Chiara De Panfilis:** resources, investigation, writing – review and editing. **Eva Fassbinder:** resources, investigation, writing – review and editing. **Marc Ferrer:** resources, investigation, writing – review and editing. **Gitta Jacob:** resources, investigation, writing – review and editing. **Juliette Richetin:** resources, investigation, writing – review and editing. **Anja Schaich:** resources, investigation, writing – review and editing. **Emanuele Preti:** resources, investigation, writing – review and editing. **Jan Philipp Klein:** supervision, resources, investigation, formal analysis, methodology, writing – review and editing, project administration, conceptualization (equal). **Arnoud Arntz:** supervision (lead), resources, investigation, formal analysis, methodology, writing – review and editing, project administration (lead).

## Ethics Statement

Briefly, data included in this psychometric analysis was from individual studies where ethics approval had been granted and participants provided informed consent. The ethical review boards of the following institutions have approved the individual studies: Lübeck University, Maastricht University, Person Università di Parma, Università degli Studi di Milano‐Bicocca, Vall d’Hebron University Hospital, Peel Mental Health Service, Rockingham Mental Health Service, University of Freiburg, IVAH GmbH Hamburg (Institut for training CBT), Eginition Hospital Athen, Athens University, De Viersprong Amsterdam/Duivendrecht, Mondriaan Mental Health Institute Heerlen, Mental Health Institute GGZ Oostbrabant Helmond, Symfora GGZ Centraal Mental Health Institute Hilversum, Community Mental Health Center Maastricht, Vincent van Gogh Mental Health Institute Venlo, Vincent van Gogh Mental Health Institute Venray, Bradford District Care NHS Foundation Trust and Maudsley NHS Foundation Trust London.

## Consent

The authors have nothing to report.

## Conflicts of Interest

The authors declare no conflicts of interest.

## Supporting information

Supporting Information S1

## Data Availability

The data that support the findings of this study are available on request from the authors of the individual studies that this analysis is based on. Supplementary materials, containing the Borderline Personality Disorder Checklist, a description of the indidivual studies and further results of each BPDCL version, can be openly accessed (see Supporting Information S1–S7).
