# Supplementary material for: Psychometric Evaluation of the Borderline Personality Disorder Checklist
Source: Int J Methods Psychiatr Res. 2025 Sep 25;34(3):e70029. doi: 10.1002/mpr.70029 (PMC12461754; doi:10.1002/mpr.70029)
Supplement: Supplementary file 1 — Supporting Information S1 [file MPR-34-e70029-s001.zip › SM1-Total sample.docx]

**Supplementary material for the total dataset (SM1)**

**eAppendix 1.** English version of the Borderline Personality Disorder Checklist

**eAppendix 2.** Overview of the individual studies

**eAppendix 3.** Exclusion criteria of current study

**eAppendix 4**. Overview of psychological instruments

**eTable 1.** Means and standard deviations of each instrument

**eAppendix 5.** Group differences in demographics

**eTable 2.** Across language groups

**eTable 3.** Across diagnostic groups

**eAppendix 6.** Test of normality of the data

**eTable 4.** Results of Shapiro-Wilks test

**eAppendix 7.** Reliability coefficients of BPDCL subscales

**eTable 5.** Total sample

**eTable 6.** BPD sample

**eAppendix 8.** Convergent validity

**eTable 7.** BPDCL and BPDSI

**eTable 8.** BPDCL and EuroQoL, IPO, WSAS and DBT-WCCL

**eTable 9.** BPDCL and SCL-90 and BSI-53

**eTable 10.** BPDCL and WHO

**eAppendix 9.** Known-groups validity

**eTable 11.** Results of Kruskal-Wallis test

**eAppendix 10.** Exploratory factor analysis

**eFigure 1.** Scree plot

**eTable 12.** Total variance explained by Eigenvalues

**eTable 13.** Correlation matrix of seven-factor solution

**eTable 14.** Factor loadings of seven-factor solution

**eTable 15.** Factor loadings of nine-factor solution

**eTable 16.** Correlation matrix of nine-factor solution

This supplementary material has been provided by the authors to give readers additional information about their work.

**eAppendix 1.** English version of the Borderline Personality Disorder Checklist (BPDCL)

## **Borderline Personality Disorder Checklist (Giesen-Bloo, Arntz, & Schouten, 2006)**

Arnoud Arntz  &  Laura Dreessen

University of Maastricht

Department of Medical Clinical and Experimental Psychology

Post box 616

6200 MD  Maastricht

The Netherlands

English translation: Josephine Giesen-Bloo, Maastricht, August 2001

During last month, to what extent were you troubled by:

| 1 | Impulsive spending of too much money that you cannot afford to spend | 1 | 2 | 3 | 4 | 5 |
| --- | --- | --- | --- | --- | --- | --- |
| 2 | Quick changes of mood | 1 | 2 | 3 | 4 | 5 |
| 3 | Tantrums | 1 | 2 | 3 | 4 | 5 |
| 4 | Not feeling oneself anymore, like an outside observer of  yourself, or experiencing yourself as in a movie or dream  (not  because of drugs) | 1 | 2 | 3 | 4 | 5 |
| 5 | Hitting others or throwing things at others | 1 | 2 | 3 | 4 | 5 |
| 6 | Injuring yourself on purpose (cutting, pricking, hitting, burning) | 1 | 2 | 3 | 4 | 5 |
| 7 | Not knowing whether you actually feel attracted to men or women | 1 | 2 | 3 | 4 | 5 |
| 8 | Gambling | 1 | 2 | 3 | 4 | 5 |
| 9 | The urge to kill yourself | 1 | 2 | 3 | 4 | 5 |
| 10 | Uncertainty about who you really are | 1 | 2 | 3 | 4 | 5 |
| 11 | Feeling bored or empty inside | 1 | 2 | 3 | 4 | 5 |
| 12 | Drinking too much | 1 | 2 | 3 | 4 | 5 |
| 13 | Fear that others will leave you | 1 | 2 | 3 | 4 | 5 |
| 14 | Being so different in various situations or with other people  that you don't know who you are anymore | 1 | 2 | 3 | 4 | 5 |
| 15 | Uncertainty about what your life should look like | 1 | 2 | 3 | 4 | 5 |
| 16 | Being convinced that others are treating you unfairly | 1 | 2 | 3 | 4 | 5 |
| 17 | Drug use | 1 | 2 | 3 | 4 | 5 |
| 18 | Strong changes in feelings for other people | 1 | 2 | 3 | 4 | 5 |
| 19 | Distrusting other people | 1 | 2 | 3 | 4 | 5 |
| 20 | Not daring to recognize the bad sides of yourself | 1 | 2 | 3 | 4 | 5 |
| 21 | The idea that if others really get to know you, they will reject you | 1 | 2 | 3 | 4 | 5 |
| 22 | Reckless driving (car, motor, bike) |  |  |  |  |  |
| 23 | Observing or experiencing the world around you totally differently so that it seems very odd or unreal to you  (e.g., others look unfamiliar or like 'robots'; not because of drugs) | 1 | 2 | 3 | 4 | 5 |
| 24 | The tendency to act in life threatening ways (e.g., in traffic) | 1 | 2 | 3 | 4 | 5 |
| 25 | Feelings of despair | 1 | 2 | 3 | 4 | 5 |
| 26 | Trying to kill yourself | 1 | 2 | 3 | 4 | 5 |
| 27 | Losing your senses because you are convinced/think   that somebody who's important to you, will leave you | 1 | 2 | 3 | 4 | 5 |
| 28 | Threatening other people that you will injure or kill yourself | 1 | 2 | 3 | 4 | 5 |
| 29 | Binge eating | 1 | 2 | 3 | 4 | 5 |
| 30 | Finding yourself a bad and unacceptable person | 1 | 2 | 3 | 4 | 5 |
| 31 | Being convinced that others have it in for you  (that you're being persecuted) | 1 | 2 | 3 | 4 | 5 |
| 32 | Not knowing what friends or loved ones you want to have | 1 | 2 | 3 | 4 | 5 |
| 33 | Feelings that are unacceptable to you | 1 | 2 | 3 | 4 | 5 |
| 34 | Not knowing what is actually important to you | 1 | 2 | 3 | 4 | 5 |
| 35 | Shoplifting | 1 | 2 | 3 | 4 | 5 |
| 36 | Sudden anxieties, depressions or irritability | 1 | 2 | 3 | 4 | 5 |
| 37 | Becoming so angry that you lose control and break things | 1 | 2 | 3 | 4 | 5 |
| 38 | Not being able to remember important things  (not because of drugs) | 1 | 2 | 3 | 4 | 5 |
| 39 | Being very suspicious | 1 | 2 | 3 | 4 | 5 |
| 40 | Feeling terribly disappointed in someone you first admired  or loved | 1 | 2 | 3 | 4 | 5 |
| 41 | Acting on an impulsive sexual contact you later regretted | 1 | 2 | 3 | 4 | 5 |
| 42 | Suddenly losing trust in other people | 1 | 2 | 3 | 4 | 5 |
| 43 | The conviction that you're not able to deal with life on your own | 1 | 2 | 3 | 4 | 5 |
| 44 | Hating yourself, everybody and the world | 1 | 2 | 3 | 4 | 5 |
| 45 | Frantically trying to prevent others from leaving you | 1 | 2 | 3 | 4 | 5 |
| 46 | Uncertainty about what your true standards and values are | 1 | 2 | 3 | 4 | 5 |
| 47 | Not knowing anymore what you have done or where you are  (not because of drugs) | 1 | 2 | 3 | 4 | 5 |

**eAppendix 2.** Overview of the individual studies

***Study 1***

The first study was conducted in Spain by Calvo and colleagues (Calvo et al., 2018). Participants were recruited at the Psychiatry Department of the General Hospital in Barcelona. This sample consists of BPD patients and clinical controls (any non-BPD personality disorder). Being aged between 18 and 60 years was required. Exclusion criteria were a below average intelligence, learning disability, and a current diagnosis of schizophrenia, bipolar disorder, substance dependence disorder or any organic condition that could better explain the symptoms. In order to be included in one of the patient groups one had to meet the criteria for at least one personality disorder. The Structured Clinical Interview for the DSM-IV Axis I Disorders and Axis II Personality Disorders (SCID-I;(First, 1997); SCID-II; (First & Gibbon, 2004)) were used for clinical assessment. The Spanish version of the BPDCL was used. For further details see (Calvo et al., 2018). Four hundred fifteen participants of the study were included for the current analyses.

***Study 2***

The second study was conducted in Italy at various study sites (e.g. public mental health center and at universities) by Richetin and colleagues (Richetin, Preti, Costantini, & De Panfilis, 2017). This sample consists of BPD-patients, healthy controls and clinical controls (any non-BPD personality disorder). Participants had to be aged between 18 and 75 years. In order to be included in one of the patient groups one had to meet the criteria for at least one personality disorder. Healthy controls were recruited at the universities Milano-Bicocca and Parma. Exclusion criteria were cognitive impairment and a current manic or psychotic episode. A non-structured DSM-oriented clinical interview was conducted in order to assess mental health disorders of the participants. The Italian version of the BPDCL was used. For further details see (Richetin et al., 2017). We included 1795 participants of the study for the current analyses.

***Study 3***

The third study was conducted in the outpatient clinic of the Department of Psychiatry and Psychotherapy at Lübeck university (Germany) by Assmann and colleagues (Assmann et al., 2024). This sample consists of BPD-patients only. Inclusion criteria were age between 18 and 65 years, primary diagnosis of Borderline Personality Disorder and a BPDSI score higher than 20 (indicative of clear and severe BPD). Exclusion criteria were intellectual deficits (intelligence quotient below 85) and a lifetime major psychotic disorder or an acute severe substance dependence disorder.  The SCID- II interview was conducted for the assessment of mental disorders. The German version of the BPDCL was used. For further details see (Assmann et al., 2024). We included 158 participants of the study for the current analyses.

***Study 4***

The fourth study was conducted in 12 in- and outpatient centers in the Netherlands by Bloo and colleagues (Giesen-Bloo et al., 2006). Participants had to be aged between 18 and 60 years. This sample consists of BPD-patients, healthy controls and clinical controls (any Cluster C personality disorder or Axis I disorders only). Overall exclusion criteria were mental retardation, current psychotic- or bipolar disorders, or any psychiatric disorder secondary to a medical condition. The study consisted of three groups: BPD patients, clinical controls (Cluster C patients) and healthy controls. Exclusion criteria for healthy controls were any psychological complaints or disorders. Clinical controls had to meet criteria for at least one Cluster C personality disorder. Exclusion criteria for the clinical control group were diagnosis of Cluster A or B personality disorder. The SCID-I and II were conducted for the assessment of mental disorders. The Dutch version of the BPDCL was used. For further details see (Giesen-Bloo et al., 2006). We included 337 participants of the study for the current analyses.

***Study 5***

The group schema therapy study was conducted at 15 study sites in Australia, United Kingdom, Greece, the Netherlands and Germany (Arntz et al., 2022). Inclusion criteria were age between 18 and 65 years, primary diagnosis of Borderline Personality Disorder and a Borderline Personality Disorder Severity Index score higher than 20. Exclusion criteria were intellectual deficits (intelligence quotient below 80), inability to read, speak or write the language at the assessment center, a lifetime psychotic disorder (except brief psychotic disorder), ADHD (Attention Deficit Hyperactivity Disorder; unless successfully treated), bipolar disorder type 1, dissociative identity disorder, full or subthreshold narcissistic or Antisocial Personality Disorder, substance dependence disorder,  serious and/or unstable medical illness and having received schema therapy for more than 3 months in the past 3 years. The SCID-I and II, as well as the WHO ADHD screener were used for the assessment of mental disorders. The BPDCL was provided in various languages depending on the study site (i.e. German, Dutch, English and Greek). For further details see (Arntz et al., 2022). We included 494 participants of the study for the current analyses.

**eAppendix 3.** Exclusion criteria of current study

## **Exclusion criteria for the current study**

Data of 3347 cases was provided by the researchers. Twelve Dutch cases did not have any information about the sample. It was not clear to which clinical group (BPD, other PD, Axis I disorder or healthy controls) they belonged to and those cases did not fill out the BPDCL (i.e. only missing values). Cases without BPDCL data were excluded from the current data set. Two Dutch cases were classified as BPD patients, but did only meet four BPD criteria. We decided to delete those two cases, as we could not say with certainty which variable was misleading in those cases. Some cases were initially lacking specific clinical information (n=109), except for the information on the clinical group (BPD, other PD, Axis I disorder or healthy patient). The data on the clinical information was not made available by the researchers at the time the analyses were performed. We decided to keep those cases, as we did not control for comorbidities. Twenty-four cases (1 Italian, 1 English & 22 Spanish) were younger than 18 years, which violates our inclusion criterion. Seventy-eight cases of the Spanish data set were excluded, as they did not meet the diagnosis of any personality disorder, even though this was the inclusion criterion of Calvo et al. (Calvo et al., 2018). Two German cases were excluded, as they did not fulfill their inclusion criterion (i.e. diagnosis of BPD). Fourteen additional cases did not fill out the BPDCL and thus were excluded (13 German & 1 Italian). Finally, we performed a duplicate analysis as we used data from multiple studies and wanted to make sure that there are no duplicates in the total sample. Sixteen duplicates were found and excluded from the analyses. To sum up, 148 cases were excluded for the analyses, which is about 4.42% of all provided cases.

**eAppendix 4**. Overview of psychological instruments

***Borderline Personality Checklist (BPDCL)***

All studies administered the Borderline Personality Checklist (BPDCL). This self-report measure consists of 47 items. The patients are asked to rate their subjective burden during the past four weeks on a 5-point Likert scale, ranging from “Not at all” to “Extremely” (Giesen-Bloo et al., 2006). The items are based on the DSM-IV diagnostic criteria of BPD: efforts to avoid abandonment (Q13, Q18, Q21, Q27, Q28, Q43 and Q45) , unstable relationships (Q32, Q40 and Q42), identity disturbance (Q7, Q10, Q14, Q15, Q20, Q30, Q34 and Q46), impulsivity (Q1, Q8, Q12, Q17, Q22, Q24, Q29, Q35 and Q41), parasuicidal behavior (Q6, Q9 and Q26), affective instability ( Q2, Q25, Q33 and Q36), difficulty controlling anger (Q3, Q5, Q37 and Q44), chronic feelings of emptiness (Q11) and lastly dissoziation and paranoid ideation (Q4, Q16, Q19, Q23, Q31, Q38, Q39 and  Q47). Nine subscores can be calculated by summing up all items belonging to that scale. The total score is the sum of all items (Giesen-Bloo et al., 2006; Richetin et al., 2017).

***Borderline Personality Disorder Severity Index-IV (BPDSI)***

The BPDSI-IV is a semi-structured interview used to assess the severity and frequency of the nine DSM-IV BPD-criteria. The participant is asked to rate the symptom frequency on a 11-point scale, 0 indicating “Never” and 11 indicating “Daily”. Only the items assessing identity disturbance are rated on a 5-point Likert scale, ranging from 0 (“Absent”) to 4 (“Dominant”). Criteria scores and a total score can be calculated (Arntz et al., 2003). The BPDSI consists of 70 items in total. It has adequate psychometric qualities in samples with in- and outpatients (Arntz et al., 2003). The BPDSI can also be used as treatment outcome measure, as it is sensitive to change over time (Arntz et al., 2003).

***Symptom Checklist 90-Revised (SCL)***

The SCL-90-R is a self-report questionnaire, consisting of 90 items, which assess psychological symptoms in psychiatric and medical patients (Derogatis, 1992). Each item can be rated on a 5-point Likert scale, running from 0 “Not at all” to 4 “Very much”. The Dutch version and the Italian version of the SCL-90-R consist of different subscales. Thus, we are not able to compare those subscales across the samples. However, we will look at the total sum score of the 90 items (GS), which is indicative of the overall psychiatric impairment. In addition, we will look at the correlation between the BPDCL and the Global Severity Index (GSI), Positive Symptom Total (PST) and the Positive Symptom Distress Index (PSDI) of the Italian sample. The SCL-90-R has proven high reliability (e.g. Cronbach’s α ranging from 0.96 to 0.98 for the GSI) and satisfactory factorial validity across multiple language versions (Derogatis, 1993; Prinz et al., 2013; Smits, Timmerman, Barelds, & Meijer, 2014).

***Brief Symptom Inventory (BSI)***

The BSI is the short version of the SCL-90-R and consists of 53 items. The BSI resulted from a factor analysis of the SCL-90-R, and thus the items are based on the same dimensions. Both instruments contain the nine subscales and three indices. Participants are asked to rate their symptoms on a 5-point Likert scale. It has good psychometric qualities and can be seen as an adequate alternative to the SCL-90-R. We calculated the mean scores of the following scales: Somatization, Obsession, Insecurity, Depression, Anxiety, Hostility, Phobic Anxiety, Paranoid ideation and Psychoticism. In addition, the total sum score, also called GS index, was calculated by adding all item scores together (Derogatis, 1993; Derogatis & Melisaratos, 1983).

***European Quality of Life Scale (EuroQoL)***

The EuroQoL is a self-report measure, which assesses five health-related dimensions: mobility, self-care, usual activities, pain/discomfort, and anxiety/depression. Participants have to rate the items on a three-level scale, indicating the severity or presence of a symptom (e.g. mobility: “I have no problems in walking about”, “I have some problems in walking about” and “I am confined to bed”). In addition to the five dimensions, they are asked to rate their current health state on a scale from 0 to 100 (VAS scale; (Brooks, 1996)). The EuroQoL has good test-retest reliability in several somatic-patient samples and seems to be a useful instrument for assessing the quality of life of borderline personality disorder patients (Dorman, Slattery, Farrell, Dennis, & Sandercock, 1998; Tsang et al., 2019; Van Asselt, Dirksen, Arntz, Giesen-Bloo, & Severens, 2009). We converted the raw data into the EQ-5D-3L index score. The syntax was provided by the EuroQoL website. The Dutch, English and German versions of the EuroQoL required a language specific SPSS syntax (https://euroqol.org/information-and-support/resources/value-sets/, [cited 2023 Feb 4]).

***Work and Social Adjustment Scale (WSAS)***

The WSAS is a self-report measure, assessing the impairment of daily functioning in terms of work, home management, social leisure, private leisure and personal or family relationships. Participants have to rate each of the five items on a scale, running from 0 (“No impairment”) to 8 (“Very severe impairment”). A total score of 40 can be reached, indicating a very high form of impairment. The psychometric qualities were analyzed in various clinical samples (Mataix-Cols et al., 2005; Mundt, Marks, Shear, & Greist, 2002). For instance, the WSAS has a good to excellent internal consistency in a sample of phobic patients (Cronbach’s Alpha ranged from .71 to .90, depending on the type of phobia; (Mataix-Cols et al., 2005).

***Inventory of Personality Organization (IPO)***

The IPO consists of 83 items, which are rated by the participant on a 5-point Likert scale (“Never true” to “Always true”). It comprises three main scales, namely Identity diffusion (ID), Primitive Defensen (PD) and Reality Testing (RT), as well as two newly developed scales (Aggression and Moral Values). Good psychometric qualities of the inventory were proven in clinical and non-clinical samples (Berghuis, Kamphuis, Boedijn, & Verheul, 2009; Kernberg & Clarkin, 1995). The current study only included the data of the three main scales.

***World Health Organization Quality of Life Questionnaire (WHOQoL-BREF)***

The WHOQOL-BREF is a short version of the WHOQoL-100, which assesses the participants well-being on six domains. In contrast, the WHOQoL-BREF assesses only four domains: physical, psychological, social relationships and environmental aspects (Group, 1998). The version used in the current data set consists of 35 items, as questions regarding the participant’s self-esteem and positive and negative emotions were added (Group, 1998; Wetzelaer et al., 2014).

***Dialectical-Behavioral Ways of Coping Checklist (DBT-WCCL)***

The DBT-WCCL is a 59 item self-report measure. Participants are asked to rate their skill use on a four-point Likert scale. Three subscores can be calculated: Skill use, dysfunctional coping and blaming others (Neacsiu, Rizvi, Vitaliano, Lynch, & Linehan, 2010). The WCCL proved to have excellent internal consistency (α=.94) and high convergent validity with other questionnaires that measure skill use (Stein, Hearon, Beard, Hsu, & Björgvinsson, 2016).

**eTable 1.**

*Means and standard deviations of each instrument (N=3199)*

| Instrument | n | Min | Max | Mean | SD |
| --- | --- | --- | --- | --- | --- |
| BPDSI- Abandonment | 898 | 0 | 9 | 2.83 | 2.01 |
| BPDSI- Interpersonal relationships | 898 | 0 | 8 | 2.35 | 1.59 |
| BPDSI- Identity distrubance | 898 | 0 | 10 | 4.35 | 2.48 |
| BPDSI- Impulsivity | 898 | 0 | 6 | 1.41 | 1.11 |
| BPDSI- Parasuicidal b. | 898 | 0 | 7 | .97 | 1.04 |
| BPDSI- Affective inst. | 898 | 0 | 10 | 6.28 | 2.89 |
| BPDSI- Emptiness | 898 | 0 | 10 | 5.48 | 2.78 |
| BPDSI- Anger | 898 | 0 | 10 | 2.74 | 1.98 |
| BPDSI- Dissociation | 898 | 0 | 10 | 2.22 | 1.84 |
| BPDSI- Total score | 917 | 0 | 70 | 28.67 | 13.02 |
| EQ-5D-3L | 620 | 0 | 1 | .60 | 0.22 |
| IPO - Identity Diffusion | 174 | 1 | 78 | 25.80 | 27.24 |
| IPO - Primitive Psychological Defenses | 174 | 1 | 66 | 19.70 | 20.37 |
| IPO-Alternative Reality Testing | 174 | 1 | 61 | 13.11 | 14.08 |
| WSAS Total score | 649 | 2 | 40 | 23.41 | 7.70 |
| SCL-GS | 342 | 90 | 372 | 191.13 | 72.60 |
| SCL-Somatization Mean | 94 | 1 | 5 | 2.24 | 0.93 |
| SCL-Obsessive Mean | 93 | 1 | 5 | 2.56 | 0.96 |
| SCL-Insecurity Mean | 94 | 1 | 5 | 2.28 | 1.01 |
| SCL-Depression Mean | 94 | 1 | 5 | 2.83 | 1.03 |
| SCL- Anxiety Mean | 94 | 1 | 5 | 2.35 | 0.95 |
| SCL-Hostility Mean | 94 | 1 | 5 | 1.78 | 0.74 |
| SCL Phobic anxiety Mean | 94 | 1 | 4 | 1.79 | 0.87 |
| SCL- Paranoid Mean | 94 | 1 | 5 | 2.27 | 0.91 |
| SCL-Psychoticism Mean | 94 | 1 | 5 | 2.10 | 0.75 |
| BSI- Somatization | 652 | 0 | 4 | 1.29 | 0.87 |
| BSI- Obsession | 652 | 0 | 4 | 2.18 | 0.86 |
| BSI-Insecurity | 652 | 0 | 4 | 2.23 | 0.97 |
| BSI-Depression | 652 | 0 | 4 | 2.34 | 0.94 |
| BSI-Anxiety | 652 | 0 | 4 | 1.82 | 0.94 |
| BSI-Hostility | 652 | 0 | 4 | 1.61 | 0.97 |
| BSI-Phobic Anxiety | 652 | 0 | 4 | 1.45 | 1.04 |
| BSI-Paranoid | 652 | 0 | 4 | 1.80 | 0.91 |
| BSI-Psychoticism | 652 | 0 | 4 | 1.77 | 0.82 |
| BSI-GS | 623 | 15 | 209 | 96.65 | 37.13 |
| WCCL-Skill Use | 145 | 0 | 3 | 1.51 | 0.48 |
| WCCL-Dysfunctional coping | 145 | 0 | 3 | 2.33 | 0.46 |
| WCCL-Blaming others | 145 | 0 | 3 | 1.61 | 0.74 |
| WHO-Self-esteem | 652 | 1 | 4 | 2.11 | 0.68 |
| WHO-Negative feelings | 652 | 1 | 5 | 3.44 | 1.03 |
| WHO-Positive feelings | 652 | 1 | 4 | 2.25 | 0.65 |
| WHO-Physical | 652 | 4 | 88 | 39.70 | 13.04 |
| WHO-Psychological | 652 | 0 | 90 | 35.31 | 13.35 |
| WHO - Social | 652 | 0 | 100 | 39.30 | 21.62 |
| WHO- Environment | 652 | 6 | 97 | 53.24 | 15.28 |

*Note.* n = Frequency (i.e. number of participants who filled out the given questionnaire), Min= Minimum score, Max= Maximum score, SD= standard deviation, BPDSI= Borderline Personality Disorder Severity Index, EQ-5D-3L= EuroQoL index, IPO= Inventory of Personality Organization, WSAS= Work and Social Adjustment Scale, SCL= Symptom Checklist-90 revised, GS= Sum score, BSI= Brief Symptom Inventory-53, WCCL=Dialectical Behavioral Therapy Ways of Coping Checklist, WHO= World Health Organization Quality of Life questionnaire.

**eAppendix 5.** Group differences in demographics

**eTable 2.**

*Differences in demographics across language groups (N=3199)*

|  | X^2^ | df | p |
| --- | --- | --- | --- |
| Gender | 39.68 | 10 | <.001 |
| Marital status | 146.57 | 20 | <.001 |
| Setting | 785.09 | 5 | <.001 |
| Nationality | 12.10 | 4 | .017 |
| Ethnicity | 35.73 | 20 | .017 |
| Employment | 3564.75 | 35 | <.001 |
|  | F | df1/df2 | p |
| Age | 183.59 | 5/236.86 | <.001 |

*Note.* X^2^ = Chi Square statistic, df= degree of freedom, p= p-value,

F= Welch ANOVA F-statistic.

**eTable 3.**

*Differences in demographics across diagnostic groups (N=3199)*

|  | X^2^ | df | p |
| --- | --- | --- | --- |
| Gender | 86.22 | 6 | <.001 |
| Marital status | 48.10 | 12 | <.001 |
| Setting | 113.08 | 2 | <.001 |
| Nationality | 8.54 | 12 | .742 |
| Ethnicity | 10.56 | 15 | .78 |
| Employment | 2645.33 | 21 | <.001 |
|  | F | df1/df2 | p |
| Age | 342.84 | 3/209.70 | <.001 |

*Note.* X^2^ = Chi Square statistic, df= degree of freedom, p= p-value,

F= Welch ANOVA F-statistic.

**eAppendix 6.** Test of normality of the data

**eTable 4.**

*Shapiro Wilk test of normality*

| Items |  | Statistic | df | Sig. |
| --- | --- | --- | --- | --- |
| item 1 | Money spending | .85 | 3182 | <.001 |
| item 2 | Quick changes of mood | .90 | 3182 | <.001 |
| item 3 | Tantrums | .89 | 3182 | <.001 |
| item 4 | Depersonalization | .82 | 3182 | <.001 |
| item 5 | Hitting others | .47 | 3182 | <.001 |
| item 6 | Self-mutilation | .53 | 3182 | <.001 |
| item 7 | Attracted to women or men? | .52 | 3182 | <.001 |
| item 8 | Gambling | .30 | 3182 | <.001 |
| item 9 | Urge to kill yourself | .60 | 3182 | <.001 |
| item 10 | Uncertainty who you are | .87 | 3182 | <.001 |
| item 11 | Feeling empty inside | .89 | 3182 | <.001 |
| item 12 | Drinking | .69 | 3182 | <.001 |
| item 13 | Fear that others leave | .89 | 3182 | <.001 |
| item 14 | Being different in various situations | .82 | 3182 | <.001 |
| item 15 | Uncertainty about what should life look like | .90 | 3182 | <.001 |
| item 16 | Convicted that others treat me unfairly | .81 | 3182 | <.001 |
| item 17 | Drug use | .47 | 3182 | <.001 |
| item 18 | Strong changes of feelings for others | .87 | 3182 | <.001 |
| item 19 | Distrusting others | .90 | 3182 | <.001 |
| item 20 | Not dare to recognize bad sides of yourself | .83 | 3182 | <.001 |
| item 21 | If others get to know me, they will leave | .83 | 3182 | <.001 |
| item 22 | Reckless driving | .65 | 3182 | <.001 |
| item 23 | Derealization | .63 | 3182 | <.001 |
| item 24 | Acts in life threatening ways | .58 | 3182 | <.001 |
| item 25 | Feelings of despair | .86 | 3182 | <.001 |
| item 26 | Trying to kill yourself | .41 | 3182 | <.001 |
| item 27 | Losing senses, because you are convicted that others will leave you | .63 | 3182 | <.001 |
| item 28 | Threat others that you will hurt/kill yourself | .40 | 3182 | <.001 |
| item 29 | Binge eating | .78 | 3182 | <.001 |
| item 30 | Finding yourself a bad and unacceptable person | .80 | 3182 | <.001 |
| item 31 | Being convicted that others have it in for your | .54 | 3182 | <.001 |
| item 32 | Not knowing what friends or loved ones you want to have | .83 | 3182 | <.001 |
| item 33 | Feelings that are unacceptable to you | .81 | 3182 | <.001 |
| item 34 | Not knowing what is actually important to you | .88 | 3182 | <.001 |
| item 35 | Shoplifting | .27 | 3182 | <.001 |
| item 36 | Sudden anxieties, depressions or irritability | .89 | 3182 | <.001 |
| item 37 | Becoming so angry that you lose control and break things | .61 | 3182 | <.001 |
| item 38 | Not being able to remember important things | .82 | 3182 | <.001 |
| item 39 | Being very suspicious | .88 | 3182 | <.001 |
| item 40 | Feeling terribly disappointed in someone you first admired or loved | .87 | 3182 | <.001 |
| item 41 | Acting on an impulsive sexual contact you later regretted | .51 | 3182 | <.001 |
| item 42 | Suddenly losing trust in other people | .85 | 3182 | <.001 |
| item 43 | The conviction that you're not able to deal with life on your own | .86 | 3182 | <.001 |
| item 44 | Hating yourself. everybody and the world | .79 | 3182 | <.001 |
| item 45 | Frantically trying to prevent others from leaving you | .70 | 3182 | <.001 |
| item 46 | Uncertainty about what your true standards and values are | .83 | 3182 | <.001 |
| item 47 | Not knowing anymore what you have done or where you are | .52 | 3182 | <.001 |

*Note*. df= degress of freedom. Cases were excluded listwise. Only 3182 cases were included in the analysis of the normal distribution.

**eAppendix 7.** Reliability coefficients of BPDCL subscales

**eTable 5.**

*Reliability coefficients of each BPDCL subscale for the total sample (N=3199)*

|  | Cronbach’s Alpha | Guttman Lambda_2_ | McDonald’s Omega |
| --- | --- | --- | --- |
| Fear of abandonment | .85 | .86 | .86 |
| Interpersonal relationships | .74 | .75 | .76 |
| Identity disturbance | .87 | .88 | .88 |
| Impulsivity | .71 | .72 | .71 |
| Parasuicidal behavior | .82 | .82 | .83 |
| Affective instability | .87 | .87 | .87 |
| Emptiness | - | - | - |
| Anger | .75 | .76 | .76 |
| Paranoid and dissociative behavior | .84 | .84 | .84 |
| Total scale | .96 | .97 | .96 |

*Note.* Reliability coefficients for the emptiness subscale could not be calculated, as it consists of only one item.

**eTable 6.**

*Reliability coefficients of each BPDCL subscale for the BPD sample (N=1131)*

|  | Cronbach’s Alpha | Guttman Lambda_2_ | McDonald’s Omega |
| --- | --- | --- | --- |
| Fear of abandonment | .80 | .81 | .80 |
| Interpersonal relationships | .69 | .69 | .70 |
| Identity disturbance | .81 | .81 | .81 |
| Impulsivity | .63 | .64 | .63 |
| Parasuicidal behavior | .76 | .76 | .77 |
| Affective instability | .78 | .78 | .78 |
| Emptiness | - | - | - |
| Anger | .74 | .75 | .75 |
| Paranoid and dissociative behavior | .81 | .81 | .79 |
| Total scale | .94 | .94 | .94 |

*Note.* Reliability coefficients for the emptiness subscale could not be calculated, as it consists of only one item. McDonald’s Omega should be cautiously looked at, since the lack of normality of the data.

**eAppendix 8.** Convergent validity

**eTable 7.**

*Spearman’s Rho correlations of the BPDCL and BPDSI*

|  | BPDSI Abandon | BPDSI  Relation | BPDSI  Identity | BPDSI  Impulsive | BPDSI  Parasuicid. | BPDSI  Affective | BPDSI  Emptiness | BPDSI Anger | BPDSI  Dissociative | BPDSI  Total | # BPD criteria |
| --- | --- | --- | --- | --- | --- | --- | --- | --- | --- | --- | --- |
| BPDCL subscales |  |  |  |  |  |  |  |  |  |  |  |
| Abandonment | **.63**** | .49** | .49** | .44** | .50** | .49** | .45** | .40** | .47** | **.64**** | .50 |
| Interpersonal relationships | .45** | **.49**** | .48** | .42** | .44** | .43** | .41** | .38** | .47** | **.57**** | .45 |
| Identity disturbance | .44** | .47** | **.61**** | .48** | .52** | .46** | .46** | .37** | .51** | **.63**** | .50 |
| Impulsivity | .35** | .39** | .40** | **.74**** | .39** | .38** | .40** | .35** | .39** | **.53**** | .41 |
| Parasuicidal behavior | .32** | .33** | .36** | .38** | **.71**** | .35** | .32** | .28** | .40** | **.47**** | .39 |
| Affective instability | .47** | .48** | .50** | .47** | .58** | **.54**** | .48** | .43** | .51** | **.64**** | .46 |
| Emptiness | .40** | .38** | .44** | .41** | .45** | .50** | **.52**** | .34** | .41** | **.56**** | .43 |
| Anger | .44** | .50** | .46** | .41** | .47** | .51** | .39** | **.64**** | .44** | **.62**** | .48 |
| Paranoid ideation | .40** | .49** | .48** | .43** | .49** | .49** | .44** | .43** | **.65**** | **.63**** | .47 |
| Total score | **.52**** | **.53**** | **.56**** | **.55**** | **.57**** | **.54**** | **.49**** | **.47**** | **.57**** | **.71**** | **.54** |

*Note.* ** Correlation is significant at the 0.01 level (two-tailed). * Correlation is significant at the 0.05 level (two-tailed). Correlations without a star are not significant. Important findings are highlighted (bold and underlined). BPDCL= Borderline Personality Disorder Checklist, BPDSI= Borderline Personality Disorder Severity Index, #BPD criteria= Number of Borderline Personality Disorder criteria, according to SCID-II.

**eTable 8.**

*Spearman's Rho correlations of the BPDCL and other psychological instruments for the total sample*

|  | EQ-5D-3L | IPO  Identity | IPO Defenses | IPO  Reality testing | WSAS  Total score | WCCL  Skill use | WCCL Dysfunctional | WCCL Blaming |
| --- | --- | --- | --- | --- | --- | --- | --- | --- |
| BPDCL subscales |  |  |  |  |  |  |  |  |
| Abandonment | -.17** | .56** | **.55**** | .48** | .21** | -.02 | .41** | .42** |
| Interpersonal relationships | -.24** | .39** | .40** | .34** | .18** | .06 | .48** | .40** |
| Identity disturbance | -.17** | **.60**** | .53** | **.53**** | .24** | -.07 | .48** | .32** |
| Impulsivity | -.05 | .19* | .17* | .20** | .15** | -.11 | .24** | .18** |
| Parasuicidal behavior | -.14** | .26** | .26** | .27** | .17** | -.10 | .32** | .11 |
| Affective instability | -.32** | .52** | .50** | .46** | .30** | -.07 | **.51**** | .31** |
| Emptiness | -.30** | .41** | .37** | .39** | .24** | -.17* | .36** | .22** |
| Anger | -.19** | .46** | .44** | .43** | .19** | -.03 | .41** | **.51**** |
| Paranoid ideation | -.28** | .46** | .48** | **.53**** | .22** | .07 | .46** | .33** |
| Total score | **-.25**** | **.58**** | **.56**** | **.54**** | **.26**** | **-.03** | **.53**** | **.41**** |

*Note.* ** Correlation is significant at the 0.01 level (two-tailed). * Correlation is significant at the 0.05 level (two-tailed). Correlations without a star are not significant. EQ-5D-3L= EuroQoL index, IPO=Inventory of Personality Organization (subscales: Identity diffusion, Primitive psychological defenses, and Alternative reality testing), WSAS= Work and Social Adjustment scale, WCCL= Ways of Coping Checklist (Subscales: Skill use, Dysfunctional coping and Blaming others). The convergent validity with the WCCL was based on the English and Dutch WCCL data only. The German WCCL was rated on a different response scale.

**eTable 9.**

*Spearman’s Rho correlations of the BPDCL and the SCL-90-R and then BSI-53*

|  | SCL Depression | | SCL  Anxiety | | SCL  Hostility | | SCL Paranoid | | SCL  Total Score | | BSI Depression | BSI  Anxiety | | BSI  Hostility | | BSI  Paranoid | | BSI  Total Score | |
| --- | --- | --- | --- | --- | --- | --- | --- | --- | --- | --- | --- | --- | --- | --- | --- | --- | --- | --- | --- |
| BPDCL subscales |  |  | |  | |  | |  | |  | | |  | |  | |  | |  |
| Abandonment | .72** | .70** | | .63** | | .66** | | **.83**** | | .53** | | | .47** | | .53** | | .54** | | **.61**** |
| Interpersonal relationships | .65** | .60** | | .56** | | .59** | | **.75**** | | .48** | | | .45** | | .50** | | .61** | | **.62**** |
| Identity disturbance | .70** | .65** | | .64** | | .63** | | **.81**** | | .60** | | | .43** | | .47** | | .52** | | **.63**** |
| Impulsivity | .36** | .42** | | .37** | | .28** | | **.62**** | | .33** | | | .28** | | .30** | | .30** | | **.36**** |
| Parasuicidal behavior | .59** | .49** | | .43** | | .42** | | **.66**** | | .58** | | | .36** | | .34** | | .32** | | **.52**** |
| Affective instability | **.76**** | **.75**** | | .61** | | .63** | | **.88**** | | **.69**** | | | **.57**** | | .59** | | .49** | | **.73**** |
| Emptiness | .67** | .58** | | .48** | | .42** | | **.80**** | | .63** | | | .39** | | .38** | | .37** | | **.57**** |
| Anger | .64** | .62** | | **.71**** | | .63** | | **.78**** | | .41** | | | .38** | | **.75**** | | .47** | | **.54**** |
| Paranoid ideation | .63** | .63** | | .59** | | **.73**** | | **.83**** | | .50** | | | .52** | | .53** | | **.68**** | | **.72**** |
| Total score | **.80**** | **.76**** | | **.70**** | | **.78**** | | **.90**** | | **.66**** | | | **.56**** | | **.65**** | | **.65**** | | **.77**** |

*Note.* ** Correlation is significant at the 0.01 level (two-tailed). * Correlation is significant at the 0.05 level (two-tailed). Important findings are highlighted (bold).  Correlations without a star are not significant. BPDCL= Borderline Personality Disorder Checklist, SCL= Symptom Checklist-90 revised, BSI= Brief Symptom Inventory-53. Due to space constraints, not all SCL and BSI subscales are presented in this table.

**eTable 10.**

*Spearman's Rho correlations of the BPDCL and WHO subscales*

|  | WHO  Self-esteem | WHO  Negative | WHO  Positive | WHO  Physical | WHO Psychological | WHO  Social | WHO Environment |
| --- | --- | --- | --- | --- | --- | --- | --- |
| BPDCL subscales |  |  |  |  |  |  |  |
| Abandonment | -.40** | .15** | -.26** | -.23** | -.33** | -.25** | -.21** |
| Interpersonal relationships | -.32** | .12** | -.27** | -.16** | -.31** | -.35** | -.20** |
| Identity disturbance | **-.52**** | .08* | -.43** | -.28** | **-.48**** | -.33** | -.21** |
| Impulsivity | -.22** | .06 | -.19** | -.16** | -.27** | -.21** | -.17** |
| Parasuicidal behavior | -.36** | .10* | -.36** | -.22** | -.40** | -.20** | -.17** |
| Affective instability | **-.50**** | .21** | **-.45**** | -.31** | **-.48**** | -.25** | -.18** |
| Emptiness | -.38** | .20** | -.43** | -.35** | -.38** | -.28** | -.21** |
| Anger | -.32** | .07 | -.28** | -.16** | -.29** | -.19** | -.18** |
| Paranoid ideation | -.34** | .08* | -.33** | -.18** | -.37** | -.24** | -.28** |
| Total score | **-.48**** | **.14**** | **-.41**** | **-.28**** | **-.47**** | **-.33**** | **-.26**** |

*Note.* ** Correlation is significant at the 0.01 level (two-tailed). * Correlation is significant at the 0.05 level (two-tailed). Important findings are highlighted (bold).  Correlations without a star are not significant. BPDCL= Borderline Personality Disorder Checklist, WHO= World health Organization Quality of Life questionnaire (Subscales: self-esteem, negative feelings, Positive feelings, Physical, Psychological, Social and Environmental quality of life.

**eAppendix 9.** Known-groups validity

**eTable 11.**

*Known-groups validity of the BPDCL subscales (N=3199)*

| Kruskal-Wallis post hoc tests | | | |
| --- | --- | --- | --- |
| Subscale | Sample I | Sample J | Adjusted significance |
| Abandonment | BPD | Other PD | .000 |
|  | BPD | Axis I | .000 |
|  | BPD | Healthy control | .000 |
|  | Other PD | Axis I | .000 |
|  | Other PD | Healthy control | .000 |
|  | Axis I | Other PD | .000 |
|  | Axis I | Healthy control | 1.000 |
| Interpersonal relationships | BPD | Other PD | .000 |
|  | BPD | Axis I | .000 |
|  | BPD | Healthy control | .000 |
|  | Other PD | Axis I | .000 |
|  | Other PD | Healthy control | .028 |
|  | Axis I | Other PD | .001 |
|  | Axis I | Healthy control | .032 |
| Identity disturbance | BPD | Other PD | .000 |
|  | BPD | Axis I | .000 |
|  | BPD | Healthy control | .000 |
|  | Other PD | Axis I | .000 |
|  | Other PD | Healthy control | .000 |
|  | Axis I | Other PD | .000 |
|  | Axis I | Healthy control | .190 |
| Impulsivity | BPD | Other PD | .000 |
|  | BPD | Axis I | .000 |
|  | BPD | Healthy control | .000 |
|  | Other PD | Axis I | .000 |
|  | Other PD | Healthy control | .001 |
|  | Axis I | Other PD | .000 |
|  | Axis I | Healthy control | .001 |
| Parasuicidal behavior | BPD | Other PD | .000 |
|  | BPD | Axis I | .000 |
|  | BPD | Healthy control | .000 |
|  | Other PD | Axis I | .000 |
|  | Other PD | Healthy control | .000 |
|  | Axis I | Other PD | .000 |
|  | Axis I | Healthy control | 1.000 |
| Affective instability | BPD | Other PD | .000 |
|  | BPD | Axis I | .000 |
|  | BPD | Healthy control | .000 |
|  | Other PD | Axis I | .000 |
|  | Other PD | Healthy control | .000 |
|  | Axis I | Other PD | .000 |
|  | Axis I | Healthy control | 1.000 |
| Emptiness | BPD | Other PD | .000 |
|  | BPD | Axis I | .000 |
|  | BPD | Healthy control | .000 |
|  | Other PD | Axis I | .000 |
|  | Other PD | Healthy control | .000 |
|  | Axis I | Other PD | .000 |
|  | Axis I | Healthy control | 1.000 |
| Anger | BPD | Other PD | .000 |
|  | BPD | Axis I | .000 |
|  | BPD | Healthy control | .000 |
|  | Other PD | Axis I | .000 |
|  | Other PD | Healthy control | .000 |
|  | Axis I | Other PD | .000 |
|  | Axis I | Healthy control | .001 |
| Paranoid and dissociation symptoms | BPD | Other PD | .000 |
|  | BPD | Axis I | .000 |
|  | BPD | Healthy control | .000 |
|  | Other PD | Axis I | .001 |
|  | Other PD | Healthy control | .000 |
|  | Axis I | Other PD | .001 |
|  | Axis I | Healthy control | .479 |
| Total score | BPD | Other PD | .000 |
|  | BPD | Axis I | .000 |
|  | BPD | Healthy control | .000 |
|  | Other PD | Axis I | .000 |
|  | Other PD | Healthy control | .000 |
|  | Axis I | Other PD | .000 |
|  | Axis I | Healthy control | 0.392 |

*Note.* BPD= Borderline Personality Disorder, other PD= non-BPD Personality disorder. The adjusted significance after Bonferroni correction is displayed.

**eAppendix 10.** Exploratory factor analysis

The 47 items were subjected to a principal axis factoring analysis (N=3199). Bartlett's test of sphericity was significant (χ²(1081)=80800.00, *p*<.001). The Kaiser-Meyer-Olkin measure of sampling adequacy was above the predefined value of .60, namely .97. Both measures approved the suitability of the data for exploratory factor analysis. The scree plot inspection would suggest a one-factor solution (see eFigure 1). According to the Eigenvalue criterion (>1), seven factors were identified (see eTable 12). The seven factors would explain 56.88% of the total variance, with the first factor explaining 36.94% of the variance. Performing Horn’s parallel analysis, the Eigenvalues of the seven factors all exceed the corresponding criterion values for a randomly generated data matrix of the same size (47 items, N=3199). Those factors may be used for further exploration. The seven factors correlated strongly with each other. Correlation coefficients ranged from .06 to .78. (see eTable 13).

Factor loadings (>.30) after Promax rotation are presented in eTable 14. Based on the items the factors were labeled as followed: *Identity disturbance* (e.g., Uncertainty about who you really are’ ), *Paranoid fear of abandonment* (e.g., ‘Suddenly losing trust in other people’), (*Para)suicidal behavior* (e.g., ‘Try to kill yourself’), *Impulsivity* (e.g., ‘Impulsive spending of too much money that you cannot afford to spend’), *Difficulty controlling anger* (e.g., ‘Becoming so angry that you lose control and break things’), *Dissociative symptoms* (e.g., ‘Not feeling oneself anymore, like an outside observer of yourself, or experiencing yourself as in a movie or dream’) and *Affective instability* (e.g., ‘Sudden anxieties, depressions or irritability’). An additional principal axis factoring was conducted with the factor number set to nine, based on the DSM criteria (eTable15).

Bloo et al. (Giesen-Bloo et al., 2006) conducted confirmatory factor analyses based on several theory-driven models and found a nine-factor solution based on DSM criteria, to fit the data best. In the initial stage of our research, we decided to look at the current data exploratively, as it is not biased by previous theories. A principal axis factoring analysis included the total data set (N=3199). Interestingly, we identified seven factors that greatly resembled the ones proposed by Bloo et al. (Giesen-Bloo et al., 2006). To correctly compare the current results with those found by Bloo et al. (Giesen-Bloo et al., 2006), one could perform additional confirmatory factor analyses. Also, further investigation of the independent translations is needed to see whether the factorial structure varies across languages.

**eFigure 1.**

*Scree plot*


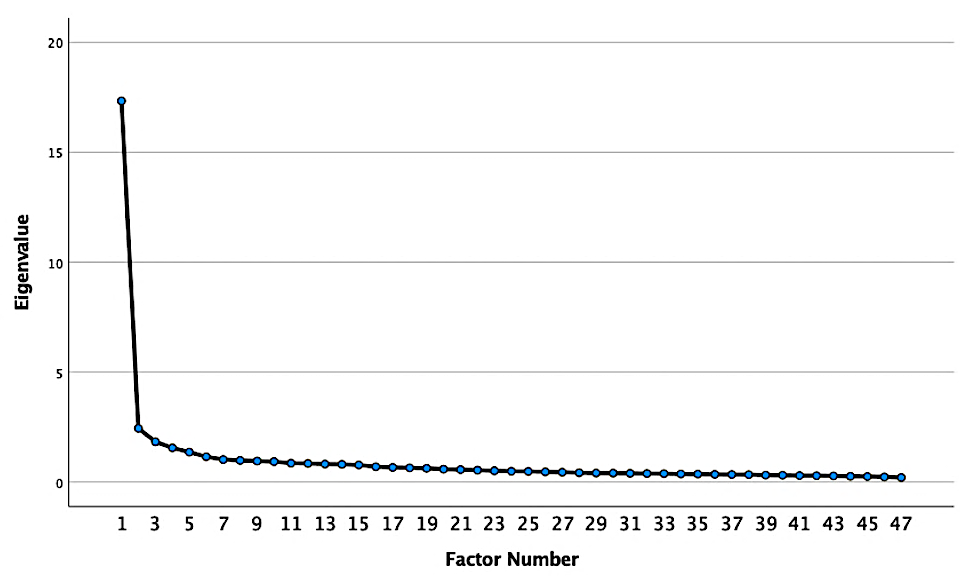


**eTable 12.**

*Total variance explained by Eigenvalues*

|  | Initial Eigenvalues | | |
| --- | --- | --- | --- |
| Factor | Total | % of Variance | Cumulative % |
| 1 | 17.36 | 36.94 | 36.94 |
| 2 | 2.45 | 5.21 | 42.15 |
| 3 | 1.83 | 3.90 | 46.05 |
| 4 | 1.56 | 3.32 | 49.36 |
| 5 | 1.36 | 2.89 | 52.26 |
| 6 | 1.15 | 2.44 | 54.70 |
| 7 | 01.03 | 2.18 | 56.88 |
| 8 | 0.98 | 2.10 | 58.98 |
| 9 | 0.96 | 2.03 | 61.01 |
| 10 | 0.93 | 1.98 | 62.98 |

*Note.* Only Eigenvalues from up to .90 are displayed.

**eTable 13.**

*Factor correlation matrix (seven-factors)*

| Factor | 1 | 2 | 3 | 4 | 5 | 6 | 7 |
| --- | --- | --- | --- | --- | --- | --- | --- |
| 1 | 1 | .78 | .65 | .43 | .59 | .50 | .16 |
| 2 | .78 | 1.00 | .58 | .39 | .64 | .43 | .14 |
| 3 | .65 | .58 | 1.00 | .39 | .65 | .30 | .06 |
| 4 | .43 | .39 | .39 | 1.00 | .46 | .42 | -.08 |
| 5 | .59 | .64 | .65 | .46 | 1.00 | .38 | -.11 |
| 6 | .50 | .43 | .30 | .42 | .38 | 1.00 | -.10 |
| 7 | .16 | .14 | .06 | -.08 | -.11 | -.10 | 1.00 |

**eTable 14.**

*Rotated factor loadings of seven-factor solution*

| Items | Factors | | | | | | |
| --- | --- | --- | --- | --- | --- | --- | --- |
|  | Identity disturbance | Paranoid fear of abandonment | (Para)suicidal behavior | Impulsivity | Difficulty controlling anger Anger | Dissociation | Affective instability |
| Not knowing what is actually important to you | .94 |  |  |  |  |  |  |
| Uncertainty about what your true standards and values are | .83 |  |  |  |  |  |  |
| Uncertainty about what should life look like | .81 |  |  |  |  |  |  |
| Uncertainty who you are | .81 |  |  |  |  |  |  |
| The conviction that you're not able to deal with life on your own | .80 |  |  |  |  |  |  |
| Finding yourself a bad and unacceptable person | .75 |  |  |  |  |  |  |
| If others get to know me, they will leave | .73 |  |  |  |  |  |  |
| Feeling empty inside | .70 |  |  |  |  |  |  |
| Not knowing what friends or loved ones you want to have | .70 |  |  |  |  |  |  |
| Feelings that are unacceptable to you | .65 |  |  |  |  |  |  |
| Feelings of despair | (.58) |  | (.32) |  |  |  |  |
| Being different in various situations | .57 |  |  |  |  |  |  |
| Hating yourself, everybody and the world | .55 |  |  |  |  |  |  |
| Sudden anxieties, depressions or irritability | (.51) |  |  |  |  |  | .31 |
| Binge eating | (.40) |  |  |  |  |  |  |
| Not being able to remember important things | .34 |  |  |  |  |  |  |
| Impulsive spending of too much money that you cannot afford to spend | (.30) |  |  | .30 |  |  |  |
| Not dare to recognize bad sides of yourself |  |  |  |  |  |  |  |
| Not knowing whether you are attracted to women or men |  |  |  |  |  |  |  |
| Suddenly losing trust in other people |  | .89 |  |  |  |  |  |
| Feeling terribly disappointed in someone you first admired or loved |  | .78 |  |  |  |  |  |
| Distrusting others |  | .77 |  |  |  |  |  |
| Being very suspicious |  | .72 |  |  |  |  |  |
| Convicted that others treat me unfairly |  | .51 |  |  |  |  |  |
| Strong changes of feelings for others | (.31) | .43 |  |  |  |  |  |
| Fear that others leave | (.32) | .40 |  |  |  |  |  |
| Frantically trying to prevent others from leaving you |  | .33 |  |  |  |  |  |
| Being convicted that others have it in for your |  | .33 |  |  |  |  |  |
| Trying to kill yourself |  |  | .94 |  |  |  |  |
| Urge to kill yourself |  |  | .88 |  |  |  |  |
| Self-mutilation |  |  | .58 |  |  |  |  |
| Threat others that you will hurt/kill yourself |  |  | .52 |  |  |  |  |
| Act in life threatening ways |  |  | .41 | .35 |  |  |  |
| Losing senses, because you are convicted that others will leave you |  | .30 | (.38) |  |  |  |  |
| Drinking |  |  |  | .58 |  |  |  |
| Drug use |  |  |  | .54 |  |  |  |
| Acting on an impulsive sexual contact you later regretted |  |  |  | .40 |  |  |  |
| Reckless driving |  |  |  | .38 |  |  |  |
| Gambling |  |  |  | .37 |  |  |  |
| Shoplifting |  |  |  | .32 |  |  |  |
| Becoming so angry that you lose control and break things |  |  |  |  | .81 |  |  |
| Hitting others |  |  |  |  | .73 |  |  |
| Tantrums |  |  |  |  | .72 |  | .42 |
| Not feeling oneself anymore, like an outside observer of yourself, or experiencing yourself as in a movie or dream |  |  |  |  |  | .60 |  |
| Observing or experiencing the world around you totally differently so that it seems very odd or unreal to you |  |  |  |  |  | .59 |  |
| Not knowing anymore what you have done or where you are |  |  |  |  |  | .36 |  |
| Quick changes of mood |  |  |  |  |  |  | .48 |

*Note.* Only factor loadings above .30 are displayed. We put some factor loadings in brackets, as we did not find those items to perfectly match the other items that load high on that factor.

**eTable 15.**

*Rotated factor loadings of the nine-factor solution*

| Items | Factors | | | | | | | | |
| --- | --- | --- | --- | --- | --- | --- | --- | --- | --- |
|  | Identity distrubance | Paranoid ideation | Parasuicidal behaviors | Impulsivity | Difficulty controlling anger | Dissociative symptoms | Fear of abandonment | Affective instability | Low self-esteem |
| Not knowing what is actually important to you | .90 |  |  |  |  |  |  |  |  |
| Uncertainty about what your true standards and values are | .77 |  |  |  |  |  |  |  |  |
| Uncertainty who you are | .77 |  |  |  |  |  |  |  |  |
| Uncertainty about what should life look like | .76 |  |  |  |  |  |  |  |  |
| Not knowing what friends or loved ones you want to have | .68 |  |  |  |  |  |  |  |  |
| The conviction that you're not able to deal with life on your own | .66 |  |  |  |  |  |  |  |  |
| Feeling empty inside | .65 |  |  |  |  |  |  |  |  |
| Finding yourself a bad and unacceptable person | .52 |  |  |  |  |  |  |  | .53 |
| If others get to know me, they will leave | .50 |  |  |  |  |  |  |  | .46 |
| Being different in various situations | .51 |  |  |  |  |  |  |  |  |
| Feelings that are unacceptable to you | .48 |  |  |  |  |  |  |  | .36 |
| Feelings of despair | .47 |  |  |  |  |  |  |  |  |
| Sudden anxieties, depressions or irritability | .46 |  |  |  |  |  |  | .35 |  |
| Hating yourself, everybody and the world | .46 |  |  |  |  |  |  |  |  |
| Binge eating |  |  |  |  |  |  |  |  |  |
| Not being able to remember important things |  |  |  |  |  |  |  |  |  |
| Not knowing whether you are attracted to women or men |  |  |  |  |  |  |  |  |  |
| Suddenly losing trust in other people |  | .83 |  |  |  |  |  |  |  |
| Distrusting others |  | .75 |  |  |  |  |  |  |  |
| Being very suspicious |  | .68 |  |  |  |  |  |  |  |
| Feeling terribly disappointed in someone you first admired or loved |  | .67 |  |  |  |  |  |  |  |
| Convicted that others treat me unfairly |  | .44 |  |  |  |  |  |  |  |
| Strong changes of feelings for others | .33 | .43 |  |  |  |  |  |  |  |
| Being convicted that others have it in for your |  | .30 |  |  |  |  |  |  |  |
| Trying to kill yourself |  |  | .95 |  |  |  |  |  |  |
| Urge to kill yourself |  |  | .95 |  |  |  |  |  |  |
| Self-mutilation |  |  | .55 |  |  |  |  |  |  |
| Threat others that you will hurt/kill yourself |  |  | .50 |  | .30 |  |  |  |  |
| Drinking |  |  |  | .58 |  |  |  |  |  |
| Drug use |  |  |  | .53 |  |  |  |  |  |
| Reckless driving |  |  |  | .43 |  |  |  |  |  |
| Acting on an impulsive sexual contact you later regretted |  |  |  | .39 |  |  |  |  |  |
| Act in life threatening ways |  |  |  | .38 |  |  |  |  |  |
| Gambling |  |  |  | .37 |  |  |  |  |  |
| Shoplifting |  |  |  | .32 |  |  |  |  |  |
| Impulsive spending of too much money that you cannot afford to spend |  |  |  | .31 |  |  |  |  |  |
| Becoming so angry that you lose control and break things |  |  |  |  | .70 |  |  |  |  |
| Hitting others |  |  |  |  | .69 |  |  |  |  |
| Tantrums |  |  |  |  | .60 |  |  | .48 |  |
| Not feeling oneself anymore, like an outside observer of yourself, or experiencing yourself as in a movie or dream |  |  |  |  |  | .66 |  |  |  |
| Observing or experiencing the world around you totally differently so that it seems very odd or unreal to you |  |  |  |  |  | .65 |  |  |  |
| Not knowing anymore what you have done or where you are |  |  |  |  |  | .40 |  |  |  |
| Frantically trying to prevent others from leaving you |  |  |  |  |  |  | .79 |  |  |
| Fear that others leave |  |  |  |  |  |  | .62 |  |  |
| Losing senses, because you are convicted that others will leave you |  |  |  |  |  |  | .61 |  |  |
| Quick changes of mood | .30 |  |  |  |  |  |  | .53 |  |
| Not dare to recognize bad sides of yourself |  |  |  |  |  |  |  |  | .39 |

*Note.* Only factor loadings above .30 are displayed.

**eTable 16.**

*Factor correlation matrix (nine-factors)*

| Factor | 1 | 2 | 3 | 4 | 5 | 6 | 7 | 8 | 9 |
| --- | --- | --- | --- | --- | --- | --- | --- | --- | --- |
| 1 | 1.00 | .71 | .55 | .41 | .44 | .55 | .62 | .37 | .55 |
| 2 | .71 | 1.00 | .48 | .39 | .50 | .51 | .62 | .36 | .55 |
| 3 | .55 | .48 | 1.00 | .41 | .54 | .42 | .63 | .34 | .68 |
| 4 | .41 | .39 | .41 | 1.00 | .45 | .47 | .37 | .10 | .36 |
| 5 | .44 | .50 | .54 | .45 | 1.00 | .44 | .51 | .07 | .48 |
| 6 | .55 | .51 | .42 | .47 | .44 | 1.00 | .38 | .09 | .42 |
| 7 | .62 | .62 | .63 | .37 | .51 | .38 | 1.00 | .39 | .59 |
| 8 | .37 | .36 | .34 | .10 | .07 | .09 | .39 | 1.00 | .42 |
| 9 | .55 | .55 | .68 | .36 | .48 | .42 | .59 | .42 | 1.00 |

**List of abbreviations**

BPD: Borderline Personality Disorder

BPDCL: Borderline Personality Disorder Checklist

SCL-90: Symptom Checklist-90 revised

DSM: Diagnostic and Statistical Manual of Mental Disorders

BPDSI: Borderline Personality Disorder Severity Index-IV

SCID-II: Structured Clinical Interview for DSM-IV Personality Disorders

SCID-I: Structured Clinical Interview for DSM-IV Disorders

ADHD: Attention Deficit Hyperactivity Disorder

#BPD criteria= Number of Borderline Personality Disorder criteria, according to SCID-II

BSI: Brief Symptom Inventory-53

WSAS: Work and Social Adjustment Scale

IPO: Inventory of Personality Organization

WHOQoL: World Health Organization Quality of Life Questionnaire

WCCL: Dialectical Behavior Therapy Ways of Coping Checklist

EuroQoL: European Quality of Life Scale

EQ-5D-3L= EuroQoL index

Q: question

N: sample size

n: frequency

Min: Minimum score

Max: Maximum score

SD=: standard deviation

X^2^ : Chi Square statistic

df: degree of freedom

P: p-value

F: Welch ANOVA F-statistic

PD: personality disorder

Other PD: personality disorder other than Borderline Personality Disorder

OCPD: Obsessive-compulsive Personality Disorder

**References**

Arntz, A., Jacob, G. A., Lee, C. W., Brand-de Wilde, O. M., Fassbinder, E., Harper, R. P., . . . Ruths, F. A. (2022). Effectiveness of predominantly group schema therapy and combined individual and group schema therapy for borderline personality disorder: A randomized clinical trial. *JAMA psychiatry, 79*(4), 287-299.

Arntz, A., van den Hoorn, M., Cornelis, J., Verheul, R., van den Bosch, W. M., & de Bie, A. J. (2003). Reliability and validity of the borderline personality disorder severity index. *Journal of Personality Disorders, 17*(1), 45-59.

Assmann, N., Schaich, A., Arntz, A., Wagner, T., Herzog, P., Alvarez-Fischer, D., . . . Hüppe, M. (2024). The effectiveness of dialectical behavior therapy compared to schema therapy for borderline personality disorder: A randomized clinical trial. *Psychotherapy and psychosomatics, 93*(4), 249-263.

Berghuis, H., Kamphuis, J. H., Boedijn, G., & Verheul, R. (2009). Psychometric properties and validity of the Dutch Inventory of Personality Organization (IPO-NL). *Bulletin of the Menninger Clinic, 73*(1), 44-60.

Brooks, R. (1996). EuroQol: the current state of play. *Health policy, 37*(1), 53-72.

Calvo, N., Valero, S., Arntz, A., Andión, Ó., Matalí, J., Navascues, V., . . . Ferrer, M. (2018). Validation of the Spanish version of the Borderline Personality Disorder Checklist (BPD Checklist) in a sample of BPD patients: Study of psychometric properties. *The European Journal of Psychiatry, 32*(1), 26-35.

Derogatis, L. R. (1992). SCL-90-R: Administration, scoring & procedures manual-II for the (revised) version and other instruments of the psychopathology rating scale series. *Clinical Psychometric Research.*, 1-16.

Derogatis, L. R. (1993). *Brief Symptom Inventory: Administration, scoring, and procedures manual*: National Computer Systems (NCS).

Derogatis, L. R., & Melisaratos, N. (1983). The brief symptom inventory: an introductory report. *Psychological medicine, 13*(3), 595-605.

Dorman, P., Slattery, J., Farrell, B., Dennis, M., & Sandercock, P. (1998). Qualitative comparison of the reliability of health status assessments with the EuroQol and SF-36 questionnaires after stroke. *Stroke, 29*(1), 63-68.

First, M. B. (1997). Structured Clinical Interview for DSM-IV Axis I Disorders (SCID-I), Clinician Version (Administration Booklet). American Psychiatric Publishing, Inc. *(No Title)*.

First, M. B., & Gibbon, M. (2004). The structured clinical interview for DSM-IV axis I disorders (SCID-I) and the structured clinical interview for DSM-IV axis II disorders (SCID-II).

Giesen-Bloo, J., Arntz, A., & Schouten, E. (2006). The borderline personality disorder checklist: psychometric evaluation and factorial structure in clinical and nonclinical samples. *Crossing Borders: Theory, Assessment and Treatment in Borderline Personality Disorder by Giesen-Bloo J, 2006*, 85-102.

Group, W. (1998). Development of the World Health Organization WHOQOL-BREF quality of life assessment. *Psychological medicine, 28*(3), 551-558.

<https://euroqol.org/information-and-support/resources/value-sets/>, A. f. ([cited 2023 Feb 4]). Value sets [Internet]. EuroQol. .

Kernberg, O. F., & Clarkin, J. F. (1995). Inventory of Personality Organization. *PsycTESTS Dataset*.

Mataix-Cols, D., Cowley, A. J., Hankins, M., Schneider, A., Bachofen, M., Kenwright, M., . . . Marks, I. M. (2005). Reliability and validity of the Work and Social Adjustment Scale in phobic disorders. *Comprehensive psychiatry, 46*(3), 223-228.

Mundt, J. C., Marks, I. M., Shear, M. K., & Greist, J. M. (2002). The Work and Social Adjustment Scale: a simple measure of impairment in functioning. *The British Journal of Psychiatry, 180*(5), 461-464.

Neacsiu, A. D., Rizvi, S. L., Vitaliano, P. P., Lynch, T. R., & Linehan, M. M. (2010). The dialectical behavior therapy ways of coping checklist: development and psychometric properties. *Journal of clinical psychology, 66*(6), 563-582.

Prinz, U., Nutzinger, D. O., Schulz, H., Petermann, F., Braukhaus, C., & Andreas, S. (2013). Comparative psychometric analyses of the SCL-90-R and its short versions in patients with affective disorders. *BMC psychiatry, 13*, 1-9.

Richetin, J., Preti, E., Costantini, G., & De Panfilis, C. (2017). The centrality of affective instability and identity in Borderline Personality Disorder: Evidence from network analysis. *PloS one, 12*(10), e0186695.

Smits, I. A., Timmerman, M. E., Barelds, D. P., & Meijer, R. R. (2014). The Dutch symptom checklist-90-revised. *European Journal of Psychological Assessment*.

Stein, A. T., Hearon, B. A., Beard, C., Hsu, K. J., & Björgvinsson, T. (2016). Properties of the dialectical behavior therapy ways of coping checklist in a diagnostically diverse partial hospital sample. *Journal of clinical psychology, 72*(1), 49-57.

Tsang, H. H. L., Cheung, J. P. Y., Wong, C. K. H., Cheung, P. W. H., Lau, C. S., & Chung, H. Y. (2019). Psychometric validation of the EuroQoL 5-dimension (EQ-5D) questionnaire in patients with spondyloarthritis. *Arthritis Research & Therapy, 21*, 1-14.

Van Asselt, A., Dirksen, C., Arntz, A., Giesen-Bloo, J., & Severens, J. (2009). The EQ-5D: A useful quality of life measure in borderline personality disorder? *European psychiatry, 24*(2), 79-85.

Wetzelaer, P., Farrell, J., Evers, S. M., Jacob, G. A., Lee, C. W., Brand, O., . . . Harper, R. P. (2014). Design of an international multicentre RCT on group schema therapy for borderline personality disorder. *BMC psychiatry, 14*(1), 319.
