# Supplementary material for: Psychometric Evaluation of the Borderline Personality Disorder Checklist
Source: Int J Methods Psychiatr Res. 2025 Sep 25;34(3):e70029. doi: 10.1002/mpr.70029 (PMC12461754; doi:10.1002/mpr.70029)
Supplement: Supplementary file 1 — Supporting Information S1 [file MPR-34-e70029-s001.zip › Wiley_SM2-Dutch .docx]

**Supplementary material for the Dutch dataset (SM2)**

**eAppendix 1.** Descriptives of the Dutch sample

**eTable 1.** Demographics

**eTable 2.** Clinical information

**eAppendix 2.** Item analyses of the Dutch BPDCL

**eTable 3.** Results of Shapiro Wilk test

**eTable 4**. Results of individual item analyses

**eAppendix 3.** Reliability analyses of the Dutch BPDCL

**eTable 5.** Reliability coefficients of each BPDCL subscales (total sample)

**eTable 6.** Reliability coefficients of each BPDCL subscales (BPD sample)

**eAppendix 4**. Convergent validity of the Dutch BPDCL

**eTable 7.** Means and standard deviations of each instrument

**eTable 8.** BPDCL and BPDSI

**eTable 9.** BPDCL and EuroQoL, WSAS, IPO and the WCCL

**eTable 10.** BPDCL and BSI-53

**eTable 11.** BPDCL *WHOQoL*

**eAppendix 5**. Known-groups validity of Dutch BPDCL

**eTable 12.** Results of Kruskal-Wallis test

This supplementary material has been provided by the authors to give readers additional information about their work. **eAppendix 1.** Descriptives of the Dutch sample

The Dutch sample consisted of 603 participants, 404 BPD patients, 55 other PD patients, 57 Axis I disorder patients and 87 healthy controls. The data was drawn from two different studies, conducted by Bloo et al. and Arntz et al.  The age ranged from 18 to 60, with a mean

age of 33.58 (SD=10.38). The majority identified as female, was single, came from Western Europe and currently received sickness benefits. Comorbidities with other disorders were quite high. Table 1 gives a good overview over the demographics and clinical information based on the clinical group (BPD, other PD, Axis I disorder or healthy patient). It is important to note that the specific clinical information (which exact diagnosis) of 109 Dutch participants was not provided by the researchers. However, the variable *clinical group* was available. Thus, each participant was assigned to a specific clinical group (BPD, other PD, Axis I disorder or healthy patient) and the data could be used for our analyses (we did not control for comorbidities). Chi-square tests were applied for the categorical variables (gender, marital status, ethnicity and jobstatus). The four groups differed significantly with respect to gender ((χ² (3)=43.71, p<.001), marital status (χ² (12)=33.395, p<.001) and jobstatus (χ² (21) = 113.872, p<.001). The four groups did not differ with respect to ethnicity ((χ² (15)=10.772, p=.769). One way ANOVA was performed on the mean age across the four groups. Unequal variances must be assumed (Levene’s statistic(3,593)=27.661, p<.001). Welch ANOVA indicates that the four groups differ with respect to age (F(3, 114.657)=4.649, p=.004). Games-Howell post hoc tests suggest that the BPD group was slightly younger than the other PD group (p=.001). The other groups did not differ significantly from one another (p>.05).

**eTable 1.**

*Sociodemographic data of the Dutch sample*

|  | BPD  N=404 | Other PD  N=55 | Axis I  N=57 | HC  N=87 |
| --- | --- | --- | --- | --- |
|  |  |  |  |  |
|  | n | n | n | n |
| Gender |  |  |  |  |
| Female | 346 (85.64) | 29 (54.72) | 33 (62.26) | 59 (67.82) |
| Male | 58 (14.36) | 24 (45.28) | 20 (37.74) | 28 (32.18) |
| Marital status |  |  |  |  |
| Single | 227 (56.47) | 20 (37.74) | 19 (35.85) | 34 (39.53) |
| Married or lasting relationship | 151 (37.56) | 28 (52.83) | 28 (52.83) | 45 (52.33) |
| Separated divorced | 11 (2.74) | 3 (5.66) | 4 (7.55) | 5 (5.81) |
| Widowed | 0 (0) | 1 (1.89) | 0 (0) | 2 (2.33) |
| Other | 13 (3.23) | 1 (1.89) | 2 (3.77) | 0 (0) |
| Ethnicity |  |  |  |  |
| Western Europe | 381 (94.78) | 55 (100) | 57 (100) | 87 (100) |
| Eastern Europe | 2 (0.50) | 0 (0) | 0 (0) | 0 (0) |
| South America | 3 (0.75) | 0 (0) | 0 (0) | 0 (0) |
| Africa | 4 (1.00) | 0 (0) | 0 (0) | 0 (0) |
| Asia and middle east | 8 (2.00) | 0 (0) | 0 (0) | 0 (0) |
| Mixed | 4 (1.00) | 0 (0) | 0 (0) | 0 (0) |
| Employment |  |  |  |  |
| Homemaker | 32 (7.98) | 10 (18.87) | 2 (3.77) | 13 (15.12) |
| Student | 34 (8.48) | 7 (13.21) | 5 (9.43) | 29 (33.72) |
| Sickness benefits | 167 (41.65) | 16 (30.19) | 28 (52.83) | 6 (6.98) |
| Working | 93 (23.19) | 19 (35.85) | 14 (26.42) | 30 (34.88) |
| Unemployed | 36 (8.98) | 0 (0) | 0 (0) | 3 (3.49) |
| Retired | 6 (1.50) | 0 (0) | 4 (7.55) | 4 (4.65) |
| No legal income | 1 (0.25) | 0 (0) | 0 (0) | 0 (0) |
| Other | 32 (7.98) | 1 (1.89) | 0 (0) | 1 (1.16) |
|  | Mean (SD) | Mean (SD) | Mean (SD) | Mean (SD) |
| Age | 32.51 (8.91) | 38.19 (11.94) | 34.13 (10.28) | 35.38 (14.23) |

*Note.* BPD = Borderline Personality Disorder, Axis I=Axis I disorder patient, HC= Healthy controls. Only valid percentages are reported. Missings can be found in the output file.

**eTable 2.**

*Clinical information of the Dutch sample*

|  | BPD  N=404 | Other PD  N=55 | Axis I disorder  N=57 |
| --- | --- | --- | --- |
|  |  |  |  |
|  | n | n | n |
| Axis I disorder |  |  |  |
| Affective | 224 (70.22) | 12 (38.71) | 10 (40) |
| Anxiety | 118 (56.73) | 18 (58.06) | 17 (68.00) |
| Substance use | 109 (51.66) | 1 (3.23) | 2 (8.00) |
| Eating | 52 (34.67) | 0 (0) | 0 (0) |
| Other | 107 (51.44) | 2 (6.45) | 3 (12.00) |
| Axis II disorder |  |  |  |
| Avoidant PD | 73 (19.84) | 11 (35.48) | 0 (0) |
| Dependent PD | 23 (6.32) | 3 (9.68) | 0 (0) |
| OCPD | 34 (9.34) | 21 (67.74) | 0 (0) |
| Paranoid PD | 38 (10.35) | 0 (0) | 0 (0) |
| Schizotyp PD | 2 (0.54) | 0 (0) | 0 (0) |
| Borderline PD | 404 (100) | 0 (0) | 0 (0) |
| Unspecified PD | 8 (2.18) | 0 (0) | 0 (0) |

*Note.* All participants were outpatients. PD= Personality disorder, OCPD= Obsessive Compulsive Personality Disorder. Specific clinical information of 109 participants was missing, due to unavailability of the data.

**eAppendix 2.** Item analyses of the Dutch BPDCL

According to the Shapiro-Wilk’s test and the visual inspection of the data, the assumption of normality of the data is not met (p<.001, see Table 3). Item means range from 1.07 (item 8) to 3.33 (item 15), with a mean of 2.07 for the Dutch sample. Some items were centered at one end of the response scale (e.g. Item 41 with a mean of 1.25). All item responses, ranging from 1 to 5, were selected for each item by the Dutch sample.  The mean inter item correlation is 0.32, which is within the predefined range of 0.20 to 0.40. The variance of the mean-inter-item correlation (s²=.03) is rather small. Thus, the individual inter-item correlations do not vary strongly. Item 7, 8, 17, 35 and 41 have corrected item total correlations below 0.30. Cronbach’s Alpha if those items were deleted does not differ from the initial Cronbach’s Alpha of the total scale (Cronbach’s Alpha=0.96).  Thus, this small deviation from .3 can be ignored, as the scale reliability is not affected by those items. The total scale mean was 97.25 (SD=32.83).  The highest scale score was 185 and the lowest was 47. The results of the item analysis are presented in Table 4.

Reliability coefficients for the Dutch sample can be found in Table 5. Reliability coefficients of each subscale were higher than the predefined value of 0.70, except for the *Impulsivity* subscale (Cronbach’s Alpha= 0.65). Guttman’s Lamda2 was slightly higher than Cronbach’s Alpha (e.g. *Impulsivity* .68). The reliability coefficients for the BPD only sample can be seen in Table 6. Again, only the *Impulsivity* scale did not reach the predefined coefficient of .70 (Cronbach’s Alpha =.55). The Cronbach’s Alpha of the total scale was 0.93, thus slightly lower than for the total Dutch sample.

**eTable 3.**

*Test of normality of the Dutch BPDCL*

| Shapiro-Wilk | | | |
| --- | --- | --- | --- |
|  | Statistic | df | Sig. |
| item 1 | .83 | 602 | <.001 |
| item 2 | .89 | 602 | <.001 |
| item 3 | .84 | 602 | <.001 |
| item 4 | .81 | 602 | <.001 |
| item 5 | .49 | 602 | <.001 |
| item 6 | .54 | 602 | <.001 |
| item 7 | .45 | 602 | <.001 |
| item 8 | .20 | 602 | <.001 |
| item 9 | .69 | 602 | <.001 |
| item 10 | .88 | 602 | <.001 |
| item 11 | .88 | 602 | <.001 |
| item 12 | .65 | 602 | <.001 |
| item 13 | .87 | 602 | <.001 |
| item 14 | .80 | 602 | <.001 |
| item 15 | .88 | 602 | <.001 |
| item 16 | .86 | 602 | <.001 |
| item 17 | .45 | 602 | <.001 |
| item 18 | .88 | 602 | <.001 |
| item 19 | .89 | 602 | <.001 |
| item 20 | .76 | 602 | <.001 |
| item 21 | .86 | 602 | <.001 |
| item 22 | .62 | 602 | <.001 |
| item 23 | .56 | 602 | <.001 |
| item 24 | .55 | 602 | <.001 |
| item 25 | .89 | 602 | <.001 |
| item 26 | .50 | 602 | <.001 |
| item 27 | .76 | 602 | <.001 |
| item 28 | .42 | 602 | <.001 |
| item 29 | .79 | 602 | <.001 |
| item 30 | .86 | 602 | <.001 |
| item 31 | .61 | 602 | <.001 |
| item 32 | .81 | 602 | <.001 |
| item 33 | .87 | 602 | <.001 |
| item 34 | .88 | 602 | <.001 |
| item 35 | .20 | 602 | <.001 |
| item 36 | .90 | 602 | <.001 |
| item 37 | .61 | 602 | <.001 |
| item 38 | .82 | 602 | <.001 |
| item 39 | .86 | 602 | <.001 |
| item 40 | .86 | 602 | <.001 |
| item 41 | .42 | 602 | <.001 |
| item 42 | .86 | 602 | <.001 |
| item 43 | .87 | 602 | <.001 |
| item 44 | .79 | 602 | <.001 |
| item 45 | .77 | 602 | <.001 |

*Note*. df= degress of freedom, Sig.=Significance. Cases were

excluded listwise.

**eTable 4.**

*Item analysis of the Dutch BPDCL*

|  | Mean | SD | r_tot_ | α _if item was deleted_ |
| --- | --- | --- | --- | --- |
| item 1 | 2.18 | 1.25 | .48 | .96 |
| item 2 | 3.22 | 1.36 | .80 | .96 |
| item 3 | 2.21 | 1.21 | .64 | .96 |
| item 4 | 2.14 | 1.28 | .62 | .96 |
| item 5 | 1.29 | .67 | .32 | .96 |
| item 6 | 1.45 | .96 | .40 | .96 |
| item 7 | 1.29 | .74 | .30 | .96 |
| item 8 | 1.07 | .36 | .03 | .96 |
| item 9 | 1.66 | 1.01 | .59 | .96 |
| item 10 | 2.87 | 1.42 | .75 | .96 |
| item 11 | 3.20 | 1.42 | .77 | .96 |
| item 12 | 1.59 | .99 | .32 | .96 |
| item 13 | 2.80 | 1.46 | .70 | .96 |
| item 14 | 2.09 | 1.25 | .69 | .96 |
| item 15 | 3.33 | 1.39 | .74 | .96 |
| item 16 | 2.28 | 1.22 | .62 | .96 |
| item 17 | 1.32 | .82 | .19 | .96 |
| item 18 | 2.40 | 1.22 | .73 | .96 |
| item 19 | 2.68 | 1.35 | .71 | .96 |
| item 20 | 1.75 | .96 | .52 | .96 |
| item 21 | 2.51 | 1.40 | .72 | .96 |
| item 22 | 1.51 | .90 | .40 | .96 |
| item 23 | 1.43 | .87 | .41 | .96 |
| item 24 | 1.39 | .79 | .48 | .96 |
| item 25 | 2.91 | 1.41 | .78 | .96 |
| item 26 | 1.36 | .82 | .47 | .96 |
| item 27 | 1.91 | 1.18 | .58 | .96 |
| item 28 | 1.24 | .66 | .38 | .96 |
| item 29 | 2.11 | 1.33 | .44 | .96 |
| item 30 | 2.49 | 1.38 | .73 | .96 |
| item 31 | 1.52 | .95 | .51 | .96 |
| item 32 | 2.16 | 1.30 | .65 | .96 |
| item 33 | 2.42 | 1.31 | .71 | .96 |
| item 34 | 2.55 | 1.31 | .69 | .96 |
| item 35 | 1.08 | .39 | .13 | .96 |
| item 36 | 3.07 | 1.37 | .81 | .96 |
| item 37 | 1.55 | 1.01 | .47 | .96 |
| item 38 | 2.03 | 1.14 | .52 | .96 |
| item 39 | 2.40 | 1.29 | .70 | .96 |
| item 40 | 2.63 | 1.45 | .68 | .96 |
| item 41 | 1.25 | .69 | .26 | .96 |
| item 42 | 2.31 | 1.24 | .72 | .96 |
| item 43 | 2.67 | 1.44 | .71 | .96 |
| item 44 | 2.14 | 1.34 | .71 | .96 |
| item 45 | 2.03 | 1.29 | .61 | .96 |
| item 46 | 2.26 | 1.25 | .65 | .96 |
| item 47 | 1.43 | .84 | .42 | .96 |

*Note.* SD= Standard deviation, r_tot=_ corrected item total correlation,

α= Cronbach’s Alpha.

**eAppendix 3.** Reliability analyses of the Dutch BPDCL

**eTable 5.**

*Reliability coefficients of each BPDCL subscale for the Dutch sample (N=603)*

|  | Cronbach’s Alpha | Guttman Lamda2 | McDonald’sOmega |
| --- | --- | --- | --- |
| Fear of Abandonment | .86 | .88 | .88 |
| Interpersonal relationships | .79 | .79 | .80 |
| Identity disturbance | .88 | .89 | .89 |
| Impulsivity | .65 | .68 | .67 |
| Parasuicidal behaviour | .77 | .78 | .79 |
| Affective instability | .90 | .90 | .90 |
| Emptiness | - | - | - |
| Anger | .77 | .79 | .80 |
| Paranoid and dissociative behavior | .84 | .85 | .84 |
| Total scale | .96 | .97 | .97 |

*Note.* McDonal’s Omega should be interpreted with caution, as the assumption of normality was violated.

**eTable 6.**

*Reliability coefficients of each BPDCL subscale for the Dutch BPD sample (N=404)*

|  | Cronbach’s Alpha | Guttman Lamda2 | McDonald’sOmega |
| --- | --- | --- | --- |
| Fear of Abandonment | .80 | .82 | .81 |
| Interpersonal relationships | .71 | .71 | .72 |
| Identity disturbance | .81 | .82 | .82 |
| Impulsivity | .55 | .58 | .55 |
| Parasuicidal behaviour | .75 | .75 | .77 |
| Affective instability | .81 | .82 | .82 |
| Emptiness | - | - | - |
| Anger | .73 | .75 | .75 |
| Paranoid and dissociative behavior | .79 | .80 | .76 |
| Total scale | .93 | .94 | .93 |

*Note.* McDonal’s Omega should be interpreted with caution, as the assumption of normality was violated.

**eAppendix 4**. Convergent validity of the Dutch BPDCL

The means and standard deviations of the other psychological instruments are presented in Table 7. Convergent validity was assessed by correlating the BPDCL with the other instruments. Spearman’s Rho correlations are presented in Table 7 to 12.

The BPDCL total score correlated strongly with the BPDSI total score (.75). BPDCL Affective instability correlated the most with the BPDSI total score (.71). All BPDCL subscales correlated strongly (>.55) with their corresponding BPDSI subscales. The highest correlation was found between the two *Impulsivity* subscales (.78). Overall, the correlations were very high across the two instruments.

The EuroQoL index (EQ-5D-3L) did not correlate strongly with the BPDCL (-.43). Only the BPDCL *Affective instability* scale reached an acceptable correlation of .52 with the index score. Thus, scoring high on *Affective instability* is associated with lower health-related quality of life. The WSAS total score (.25) and the WCCL subscales *Blaming others* (.29) and *Skill use* (.05) did not correlate adequately with the BPDCL. WCCL *Dysfunctional coping* was moderately correlated with *Affective instability* (.53). The BPDCL correlated strongly with the IPO subscales. IPO *identity diffusion* correlated strongly with its corresponding BPDCL scale *Identity disturbance* (.59). The *fear of Abandonment* (BPDCL) was highly correlated with the IPO *Primitive psychological defenses* scale (.53). The *Paranoid* BPDCL scale was strongly correlated with the IPO *Alternative reality testing* scale (.57).

The BSI-53 correlated very strongly with the BPDCL (.82). The BSI *Psychotic* scale had the highest correlation with the BPDCL total scale (.73). The BSI *Somatization* correlated the least with the BPDCL (.47). The BPDCL *Affective instability* scale correlated the most with the BSI scales *Depression* (.71) and *Anxiety* (.68). The BPDCL *Anger* scale correlated the strongest with its corresponding BSI scale *Hostility* (.76). The Paranoid subscales correlated strongly, too (.73). The SCL total score correlated very strongly with the BPDCL (.90).

Finally, the BPDCL total score did not correlate strongly with the WHO scales *Psychological, Physical, Environmental and Social* quality of life (<.45). Only *Affective instability* correlated acceptably with the *Psychological* domain of the WHO (.45). The BPDCL did correlate highly with the WHO domains *self-esteem* (-.50), *Positive feelings* (-.45) and *Negative feelings* (.59). Scoring high on *Identity disturbance* (BPDCL) was associated with low scores on the *self-esteem* scale of the WHO (-.53). The WHO *Negative feelings* scale was the most associated with *Affective instability* (.71). The WHO scale *Positive feelings* was acceptably and negatively correlated with *Affective instability and Emptiness* scales of the BPDCL (.51 for both scales).

**eTable 7.**

*Means and standard deviations of each instrument filled out by the Dutch sample*

|  | N | Min | Max | Mean | SD |
| --- | --- | --- | --- | --- | --- |
| BPDSI- Abandonment | 512 | 0.00 | 8.43 | 2.55 | 2.02 |
| BPDSI- Interpersonal  relationships | 512 | 0.00 | 6.25 | 1.91 | 1.51 |
| BPDSI- Identity disturbance | 512 | 0.00 | 9.69 | 3.60 | 2.61 |
| BPDSI- Impulsivity | 512 | 0.00 | 5.27 | 1.10 | 1.04 |
| BPDSI- Parasuicidal b. | 512 | 0.00 | 6.31 | .67 | 0.94 |
| BPDSI- Affective instability | 512 | 0.00 | 10.00 | 5.60 | 3.33 |
| BPDSI- Emptiness | 512 | 0.00 | 10.00 | 4.91 | 3.11 |
| BPDSI- Anger | 512 | 0.00 | 8.67 | 2.47 | 2.02 |
| BPDSI- Dissoziation | 512 | 0.00 | 9.25 | 1.76 | 1.80 |
| BPDSI- Sum Score | 531 | 0.00 | 63.02 | 24.78 | 14.20 |
| EQ-5D-3L reverse crosswalk | 266 | -0.21 | 0.95 | 0.54 | 0.21 |
| Total score of WSAS | 265 | 2.00 | 39.00 | 22.64 | 8.04 |
| IPO Identity dissusion | 78 | 20.00 | 78.00 | 54.44 | 12.70 |
| IPO Primitive Psychological d. | 78 | 17.00 | 66.00 | 40.77 | 10.81 |
| IPO Alternative reality testing | 78 | 13.00 | 61.00 | 26.76 | 10.13 |
| Somatization BSI | 266 | 0.00 | 4.00 | 1.25 | 0.84 |
| Obsessive BSI | 266 | 0.00 | 4.00 | 2.04 | 0.89 |
| Insecurity BSI | 266 | 0.00 | 4.00 | 1.94 | 0.97 |
| Depressive BSI | 266 | 0.00 | 4.00 | 2.12 | 0.99 |
| Anxiety BSI | 266 | .17 | 4.00 | 1.74 | 0.92 |
| Hostility BSI | 266 | 0.00 | 4.00 | 1.41 | 0.96 |
| Phobic anxiety BSI | 266 | 0.00 | 4.00 | 1.29 | 0.95 |
| Paranoid BSI | 266 | 0.00 | 4.00 | 1.68 | 0.91 |
| Psychoticism BSI | 266 | 0.00 | 4.00 | 1.62 | 0.76 |
| Sumscore BSI | 266 | 15.00 | 202.00 | 88.76 | 37.25 |
| WCCL Skill use | 82 | 0.61 | 2.79 | 1.60 | 0.42 |
| WCCL Dyfunctional coping | 82 | 0.87 | 3.00 | 2.25 | 0.47 |
| WCCL Blaming others | 82 | 0.17 | 3.00 | 1.52 | 0.73 |
| Self-esteem WHO | 266 | 1.00 | 4.00 | 2.32 | 0.63 |
| Negative feelings WHO | 266 | 2.00 | 5.00 | 3.74 | 0.66 |
| Positive feelings WHO | 266 | 1.00 | 4.25 | 2.42 | 0.65 |
| Physical scale WHO | 266 | 10.71 | 75.00 | 39.88 | 11.17 |
| Psychological scale WHO | 266 | 16.67 | 75.00 | 40.05 | 10.99 |
| Social scale WHO | 266 | 0.00 | 100.00 | 43.48 | 22.00 |
| Environment WHO | 266 | 12.50 | 87.50 | 52.91 | 14.47 |

*Note.* SD= Standard deviation, N= Frequency, BPDSI = Borderline Personality Severity Index, EQ-5D-3 L = EuroQoL index, WSAS= Work Social Adjustment Scale, IPO= Inventory of Personality Organization,  BSI = Brief Symptom Inventory -53, WCCL= Ways of Coping Checklist, WHO= World Health Organiazion.

**eAppendix 5**. Known-groups validity of Dutch BPDCL

### ***Known-groups validity***

The Kruskal-Wallis test was significant, indicating that the diagnostic groups differed on the BPDCL total score (**χ²** (3, n=602)=287.975, p=.000). Post hoc tests with Bonferroni correction were applied to investigate which groups differed from one another (see Table 12). When comparing the BPDCL total score medians, BPD patients scored higher on the total scale (Md=111, n=403) compared to the other groups. Other PD (Md=85, n=55) and Axis I disorder patients (Md=71, n=57) did not differ significantly from one another (p=.148). However, the trend was going in the correct direction, with other PD sample scoring higher than Axis I disorder patients (not significantly). The Healthy controls (Md=49, n=87) scored the lowest on the BPDCL total score and differed significantly from the other groups (p<.05). The Dutch BPDCL total score seems to differentiate well between BPD, other psychopathology, and healthy controls.

The Kruskal-Wallis tests were significant for each subscale of the Dutch BPDCL, indicating that the groups differ on each scale (p<.05). See Table 12 for post hoc comparisons.

|  | BPDSI Abandonment | BPDSI  Relation | BPDSI Identity | BPDSI Impulsive | BPDSI  Parasuicidal | BPDSI  Affective | BPDSI  Emptiness | BPDSI Anger | BPDSI  Dissociative | BPDSI  Total |
| --- | --- | --- | --- | --- | --- | --- | --- | --- | --- | --- |
| BPDCL subscales |  |  |  |  |  |  |  |  |  |  |
| Abandonment | **.69**** | .54** | .59** | .53** | .54** | .58** | .58** | .47** | .56** | **.69**** |
| Interpersonal relationships | .53** | **.55**** | .55** | .52** | .46** | .52** | .51** | .46** | .54** | **.62**** |
| Identity disturbance | .53** | .52** | **.67**** | .54** | .53** | .57** | .58** | .46** | .57** | **.68**** |
| Impulsivity | .45** | .43** | .47** | **.78**** | .39** | .45** | .46** | .40** | .42** | **.55**** |
| Parasuicidal behavior | .38** | .34** | .42** | .39** | **.68**** | .41** | .37** | .37** | .41** | **.49**** |
| Affective instability | .57** | .54** | .58** | .57** | .60* | **.64**** | .60** | .52** | .58** | **.71**** |
| Emptiness | .52** | .48** | .52** | .50** | .51** | .58** | **.61**** | .45** | .49** | **.63**** |
| Anger | .50** | .52** | .53** | .45** | .53** | .57** | .48** | **.69**** | .50** | **.65**** |
| Paranoid ideation | .51** | .55** | .59** | .50** | .54** | .58** | .55** | .51** | **.69**** | **.69**** |
| Total score | **.62**** | **.58**** | **.65**** | **.62**** | **.60**** | **.63**** | **.61**** | **.55**** | **.63**** | **.75**** |

**Table 8**

*Spearman’s Rho correlations of the Dutch BPDCL and BPDSI*

*Note.* ** Correlation is significant at the 0.01 level (two-tailed). * Correlation is significant at the 0.05 level (two-tailed). Correlations without a star are not significant. BPDCL= Borderline Personality Disorder Checklist, BPDSI= Borderline Personality Disorder Severity Index.

**Table 9**

*Spearman's Rho correlations of the Dutch BPDCL and the EuroQoL, WSAS, IPO and the WCCL*

|  | EQ-5D-3L | WSAS  Total score | IPO  Identity d. | IPO  Defenses | IPO  Reality t. | WCCL  Skill use | WCCL  Dysfunct. | WCCL  Blaming |
| --- | --- | --- | --- | --- | --- | --- | --- | --- |
| BPDCL subscales |  |  |  |  |  |  |  |  |
| Abandonment | -.27** | .21** | .51** | **.53**** | .33** | .04 | .30** | .37** |
| Interpersonal relationships | -.29** | .17** | .47** | .48** | .17 | .05 | .45** | .33** |
| Identity disturbance | -.36** | .23** | **.59**** | .49** | .29** | .11 | .46** | .23** |
| Impulsivity | -.10 | .06 | .37** | .35** | .40** | -.02 | .33** | .03 |
| Parasuicidal behavior | -.34** | .12* | .34** | .35** | .40** | .01 | .40** | .06 |
| Affective instability | **-.52**** | .31** | .46** | .49** | .32** | -.02 | **.53**** | .24* |
| Emptiness | -.41** | .24** | .37** | .44** | .29** | -.16 | .33** | .11 |
| Anger | -.25** | .22** | .51** | .49** | .52** | -.02 | .34** | .35** |
| Paranoid ideation | -.42** | .20** | .40** | .43** | **.57**** | .10 | .44** | .17 |
| Total score | **-.43**** | **.25**** | **.62**** | **.62**** | **.51**** | **.05** | **.50**** | **.29**** |

*Note.* ** Correlation is significant at the 0.01 level (two-tailed). * Correlation is significant at the 0.05 level (two-tailed). Correlations without a star are not significant. Important findings are highlighted (bold and underlined). EQ-5D-3L= EuroQoL index, WSAS= Work and Social Adjustment scale, IPO= Inventory of Personality Organization, Identity d. =Identity dissfusion, Defenses=Primitive Psychological Defenses, Reality t.= Alternative Reality Testing, WCCL= DBT- Ways of coping Checklist, Dysfunct.=Dysfunctional Coping, Blaming= Blaming others.

**Table 10**

*Spearman's Rho correlations of the Dutch BPDCL and the BSI-53*

|  | BSI Somatization | BSI Obsession | BSI Insecurity | BSI Depression | BSI  Anxiety | BSI  Hostility | BSI Phobia | BSI Paranoid | BSI Psychotic | BSI Total |
| --- | --- | --- | --- | --- | --- | --- | --- | --- | --- | --- |
| BPDCL subscales |  |  |  |  |  |  |  |  |  |  |
| Abandonment | .30** | .43** | .49** | .51** | .53** | .46** | .50** | .55** | .56** | .61** |
| Interpersonal relationships | .35** | .42** | .55** | .48** | .48** | .44** | .45** | .62** | .58** | .61** |
| Identity disturbance | .38** | .52** | **.57**** | .64** | .57** | .44** | .56** | .58** | .68** | .70** |
| Impulsivity | .17** | .28** | .26** | .33** | .34** | .26** | .27** | .27** | .37** | .34** |
| Parasuicidal behavior | .29** | .28** | .35** | .54** | .44** | .32** | .39** | .31** | .46** | .50** |
| Affective instability | .49** | .57** | **.58**** | **.71**** | .**68**** | .59** | .54** | .51** | .65** | .78** |
| Emptiness | .41** | .43** | .44** | **.65**** | .45** | .37** | .45** | .34** | .55** | .60** |
| Anger | .29** | .33** | .47** | .47** | .45** | .**76**** | .37** | .51** | .42** | .57** |
| Paranoid ideation | .48** | .55** | **.60**** | .54** | .58** | .52** | .56** | **.73**** | .62** | .73** |
| Total score | **.47**** | **.58**** | **.66**** | **.70**** | **.68**** | **.63**** | **.62**** | **.70**** | **.73**** | **.82**** |

*Note.* ** Correlation is significant at the 0.01 level (two-tailed). * Correlation is significant at the 0.05 level (two-tailed). Correlations without a star are not significant. Important findings are highlighted (bold and underlined). BSI= Brief Symptom Inventory-53.

**Table 11**

*Spearman's Rho correlations of the BPDCL and the WHOQoL*

|  | SCL  Total | WHO  Self-esteem | WHO Negative f. | WHO  Positive f. | WHO Physical | WHO Psychological | WHO   Social | WHO Environment |
| --- | --- | --- | --- | --- | --- | --- | --- | --- |
| BPDCL subscales |  |  |  |  |  |  |  |  |
| Abandonment | .84 | -.38** | .45** | -.25** | -.14* | -.25** | -.21** | -.20** |
| Interpersonal relationships | .76 | -.35** | .36** | -.31** | -.08 | -.30** | -.35** | -.17** |
| Identity disturbance | .81 | **-.53**** | .49** | -.46** | -.20** | -.44** | -.29** | -.27** |
| Impulsivity | .64 | -.20** | .25** | -.15* | -.06 | -.18** | -.17** | -.14* |
| Parasuicidal behavior | .65 | -.30** | .45** | -.37** | -.19** | -.37** | -.16* | -.23** |
| Affective instability | .88 | -.50** | **.71**** | **-.51**** | -.24** | **-.45**** | -.27** | -.26** |
| Emptiness | .81 | -.46** | .58** | **-.51**** | -.31** | -.42** | -.26** | -.22** |
| Anger | .78 | -.42** | .39** | -.32 | -.08 | -.29** | -.17** | -.20** |
| Paranoid ideation | .84 | -.37** | .44** | -.39** | -.11 | -.36** | -.26** | -.28** |
| Total score | **.90** | **-.50**** | **.59**** | **-.45**** | **-.19**** | **-.42**** | **-.31**** | **-.30**** |

*Note.* ** Correlation is significant at the 0.01 level (two-tailed). * Correlation is significant at the 0.05 level (two-tailed). Correlations without a star are not significant. Important findings are highlighted (bold and underlined). SCL= Symptom Checklist-90, WHO= World Health Organization, f.=feelings. Only the total score of the SCL was analyzed, as data on the subscales are missing.

**Table 12**

*Known-groups validity of the Dutch BPDCL*

| Kruskal-Wallis post hoc tests | | | |
| --- | --- | --- | --- |
| Subscale | Sample I | Sample J | Adjusted significance |
| Abandonment | BPD | Other PD | .001 |
|  | BPD | Axis I | .000 |
|  | BPD | Healthy control | .000 |
|  | Other PD | Axis I | .126 |
|  | Other PD | Healthy control | .000 |
|  | Axis I | Other PD | .000 |
|  | Axis I | Healthy control | .000 |
| Interpersonal relationships | BPD | Other PD | .000 |
|  | BPD | Axis I | .000 |
|  | BPD | Healthy control | .000 |
|  | Other PD | Axis I | .759 |
|  | Other PD | Healthy control | .000 |
|  | Axis I | Other PD | .000 |
|  | Axis I | Healthy control | .000 |
| Identity disturbance | BPD | Other PD | .002 |
|  | BPD | Axis I | .000 |
|  | BPD | Healthy control | .000 |
|  | Other PD | Axis I | .017 |
|  | Other PD | Healthy control | .000 |
|  | Axis I | Other PD | .000 |
|  | Axis I | Healthy control | .193 |
| Impulsivity | BPD | Other PD | .000 |
|  | BPD | Axis I | .000 |
|  | BPD | Healthy control | .000 |
|  | Other PD | Axis I | .000 |
|  | Other PD | Healthy control | .000 |
|  | Axis I | Other PD | .872 |
|  | Axis I | Healthy control | .104 |
| Parasuicidal behavior | BPD | Other PD | .007 |
|  | BPD | Axis I | .000 |
|  | BPD | Healthy control | .000 |
|  | Other PD | Axis I | .000 |
|  | Other PD | Healthy control | .000 |
|  | Axis I | Other PD | .174 |
|  | Axis I | Healthy control | .448 |
| Affective instability | BPD | Other PD | .000 |
|  | BPD | Axis I | .000 |
|  | BPD | Healthy control | .000 |
|  | Other PD | Axis I | .767 |
|  | Other PD | Healthy control | .000 |
|  | Axis I | Other PD | .000 |
|  | Axis I | Healthy control | .000 |
| Emptiness | BPD | Other PD | .001 |
|  | BPD | Axis I | .000 |
|  | BPD | Healthy control | .000 |
|  | Other PD | Axis I | .058 |
|  | Other PD | Healthy control | .000 |
|  | Axis I | Other PD | .000 |
|  | Axis I | Healthy control | .000 |
| Anger | BPD | Other PD | .000 |
|  | BPD | Axis I | .000 |
|  | BPD | Healthy control | .000 |
|  | Other PD | Axis I | .452 |
|  | Other PD | Healthy control | .000 |
|  | Axis I | Other PD | .000 |
|  | Axis I | Healthy control | .015 |
| Paranoid and dissociation symptoms | BPD | Other PD | .000 |
|  | BPD | Axis I | .000 |
|  | BPD | Healthy control | .000 |
|  | Other PD | Axis I | .440 |
|  | Other PD | Healthy control | .000 |
|  | Axis I | Other PD | .000 |
|  | Axis I | Healthy control | .000 |
| Total score | BPD | Other PD | .000 |
|  | BPD | Axis I | .000 |
|  | BPD | Healthy control | .000 |
|  | Other PD | Axis I | .148 |
|  | Other PD | Healthy control | .000 |
|  | Axis I | Other PD | .000 |
|  | Axis I | Healthy control | .000 |

*Note.* BPD= Borderline Personality Disorder, other PD= non-BPD Personality disorder. The adjusted significance after Bonferroni correction is displayed.

### ***Conclusion***

To sum up, the Dutch BPDCL seems to possess good to excellent psychometric qualities. The Cronbach’s Alpha of the total scale was .96. The reliability coefficients of the subscales ranged from .65 (*Impulsivity*) to .90 (*Affective instability*). The Dutch BPDCL demonstrated high convergent validity with other mental health measures, such as the BSI-53 and the BPDSI. Finally, the BPDCL total score seems to discriminate well between BPD symptomatic, other psychopathology and healthy psychological traits.
