# Supplementary material for: Psychometric Evaluation of the Borderline Personality Disorder Checklist
Source: Int J Methods Psychiatr Res. 2025 Sep 25;34(3):e70029. doi: 10.1002/mpr.70029 (PMC12461754; doi:10.1002/mpr.70029)
Supplement: Supplementary file 1 — Supporting Information S1 [file MPR-34-e70029-s001.zip › Wiley_SM3- Spanish .docx]

**Supplementary material for the Spanish dataset**

**eAppendix 1.** Descriptives of the Spanish sample

**eTable 1.** Demographics

**eTable 2.** Clinical information

**eAppendix 2.** Item analyses of the Spanish BPDCL

**eTable 3.** Results of Shapiro Wilk test

**eTable 4**. Results of individual item analyses

**eAppendix 3.** Reliability analyses of the Spanish BPDCL

**eTable 5.** Reliability coefficients of each BPDCL subscales (total sample)

**eTable 6.** Reliability coefficients of each BPDCL subscales (BPD sample)

**eAppendix 4**. Convergent validity of the Spanish BPDCL

**eTable 7.** BPDCL and number BPD criteria (SCID-II)

**eAppendix 5**. Known-groups validity of Spanish BPDCL

**eTable 8.** Results of Mann Whitney U test

This supplementary material has been provided by the authors to give readers additional information about their work.

**eAppendix 1.** Descriptives of the Spanish sample

The Spanish sample consists of 311 BPD patients and 104 other PD patients. Demographics for the two groups can be seen in Table 1. The age of the total sample ranged from 18 to 69, with a mean age of 30.13 years (SD=8.36). The majority identified as female, were currently single and were currently unemployed. All participants came from Western Europe and were treated in an outpatient setting. The two clinical samples differed with respect to job status (χ² (4)= 10.24, p=.037) and gender (χ² (1)=15.46, p<.001). There was no difference across the groups with respect to age (t(413)= -1.47, p=.143) and marital status (χ² (3)=2.37 p=.500).

**eTable 1**

*Sociodemographic data of the Spanish sample (N=415)*

|  | BPD  N=311 | Other PD  N=104 |
| --- | --- | --- |
|  |  |  |
|  | n (%) | n (%) |
| Gender |  |  |
| Female | 262 (84.24) | 69 (66.35) |
| Male | 49 (15.76) | 35 (33.65) |
| Marital status |  |  |
| Single | 207 (66.77) | 76 (74.51) |
| Married or lasting relationship | 60 (19.35) | 15 (14.71) |
| Separated divorced | 42 (13.55) | 11 (10.78) |
| Widowed | 1 (0.32) | 0 (0) |
| Ethnicity |  |  |
| Western Europe | 311 (100) | 104 (100) |
| Employment |  |  |
| Student | 51 (17.29) | 9 (9.00) |
| Sickness benefits | 17 (5.76) | 8 (8.00) |
| Working | 78 (26.44) | 37 (37.00) |
| Unemployed | 149 (50.51) | 45 (45.00) |
| Other | 0 (0) | 1 (1.00) |
|  | Mean (SD) | Mean (SD) |
| Age | 29.78 (8.25) | 31.17 (8.65) |

*Note.* n= frequency, SD= Standard deviation. Only valid percentages

are reported.

**eTable 2.**

*Clinical information on the Spanish sample*

|  | BPD  N=311 | Other PD  N=104 |
| --- | --- | --- |
|  |  |  |
|  | n (%) | n (%) |
| Axis I disorder |  |  |
| Affective | 137(45.51) | 40 (40.00) |
| Anxiety | 194 (64.24) | 53 (53.00) |
| Substance use | 163 (53.97) | 39 (39.00) |
| Eating | 54 (17.94) | 12 (12.00) |
| Other | 26 (8.61) | 18 (18.00) |
| Axis II disorder |  |  |
| Avoidant | 112 (36.36) | 27 (25.96) |
| Dependent | 58 (18.83) | 11 (10.58) |
| OCPD | 89 (28.90) | 40 (38.46) |
| Paranoid | 115 (37.46) | 21 (20.19) |
| Schizotyp | 9 (2.93) | 3 (2.88) |
| Schizoid | 9 (2.93) | 5 (4.81) |
| Histrionic | 42 (13.64) | 13 (12.50) |
| Borderline | 311 (100) | 0 (0) |
| Narcissistic | 30 (9.74) | 11 (10.58) |
| Antisocial | 61 (19.81) | 23 (22.12) |
| Unspecified PD | 151 (49.19) | 44 (42.21) |

*Note.* All participants were outpatients. n= Frequency, Other PD

= other than BPD, PD= Personality disorder, OCPD= Obsessive

Compulsive Personality Disorder. Only valid percentages are

reported.

**eAppendix 2.** Item analyses of the Spanish BPDCL

According to the Shapiro-Wilk’s test and the visual inspection of the data, the assumption of normality of the data is not met (p<.001, see Table 3). The results of the item analysis are presented in Table 4. Item means ranged from 1.19 (item 8) to 4.16 (item 36) for the total sample. All item responses, ranging from 1 to 5, have been selected. The mean inter item correlation was 0.25, which is within the predefined range of 0.20 to 0.40. Item 8, 12, 17 and 35 have corrected item total correlations below 0.30. Cronbach’s Alpha if those items were deleted does not differ from the initial Cronbach’s Alpha of the total scale (Cronbach’s Alpha=0.94). The total scale score ranged from 55 to 212, with a mean of 124.37 (SD= 33.83).

For the Spanish sample, reliability coefficients of each subscale were higher than the predefined value of 0.70 for, except for the *Interpersonal relationship* (Cronbach’s Alpha= 0.65), *Impulsivity* (Cronbach’s Alpha= 0.63) and *Anger* (Cronbach’s Alpha= 0.69) scales. Guttman’s Lamda2 were slightly higher than Cronbach’s Alpha. McDonal’s Omega should be looked at with caution, as the normality of the data was not met. Excluding items with a corrected item total correlation below .30 did not change the reliability of those scales in a positive way. We also examined the reliability coefficients for the BPD only sample (N=311). Reliability coefficients were below .70 for the following subscales: *Interpersonal relationship*s (.66*), Impulsivity* (.62) and *Anger* (.66). The Cronbach’s Alpha of the BPDCL total scale was .93.

**eTable 3.**

*Test of normality of the Spanish BPDCL*

| Shapiro-Wilk | | | |
| --- | --- | --- | --- |
|  | Statistic | df | Sig. |
| item 1 | .84 | 412 | <.001 |
| item 2 | .80 | 412 | <.001 |
| item 3 | .88 | 412 | <.001 |
| item 4 | .81 | 412 | <.001 |
| item 5 | .62 | 412 | <.001 |
| item 6 | .73 | 412 | <.001 |
| item 7 | .60 | 412 | <.001 |
| item 8 | .29 | 412 | <.001 |
| item 9 | .77 | 412 | <.001 |
| item 10 | .85 | 412 | <.001 |
| item 11 | .78 | 412 | <.001 |
| item 12 | .73 | 412 | <.001 |
| item 13 | .83 | 412 | <.001 |
| item 14 | .85 | 412 | <.001 |
| item 15 | .83 | 412 | <.001 |
| item 16 | .87 | 412 | <.001 |
| item 17 | .71 | 412 | <.001 |
| item 18 | .89 | 412 | <.001 |
| item 19 | .88 | 412 | <.001 |
| item 20 | .80 | 412 | <.001 |
| item 21 | .84 | 412 | <.001 |
| item 22 | .58 | 412 | <.001 |
| item 23 | .60 | 412 | <.001 |
| item 24 | .72 | 412 | <.001 |
| item 25 | .81 | 412 | <.001 |
| item 26 | .65 | 412 | <.001 |
| item 27 | .82 | 412 | <.001 |
| item 28 | .67 | 412 | <.001 |
| item 29 | .83 | 412 | <.001 |
| item 30 | .85 | 412 | <.001 |
| item 31 | .65 | 412 | <.001 |
| item 32 | .85 | 412 | <.001 |
| item 33 | .87 | 412 | <.001 |
| item 34 | .85 | 412 | <.001 |
| item 35 | .45 | 412 | <.001 |
| item 36 | .72 | 412 | <.001 |
| item 37 | .77 | 412 | <.001 |
| item 38 | .86 | 412 | <.001 |
| item 39 | .88 | 412 | <.001 |
| item 40 | .82 | 412 | <.001 |
| item 41 | .60 | 412 | <.001 |
| item 42 | .86 | 412 | <.001 |
| item 43 | .85 | 412 | <.001 |
| item 44 | .82 | 412 | <.001 |
| item 45 | .82 | 412 | <.001 |
| item 46 | .87 | 412 | <.001 |
| item 47 | .66 | 412 | <.001 |

*Note*. df= degress of freedom, Sig.=Significance. Cases were excluded

listwise.

**eTable 4.**

*Item analysis of the Spanish BPDCL*

|  | Mean | SD | rtot | α _if item was deleted_ | |
| --- | --- | --- | --- | --- | --- |
| item 1 | 2.59 | 1.50 | .38 | | .94 |
| item 2 | 4.10 | 1.04 | .53 | | .94 |
| item 3 | 3.25 | 1.35 | .53 | | .94 |
| item 4 | 2.46 | 1.53 | .48 | | .94 |
| item 5 | 1.62 | 1.10 | .33 | | .94 |
| item 6 | 2.04 | 1.40 | .47 | | .94 |
| item 7 | 1.68 | 1.22 | .31 | | .94 |
| item 8 | 1.19 | .70 | .22 | | .94 |
| item 9 | 2.32 | 1.55 | .53 | | .94 |
| item 10 | 2.97 | 1.55 | .59 | | .94 |
| item 11 | 3.99 | 1.25 | .61 | | .94 |
| item 12 | 1.98 | 1.35 | .25 | | .94 |
| item 13 | 3.49 | 1.48 | .57 | | .94 |
| item 14 | 2.70 | 1.52 | .68 | | .94 |
| item 15 | 3.77 | 1.27 | .55 | | .94 |
| item 16 | 2.83 | 1.46 | .55 | | .94 |
| item 17 | 1.83 | 1.22 | .09 | | .94 |
| item 18 | 3.17 | 1.34 | .59 | | .94 |
| item 19 | 3.29 | 1.39 | .48 | | .94 |
| item 20 | 2.18 | 1.35 | .39 | | .94 |
| item 21 | 2.92 | 1.57 | .53 | | .94 |
| item 22 | 1.61 | 1.18 | .32 | | .94 |
| item 23 | 1.61 | 1.13 | .43 | | .94 |
| item 24 | 1.96 | 1.34 | .48 | | .94 |
| item 25 | 3.85 | 1.30 | .65 | | .94 |
| item 26 | 1.88 | 1.40 | .43 | | .94 |
| item 27 | 3.07 | 1.65 | .55 | | .94 |
| item 28 | 1.95 | 1.45 | .53 | | .94 |
| item 29 | 2.76 | 1.59 | .36 | | .94 |
| item 30 | 2.83 | 1.53 | .60 | | .94 |
| item 31 | 1.80 | 1.29 | .54 | | .94 |
| item 32 | 2.59 | 1.47 | .48 | | .94 |
| item 33 | 3.06 | 1.49 | .57 | | .94 |
| item 34 | 3.11 | 1.52 | .58 | | .94 |
| item 35 | 1.32 | .82 | .09 | | .94 |
| item 36 | 4.16 | 1.19 | .54 | | .94 |
| item 37 | 2.29 | 1.52 | .45 | | .94 |
| item 38 | 2.96 | 1.53 | .48 | | .94 |
| item 39 | 2.97 | 1.43 | .67 | | .94 |
| item 40 | 3.43 | 1.55 | .49 | | .94 |
| item 41 | 1.73 | 1.32 | .35 | | .94 |
| item 42 | 2.76 | 1.50 | .62 | | .94 |
| item 43 | 3.44 | 1.44 | .56 | | .94 |
| item 44 | 3.51 | 1.49 | .67 | | .94 |
| item 45 | 2.65 | 1.59 | .59 | | .94 |
| item 46 | 2.92 | 1.50 | .53 | | .94 |
| item 47 | 1.76 | 1.23 | .50 | | .94 |

*Note.* SD= Standard deviation, r_tot=_ corrected item total correlation,

α= Cronbach’s Alpha.

**eAppendix 3.** Reliability analyses of the Spanish BPDCL

**eTable 5.**

*Reliability coefficients of each BPDCL subscale for the Spanish sample (N=415)*

|  | Cronbach’s Alpha | Guttman Lamda2 | McDonald’sOmega |
| --- | --- | --- | --- |
| Fear of Abandonment | .80 | .81 | .80 |
| Interpersonal relationships | .65 | .66 | .67 |
| Identity disturbance | .80 | .81 | .81 |
| Impulsivity | .63 | .65 | .62 |
| Parasuicidal behaviour | .81 | .82 | .82 |
| Affective instability | .77 | .77 | .77 |
| Emptiness | - | - | - |
| Anger | .69 | .70 | .70 |
| Paranoid and dissociative behavior | .80 | .80 | .80 |
| Total scale | .94 | .94 | .94 |

*Note.* McDonal’s Omega should be interpreted with caution, as the assumption of normality was violated.

**eTable 6.**

*Reliability coefficients of each BPDCL subscale for the Spanish BPD sample (N=311)*

|  | Cronbach’s Alpha | Guttman Lamda2 | McDonald’sOmega |
| --- | --- | --- | --- |
| Fear of Abandonment | .79 | .80 | .79 |
| Interpersonal relationships | .66 | .67 | .68 |
| Identity disturbance | .79 | .80 | .79 |
| Impulsivity | .62 | .64 | .61 |
| Parasuicidal behaviour | .81 | .81 | .82 |
| Affective instability | .72 | .72 | .72 |
| Emptiness | - | - | - |
| Anger | .66 | .68 | .67 |
| Paranoid and dissociative behavior | .78 | .79 | .78 |
| Total scale | .93 | .94 | .93 |

*Note.* McDonal’s Omega should be interpreted with caution, as the assumption of normality was violated.

**eAppendix 4**. Convergent validity of the Spanish BPDCL

We did run a correlation analysis on the number of BPD criteria and BPDCL scale scores. The number of BPD criteria did not correlate in an acceptable manner with any BPDCL subscore (<.45, see Table 9). The number of BPD criteria correlated weakly with the BPDCL total score (.39).

**eTable 7.**

*Spearman’s Rho correlations of the Spanish*

*BPDCL and the number of BPD criteria*

|  | # BPD criteria |
| --- | --- |
| Fear of Abandonment | .35 |
| Interpersonal relationships | .32 |
| Identity disturbance | .34 |
| Impulsivity | .23 |
| Parasuicidal behaviour | .27 |
| Affective instability | .26 |
| Emptiness | .32 |
| Anger | .34 |
| Paranoid and dissociative behaviors | .34 |
| Total scale | .39 |

*Note.* # = Number

**eAppendix 5**. Known-groups validity of Spanish BPDCL

We did investigate whether the BPDCL total score distinguishes well between the BPD and the Other PD sample. The effect size was calculated by dividing the standardized test statistic (z-value) by the square root of the total number of cases (Pallant, 2016). Effect sizes can be interpreted as small (r=.10), medium (r=.30) and large (r=.50). The Mann Whitney U test for the BPDCL total score was significant (U=9832.50, z=-5.811, p<.001, r=0.29). Looking at the medians (Md) of the samples, BPD patients (Md=129, n=309) scored higher on the BPDCl total score compared to other PD patients (Md= 105, n=103). The total score of the BPDCL obtained an effect size of .29, indicating an approximately medium effect size (r=.30; Pallant, 2016). The Mann Whitney U test was significant for each subscale of the BPDCL (p<.001). Effect sizes ranged from small to medium (see Table 10).

**eTable 8.**

*Known-groups validity of the Spanish BPDCL*

|  |  | | Mann Whitney U test | | | | |  |
| --- | --- | --- | --- | --- | --- | --- | --- | --- |
| Scale | | (I) | | (J) | Sig. | n | Effect size | |
| Total score | | BPD | | Other PD | <.001 | 412 | .29 | |
| Abandonment | | BPD | | Other PD | <.001 | 414 | .23 | |
| Interpersonal relationships | | BPD | | Other PD | <.001 | 414 | .23 | |
| Identity | | BPD | | Other PD | <.001 | 413 | .22 | |
| Impulsivity | | BPD | | Other PD | <.001 | 414 | .17 | |
| Parasuicidal behavior | | BPD | | Other PD | <.001 | 414 | .22 | |
| Affective instability | | BPD | | Other PD | <.001 | 414 | .21 | |
| Emptiness | | BPD | | Other PD | <.001 | 415 | .25 | |
| Anger | | BPD | | Other PD | <.001 | 414 | .26 | |
| Paranoid and dissociative ideation | | BPD | | Other PD | <.001 | 413 | .26 | |

*Note.* BPD= Borderline Personality Disorder, PD=Personality disorder, HC= Healthy controls. Sig.= Significance, n=total number of cases.

### ***Conclusion***

To sum up, a total of 415 participants filled out the Spanish version of the BPDCL. The sample consisted of 311 BPD and 104 other PD patients. The Spanish version of the BPDCL seems to possess good psychometric qualities. The reliability coefficient of the Spanish total score was .94. The reliability coefficients of the individual subscales ranged from .63 (*Impulsivity*) to .81 (*Parasuicidal behaviors*). Convergent validity with the number of BPD criteria (SCID-II) was weak (<.45). The Spanish BPDCL seems to discriminate well between BPD and other PD patients.
