# Supplementary material for: Psychometric Evaluation of the Borderline Personality Disorder Checklist
Source: Int J Methods Psychiatr Res. 2025 Sep 25;34(3):e70029. doi: 10.1002/mpr.70029 (PMC12461754; doi:10.1002/mpr.70029)
Supplement: Supplementary file 1 — Supporting Information S1 [file MPR-34-e70029-s001.zip › Wiley_SM4- ItalianSample.docx]

**Supplementary material for the Italian dataset**

**eAppendix 1.** Descriptives of the Italian sample

**eTable 1.** Demographics

**eTable 2.** Clinical information

**eAppendix 2.** Item analyses of the Italian BPDCL

**eTable 3.** Results of Shapiro Wilk test

**eTable 4**. Results of individual item analyses

**eAppendix 3.** Reliability analyses of the Italian BPDCL

**eTable 5.** Reliability coefficients of each BPDCL subscales (total sample)

**eTable 6.** Reliability coefficients of each BPDCL subscales (BPD sample)

**eAppendix 4**. Convergent validity of the Italian BPDCL

**eTable 7.** Means and standard deviations of each instrument

**eTable 8.** BPDCL and SCL-90

**eTable 9.** BPDCL and IPO

**eAppendix 5**. Known-groups validity of Italian BPDCL

**eTable 10.** Results of Kruskal-Wallis test

This supplementary material has been provided by the authors to give readers additional information about their work.

**eAppendix 1.** Descriptives of the Italian sample

A total of 1795 participants filled out the Italian version of the BPDCL. The Italian sample consisted of 30 BPD patients, 66 clinical controls (other personality disorder) and 1699 healthy controls. See Table 1 for a detailed overview. Age ranged from 18 to 66 with a mean age of 23.44 (SD=5.67). Mean age differences across BPD patients, clinical controls and healthy controls were inspected.  Levene's test assumes that the assumption of equal variances should be rejected (F(2, 1787)= 143.66, p<.001). The Welch ANOVA indicates that the mean age differs across the three groups (F(2, 53.84)=97.55, p<.001). Games-Howell post hoc tests were applied (Games & Howell, 1976). The two clinical groups did not differ with respect to age (p=.53). Healthy controls were significantly younger than the two clinical samples (p<.001). The majority of the Italian sample identified as female, came from Western Europe and was currently studying at a university. The three subsamples differed significantly with respect to gender (X2 (2, N = 1795) = 23.46, p < .001) and job status (X2 (6, N=1789) = 1522.54, p < .001). Marital status was only assessed in the two clinical groups (BPD and other PD) and nationality was only assessed for the healthy controls. Looking at the BPD sample exclusively, the majority were currently employed and were single. For the two clinical groups comorbidities with other mental health disorders were high, see Table 2

**eTable 1.**

*Sociodemographic data of the Italian sample*

|  | BPD  N=30 | Other PD  N=66 | HC  N=1699 |
| --- | --- | --- | --- |
|  | n (%) | n (%) | n (%) |
| Gender |  |  |  |
| Female | 22 (73.33) | 36 (54.55) | 1348 (79.34) |
| Male | 8 (26.67) | 30 (45.45) | 351 (20.66) |
| Marital status |  |  |  |
| Single | 19 (63.33) | 38 (58.46) | n.a. |
| Married or lasting relationship | 5 (16.67) | 18 (27.69) | n.a. |
| Separated or divorced | 6 (20.00) | 9 (13.85) | n.a. |
| Nationality |  |  |  |
| Western Europe | n.a. | n.a. | 1633 (96.51) |
| Eastern Europe | n.a. | n.a. | 30 (1.77) |
| South America | n.a. | n.a. | 19 (1.12) |
| Africa | n.a. | n.a. | 6 (0.35) |
| Asia | n.a. | n.a. | 4 (0.24) |
| Employment |  |  |  |
| Student | 5 (1724) | 8 (13.11) | 1699 (100) |
| Working | 14 (48.28) | 32 (52.46) | 0 (0.00) |
| Unemployed | 9 (31.03) | 18 (29.51) | 0 (0.00) |
| Retired | 1 (3.45) | 3 (4.92) | 0 (0.00) |
|  | Mean (SD) | Mean (SD) | Mean (SD) |
| Age | 36.13 (9.99) | 38.58 (10.79) | 22.62 (3.91) |

*Note.* n.a.= not assessed. Nationality for the two clinical samples as well as marital status for the healthy sample were not assessed. Only valid percentages are reported.

**eTable 2.**

*Clinical information of the Italian clinical samples*

|  | BPD  N=30 | Other PD  N=66 |
| --- | --- | --- |
|  | n(%) | n(%) |
| Setting |  |  |
| Inpatient | 18 (60.00) | 37 (56.06) |
| Outpatient | 12 (40.00) | 29 (43.94) |
| Axis I disorder |  |  |
| Affective | 11 (37.93) | 22 (34.38) |
| Anxiety | 0 (0.00) | 8 (12.70) |
| Substance use | 9 (31.03) | 25 (39.06) |
| Eating | 7 (24.14) | 7 (11.11) |
| Other | 1 (3.45) | 2 (3.17) |
| Axis II disorder |  |  |
| Avoidant PD | 4 (13.33) | 5 (7.58) |
| Dependent PD | 2 (6.67) | 8 (12.12) |
| OCPD | 2 (6.67) | 6 (9.09) |
| Paranoid PD | 2 (6.67) | 5 (7.58) |
| Schizotyp PD | 0 (0.00) | 3 (4.55) |
| Schizoid PD | 0 (0.00) | 1 (1.52) |
| Histrionic PD | 4 (13.33) | 6 (9.09) |
| Borderline PD | 30 (100) | 0 (0.00) |
| Narcissistic PD | 6 (20.00) | 6 (9.09) |
| Antisocial PD | 3 (10.00) | 7 (10.61) |
| Unspecified PD | 4 (13.33) | 39 (59.09) |

Note. n=Frequency. PD= Personality disorder. OCPD=

Obsessive-compulsive personality disorder. Only

valid percentages are reported.

**eAppendix 2.** Item analyses of the Italian BPDCL

According to the Shapiro-Wilk’s test and the visual inspection of the data, the assumption of normality of the data is not met (p<.001). Item means range from 1.10 (item 35) to 3.05 (item 2) for the Italian sample. Some items were centered at one end of the response scale (e.g. Item 47 with a mean of 1.20). The mean inter item correlation is 0.29, which is within the predefined range of 0.20 to 0.40. There are no negative inter-item correlations, which indicates that the scale is measuring a one -dimensional construct. The variance of the mean-inter-item correlation (s²=.01) is rather small. Thus, the individual inter-item correlations do not vary strongly. Item 8 and 17 have corrected item total correlations below 0.30. Cronbach’s Alpha if those items were deleted do not differ from the initial Cronbach’s Alpha of the total scale (Cronbach’s Alpha=0.95).  Thus, this small deviation from .3 can be ignored, as the scale reliability is not affected by those items. The total scale mean was 83.79 (SD=25.21). The BPDCL means are rather small, as the sample consists of mainly healthy controls.  The highest score was 235 and the lowest was 47. Thus, both extremes have been scored. Also, all item responses from 1 to 5 were scored for each item.

Reliability coefficients for the Italian sample can be seen in Table 5. Reliability coefficients of each subscale were higher than the predefined value of 0.70, except for the *Anger* subscale (Cronbach’s Alpha= 0.67). Cronbach’s Alpha, Guttman’s Lamda2 and McDonal’s Omega did not differ greatly from one another. The reliability coefficients for the BPD only sample can be seen in Table 6. The coefficients were within the predefined range for the following subscales only: BPDCL total score, *identity disturbance, affective instability* and *paranoid and dissociative symptoms*. The Cronbach’s Alpha of the total scale was 0.91, thus slightly lower than for the total sample. However, those results might be biased, as the BPD sample consists only of 30 participants.

**eTable 3.**

*Test of normality of the Italian BPDCL*

| Shapiro-Wilk | | | |
| --- | --- | --- | --- |
|  | Statistic | df | Sig. |
| item 1 | .83 | 1782 | <.001 |
| item 2 | .92 | 1782 | <.001 |
| item 3 | .88 | 1782 | <.001 |
| item 4 | .80 | 1782 | <.001 |
| item 5 | .39 | 1782 | <.001 |
| item 6 | .28 | 1782 | <.001 |
| item 7 | .47 | 1782 | <.001 |
| item 8 | .34 | 1782 | <.001 |
| item 9 | .37 | 1782 | <.001 |
| item 10 | .85 | 1782 | <.001 |
| item 11 | .90 | 1782 | <.001 |
| item 12 | .68 | 1782 | <.001 |
| item 13 | .89 | 1782 | <.001 |
| item 14 | .78 | 1782 | <.001 |
| item 15 | .92 | 1782 | <.001 |
| item 16 | .71 | 1782 | <.001 |
| item 17 | .38 | 1782 | <.001 |
| item 18 | .83 | 1782 | <.001 |
| item 19 | .90 | 1782 | <.001 |
| item 20 | .82 | 1782 | <.001 |
| item 21 | .76 | 1782 | <.001 |
| item 22 | .66 | 1782 | <.001 |
| item 23 | .63 | 1782 | <.001 |
| item 24 | .48 | 1782 | <.001 |
| item 25 | .79 | 1782 | <.001 |
| item 26 | .20 | 1782 | <.001 |
| item 27 | .34 | 1782 | <.001 |
| item 28 | .22 | 1782 | <.001 |
| item 29 | .73 | 1782 | <.001 |
| item 30 | .70 | 1782 | <.001 |
| item 31 | .41 | 1782 | <.001 |
| item 32 | .81 | 1782 | <.001 |
| item 33 | .69 | 1782 | <.001 |
| item 34 | .86 | 1782 | <.001 |
| item 35 | .22 | 1782 | <.001 |
| item 36 | .88 | 1782 | <.001 |
| item 37 | .51 | 1782 | <.001 |
| item 38 | .77 | 1782 | <.001 |
| item 39 | .86 | 1782 | <.001 |
| item 40 | .87 | 1782 | <.001 |
| item 41 | .51 | 1782 | <.001 |
| item 42 | .82 | 1782 | <.001 |
| item 43 | .81 | 1782 | <.001 |
| item 44 | .69 | 1782 | <.001 |
| item 45 | .60 | 1782 | <.001 |
| item 46 | .79 | 1782 | <.001 |
| item 47 | .35 | 1782 | <.001 |

*Note*. df= degress of freedom, Sig.=Significance. Cases were

excluded listwise.

**eTable 4.**

Item analysis of the Italian BPDCL

|  | Mean | SD | r_tot_ | α _if item was deleted_ |
| --- | --- | --- | --- | --- |
| item 1 | 2.05 | 1.11 | .35 | .95 |
| item 2 | 3.05 | 1.16 | .53 | .95 |
| item 3 | 2.32 | 1.10 | .46 | .95 |
| item 4 | 2.03 | 1.20 | .55 | .95 |
| item 5 | 1.21 | 0.60 | .43 | .95 |
| item 6 | 1.15 | 0.58 | .43 | .95 |
| item 7 | 1.34 | 0.82 | .43 | .95 |
| item 8 | 1.18 | 0.60 | .29 | .95 |
| item 9 | 1.24 | 0.72 | .55 | .95 |
| item 10 | 2.17 | 1.16 | .67 | .95 |
| item 11 | 2.55 | 1.18 | .65 | .95 |
| item 12 | 1.63 | 0.98 | .34 | .95 |
| item 13 | 2.52 | 1.26 | .58 | .95 |
| item 14 | 1.91 | 1.11 | .65 | .95 |
| item 15 | 2.95 | 1.21 | .60 | .95 |
| item 16 | 1.63 | 0.91 | .62 | .95 |
| item 17 | 1.23 | 0.68 | .28 | .95 |
| item 18 | 2.08 | 1.14 | .62 | .95 |
| item 19 | 2.51 | 1.03 | .52 | .95 |
| item 20 | 1.92 | 0.91 | .34 | .95 |
| item 21 | 1.86 | 1.11 | .59 | .95 |
| item 22 | 1.56 | 0.91 | .36 | .95 |
| item 23 | 1.53 | 0.91 | .53 | .95 |
| item 24 | 1.31 | 0.73 | .54 | .95 |
| item 25 | 1.96 | 1.14 | .67 | .95 |
| item 26 | 1.11 | 0.54 | .44 | .95 |
| item 27 | 1.21 | 0.69 | .46 | .95 |
| item 28 | 1.12 | 0.54 | .48 | .95 |
| item 29 | 1.77 | 1.08 | .48 | .95 |
| item 30 | 1.65 | 0.95 | .66 | .95 |
| item 31 | 1.24 | 0.66 | .52 | .95 |
| item 32 | 2.03 | 1.17 | .57 | .95 |
| item 33 | 1.64 | 0.96 | .63 | .95 |
| item 34 | 2.23 | 1.17 | .66 | .95 |
| item 35 | 1.10 | 0.46 | .32 | .95 |
| item 36 | 2.47 | 1.34 | .68 | .95 |
| item 37 | 1.37 | 0.83 | .46 | .95 |
| item 38 | 1.82 | 1.01 | .48 | .95 |
| item 39 | 2.18 | 1.13 | .55 | .95 |
| item 40 | 2.47 | 1.34 | .53 | .95 |
| item 41 | 1.37 | 0.83 | .34 | .95 |
| item 42 | 2.03 | 1.12 | .63 | .95 |
| item 43 | 2.01 | 1.15 | .62 | .95 |
| item 44 | 1.67 | 1.01 | .68 | .95 |
| item 45 | 1.53 | 0.99 | .55 | .95 |
| item 46 | 1.92 | 1.11 | .63 | .95 |
| item 47 | 1.20 | 0.65 | .50 | .95 |

*Note.* SD= Standard deviation, r_tot_= corrected item-total correlation,

𝜶= Cronbach’s Alpha.

**eAppendix 3.** Reliability analyses of the Italian BPDCL

**eTable 5.**

*Reliability coefficients of each BPDCL subscale for the Italian sample (N=1795)*

|  | Cronbach’s Alpha | Guttman Lambda2 | McDonald’sOmega |
| --- | --- | --- | --- |
| Fear of Abandonment | .77 | .79 | .79 |
| Interpersonal relationships | .71 | .72 | .74 |
| Identity disturbance | .85 | .86 | .86 |
| Impulsivity | .73 | .73 | .73 |
| Parasuicidal behavior | .80 | .81 | .82 |
| Affective instability | .78 | .79 | .80 |
| Emptiness | - | - | - |
| Anger | .67 | .67 | .67 |
| Paranoid and dissociative behavior | .79 | .80 | .79 |
| Total scale | .95 | .95 | .95 |

*Note.* Reliability coefficients for the emptiness subscale could not be calculated, as it consists of only one item. The data is not normally distributed, that is why the McDonald’s Omega should be looked at with caution.

**eTable 6.**

*Reliability coefficients of each BPDCL subscale for the Italian BPD sample (N=30)*

|  | Cronbach’s Alpha | Guttman Lamda2 | McDonald’sOmega |
| --- | --- | --- | --- |
| Fear of Abandonment | .56 | .63 | - |
| Interpersonal relationships | .53 | .58 | .74 |
| Identity disturbance | .83 | .85 | .84 |
| Impulsivity | .48 | .55 | - |
| Parasuicidal behaviour | .57 | .60 | 0.86 |
| Affective instability | .70 | .70 | .69 |
| Emptiness | - | - | - |
| Anger | .38 | .41 | .44 |
| Paranoid and dissociative behavior | .72 | .74 | .69 |
| Total scale | .91 | .92 | - |

*Note.* Reliability coefficients for the emptiness subscale could not be calculated, as it consists of only one item.

### ***Convergent validity***

The means and standard deviation of each questionnaire are presented in Table 7. Convergent validity with other mental health questionnaires was examined. Due to the violation of normality, we reported Spearman’s Rho only. Correlations with other mental health outcomes were very strong (> .55).

The BPDCL total scale score correlated very strongly with the SCL sum score (.90). The strongest correlation with the SCL sum score demonstrated the *Affective instabi*lity scale (.86). The BPDCL also correlated strongly with the corresponding SCL subscales. SCL *insecurity* correlated especially strongly with the Paranoid BPDCL scale (.68). SCL *Depression* and *Anxiety* correlated the most with BPDCL *Affective instability* (.76 and .75, respectively). BPDCL *Anger* correlated with its corresponding SCL subscale *Hostility* (.71). The BPDCL *Paranoid and dissociative symptom* scale did correlate the most with its corresponding SCL scale *Paranoid symptoms* (.73).

The BPDCL subscales also showed strong correlations with the IPO subscales. BPDCL *identity disturbance* had the highest correlation with its corresponding IPO scale *Identity diffusion* (.65). BPDCL *Abandonment* had the strongest correlation with the *IPO Primitive psychological defenses scale* (.57). Finally, BPDCL *paranoid* scale had the strongest relationship with the IPO scale *Alternative reality testing* (.59).

**eTable 7.**

*Means and standard deviations of the questionnaires*

|  | N | Min | Max | Mean | SD |
| --- | --- | --- | --- | --- | --- |
| IPO Identity Diffusion | 96 | 1.10 | 4.38 | 2.53 | .65 |
| IPO Primitive Psychological Defenses | 96 | 1.44 | 4.31 | 2.57 | .62 |
| IPO Alternative Reality Testing | 96 | 1.00 | 4.40 | 2.02 | .64 |
| SCL Sum score | 52 | 103 | 351 | 199.42 | 69.89 |
| SCL  Somatization | 94 | 1.00 | 4.55 | 2.24 | .93 |
| SCL Obsession | 93 | 1.00 | 4.90 | 2.56 | .96 |
| SCL Insecurity | 94 | 1.00 | 4.89 | 2.28 | 1.01 |
| SCL Depression | 94 | 1.00 | 5.00 | 2.83 | 1.03 |
| SCL Anxiety | 94 | 1.00 | 5.00 | 2.35 | .95 |
| SCL Hostility | 94 | 1.00 | 4.50 | 1.78 | .74 |
| SCL Phobic anxiety | 94 | 1.00 | 4.43 | 1.79 | .87 |
| SCL Paranoid | 94 | 1.00 | 5.00 | 2.27 | .91 |
| SCL Psychoticism | 94 | 1.00 | 4.67 | 2.10 | .75 |

*Note.* SD= Standard deviation, N= Frequency, IPO= Inventory of Personality Organization, SCL = Symptom Checklist -90 revised. Only the BPD and the other PD group filled out the questionnaires. The means of the SCL scales are reported in this table.

|  | SCL Somatization | SCL Obsession | SCL  Insecurity | SCL  Depression | SCL  Anxiety | SCL  Hostility | SCL Phobia | SCL  Paranoid | SCL Psychotic | SCL  Total |
| --- | --- | --- | --- | --- | --- | --- | --- | --- | --- | --- |
| BPDCL subscales |  |  |  |  |  |  |  |  |  |  |
| Abandonment | .51** | .71** | **.66**** | .72** | **.70**** | .63** | .66** | .66** | .64** | **.76**** |
| Interpersonal relationships | .45** | .58** | .54** | .65** | .60** | .56** | .51** | .59** | .49** | **.68**** |
| Identity disturbance | .41** | .70** | **.66**** | .70** | .65** | .64** | .58** | .63** | .72** | **.77**** |
| Impulsivity | .27* | .44** | .26* | .36** | .42** | .37** | .27** | .28** | .32** | **.46**** |
| Parasuicidal behavior | .45** | .49** | .44** | .59** | .49** | .43** | .39** | .42** | .46** | **.65**** |
| Affective instability | .53** | .77** | .60** | **.76**** | **.75**** | .61** | .56** | .63** | .73** | **.86**** |
| Emptiness | .41** | .70** | .44** | .67** | .58** | .48** | .45** | .42** | .58** | **.74**** |
| Anger | .49** | .64** | **.66**** | .64** | .62** | .71** | .52** | .63** | .61** | **.81**** |
| Paranoid ideation | .47** | .66** | **.68**** | .63** | .63** | .59** | .56** | .73** | .63** | **.76**** |
| Total score | **.54**** | **.80**** | **.73**** | **.80**** | **.76**** | **.70**** | **.63**** | **.78**** | **.77**** | **.90**** |

**eTable 8.**

*Spearman's Rho correlations of the BPDCL and the SCL subscales for the Italian sample*

*Note.* ** Correlation is significant at the 0.01 level (two-tailed). * Correlation is significant at the 0.05 level (two-tailed). Correlations without a star are not significant. Important findings are highlighted (bold and underlined). SCL= Symptom Checklist-90-revised. The convergent validity could be calculated for the BPD and other PD sample only. 

**eTable 9.**

*Spearman's Rho correlations of the BPDCL and the IPO subscales for the Italian sample*

|  | IPO  Identity diffusion | IPO  Primitive defenses | IPO  Reality testing |
| --- | --- | --- | --- |
| BPDCL subscales |  |  |  |
| Abandonment | .61** | **.57**** | .47** |
| Interpersonal relationships | .40** | .43** | .43** |
| Identity disturbance | **.65**** | .46** | .57** |
| Impulsivity | .22* | .15 | .22* |
| Parasuicidal behavior | .27** | .29** | .28** |
| Affective instability | .61** | .53** | .52** |
| Emptiness | .50** | .34** | .46** |
| Anger | .55** | .50** | .45** |
| Paranoid ideation | .47** | .50** | **.59**** |
| Total score | **.64**** | **.56**** | **.57**** |

*Note.* IPO= Inventory of Personality Organization. ** Correlation is significant at the 0.01 level (two-tailed). * Correlation is significant at the 0.05 level (two-tailed). Correlations without a star are not significant. Important findings are highlighted (bold and underlined The convergent validity could be calculated for the BPD and other PD sample only.

**eAppendix 5**. Known-groups validity of Italian BPDCL

The Kruskal-Wallis test was significant, indicating that the BPD, other PD and healthy control groups differ significantly on the BPDCL total score (χ²(2, n=1782) =35.74, p<.001). Post hoc tests were significant (p<.05). Thus, all groups differed significantly from one another. When looking at the medians (Md), the BPD sample (Md=113, n=25) scored higher on the BPDCL total score compared to the other two groups. Participants of the other PD group (Md=89.50, n=58) scored higher than the healthy controls (Md= 78, n=1699). One can conclude that the Italian BPDCL total score distinguishes well between BPD patients, other PD patients and healthy controls. The individual subscales do not seem to differentiate well between the samples, as some post hoc comparisons are not significant (p>.05). Table 10 displays all BPDCL subscales post hoc tests.

**eTable 10.**

*Known-groups validity of the Italian BPDCL*

| Kruskal-Wallis post hoc tests | | | |
| --- | --- | --- | --- |
| Scale | Sample I | Sample J | Adj. significance |
| Total score | BPD | Other PD | .009 |
|  | BPD | HC | .000 |
|  | Other PD | HC | .017 |
| Abandonment | BPD | Other PD | .021 |
|  | BPD | HC | .000 |
|  | Other PD | HC | .004 |
| Interpersonal relationships | BPD | Other PD | .331 |
|  | BPD | HC | .015 |
|  | Other PD | HC | .554 |
| Identity | BPD | Other PD | .000 |
|  | BPD | HC | .000 |
|  | Other PD | HC | 1.000 |
| Impulsivity | BPD | Other PD | .000 |
|  | BPD | HC | .000 |
|  | Other PD | HC | .441 |
| Parasuicidal behavior | BPD | Other PD | .011 |
|  | BPD | HC | .000 |
|  | Other PD | HC | .000 |
| Affective instability | BPD | Other PD | .009 |
|  | BPD | HC | .000 |
|  | Other PD | HC | .000 |
| Emptiness | BPD | Other PD | .028 |
|  | BPD | HC | .000 |
|  | Other PD | HC | .000 |
| Anger | BPD | Other PD | .048 |
|  | BPD | HC | .000 |
|  | Other PD | HC | .534 |
| Paranoid and dissociative ideation | BPD | Other PD | .304 |
|  | BPD | HC | .003 |
|  | Other PD | HC | .165 |

*Note.* BPD= Borderline Personality Disorder, PD=Personality disorder, HC= Healthy controls. Adj.= Adjusted. The adjusted significance controls for multiple comparisons.

### ***Conclusion***

The Italian sample consisted of 1699 healthy controls, 30 BPD and 66 other PD patients. The Cronbach’s Alpha of the total scale was .95. Reliability coefficients of the subscales ranged from .67 (*Anger*) to .85 (*Identity disturbance*). Convergent validity with the SCL and the IPO subscales were excellent. The Italian BPDCL seems to discriminate well between BPD symptomatology, other clinical psychopathology, and healthy traits.

**References**

Games, P. A., & Howell, J. F. (1976). Pairwise multiple comparison procedures with unequal N’s and/or variances: A Monte Carlo study. Journal of Educational Statistics, 1(2), 113–125. <https://doi.org/10.3102/10769986001002113>
