# Supplementary material for: Psychometric Evaluation of the Borderline Personality Disorder Checklist
Source: Int J Methods Psychiatr Res. 2025 Sep 25;34(3):e70029. doi: 10.1002/mpr.70029 (PMC12461754; doi:10.1002/mpr.70029)
Supplement: Supplementary file 1 — Supporting Information S1 [file MPR-34-e70029-s001.zip › Wiley_SM5-German.docx]

**Supplementary material for the German dataset**

**eAppendix 1.** Descriptives of the German sample

**eTable 1.** Demographics

**eTable 2.** Clinical information

**eAppendix 2.** Item analyses of the German BPDCL

**eTable 3.** Results of Shapiro Wilk test

**eTable 4**. Results of individual item analyses

**eAppendix 3.** Reliability analyses of the German BPDCL

**eTable 5.** Reliability coefficients of each BPDCL subscales

**eAppendix 4**. Convergent validity of the German BPDCL

**eTable 6.** Means and standard deviations of each instrument

**eTable 7.** BPDCL and BPDSI

**eTable 8.** BPDCL and BSI-53

**eTable 9.** BPDCL and WHO-BREF

**eTable 10.** BPDCL and DBT-WCCL, WSAS and EuroQoL

This supplementary material has been provided by the authors to give readers additional information about their work.

**eAppendix 1.** Descriptives

The German sample consisted of 257 BPD patients and was drawn from the GST and ProBPD studies. Mean age was 32.05 (SD=10.40). The majority identified as female, was single, came from Western Europe and currently received sickness benefits. Comorbidities with other disorders were quite high, as 208 participants suffered from at least one anxiety disorder and 113 participants also met the SCID-II criteria for an avoidant personality disorder.

**eTable 1.**

*Demographics of the German sample*

|  | N=257 |
| --- | --- |
|  | n (%) |
| Gender |  |
| Female | 218 (85.16) |
| Male | 36 (14.06) |
| Other | 2 (0.78) |
| Marital status |  |
| Single | 122 (47.84) |
| Married or lasting relationship | 109 (42.75) |
| Separated | 19 (7.45) |
| Widowed | 1 (0.39) |
| Other | 4 (1.57) |
| Ethnicity |  |
| Western Europe | 240 (96.39) |
| Eastern Europe | 3 (1.20) |
| Africa | 1 (0.40) |
| Asia | 5 (2.01) |
| Employment |  |
| Homemaker | 15 (5.84) |
| Student | 14 (5.45) |
| Sickness benefits | 105 (40.86) |
| Working | 48 (18.68) |
| Unemployed | 21 (8.17) |
| Retired | 2 (0.78) |
| Other | 52 (20.23) |
|  | Mean (SD) |
| Age | 32.05 (10.40) |

*Note.* SD= Standard deviation, N=Frequency. Only

valid percentages are reported.

**eTable 2.**

*Clinical information of the German sample*

|  | N |
| --- | --- |
|  | n (%) |
| Axis I disorders |  |
| Affective | 200 (79.05) |
| Anxiety | 208 (92.04) |
| Substance use | 103 (49.28) |
| Eating | 131 (62.98) |
| Other | 151 (73.66) |
| Axis II disorders |  |
| Borderline PD | 257 (100) |
| Paranoid PD | 63 (75.39) |
| Schizoid PD | 7 (1.57) |
| Schizotypal | 4 (2.75) |
| Antisocial PD | 3 (1.34) |
| Narcissistic PD | 13 (5.10) |
| Histrionic PD | 12 (4.69) |
| Dependent PD | 38 (14.84) |
| Avoidant PD | 113 (43.97) |
| OCPD | 84 (32.94) |
| Unspecified PD | 9 (9.18) |

*Note.* N= Frequency, PD=Personality

Disorder, OC=Obsessive-compulsive. Only

valid percentages are reported.

**eAppendix 2.** Item analyses

According to the Shapiro-Wilk’s test and the visual inspection of the data, the assumption of normality of the data is not met (p<.001, see Table 3). The results of the item analyses are presented in Table 4. All answer options, ranging from 1 to 5, were chosen for all items. Item means ranged from 1.15 to 4.12 (mean=2.76). The results indicate that the responses of some items are centered at one end of the scale (e.g. Item 8 has a mean of 1.15). The mean inter-item-correlation of the total sample was .23, which is within the predefined range of .20 to .40. The Cronbach’s Alpha of the total scale was .93 (N=257). The corrected item total correlations lied between .01 (Item 8) and .63 (Item 25 and 47). All corrected item total correlations were above the predefined value of .30, except for the items 7, 8, 12,17 and 35. Cronbach’s Alpha if item was deleted did not change the overall Cronbach’s Alpha. Thus, there was no item that negatively affected the overall reliability of the scale. The total scale mean for the total sample was 129.72 (SD=27.79), with individual scores ranging from 66 to 208.

Cronbach’s Alpha, Guttman’s Lamda2 and McDonald Omega for each subscale can be seen in Table 5. Reliability coefficients were above the predefined score of .70 for each subscale of the BPDCL, except for Interpersonal relationships (.68), Impulsivity (.64) and Parasuicidal behaviors (.68). Cronbach’s Alpha ranged from .64 (Impulsivity) to .83 (Paranoid ideation). Guttman’s Lamda 2 was slightly higher than Cronbach’s Alpha, ranging from .66 (Impulsivity) to .84 (Paranoid ideation). Since the assumption of normality of the data was violated, McDonald’s Omega should only be cautiously looked at.

**eTable 3.**

*Test of normality of the German data*

| Shapiro-Wilk | | | |
| --- | --- | --- | --- |
|  | Statistic | df | Sig. |
| item 1 | .90 | 257 | <.001 |
| item 2 | .83 | 257 | <.001 |
| item 3 | .91 | 257 | <.001 |
| item 4 | .90 | 257 | <.001 |
| item 5 | .58 | 257 | <.001 |
| item 6 | .87 | 257 | <.001 |
| item 7 | .63 | 257 | <.001 |
| item 8 | .32 | 257 | <.001 |
| item 9 | .86 | 257 | <.001 |
| item 10 | .88 | 257 | <.001 |
| item 11 | .84 | 257 | <.001 |
| item 12 | .71 | 257 | <.001 |
| item 13 | .85 | 257 | <.001 |
| item 14 | .89 | 257 | <.001 |
| item 15 | .84 | 257 | <.001 |
| item 16 | .91 | 257 | <.001 |
| item 17 | .52 | 257 | <.001 |
| item 18 | .90 | 257 | <.001 |
| item 19 | .88 | 257 | <.001 |
| item 20 | .89 | 257 | <.001 |
| item 21 | .84 | 257 | <.001 |
| item 22 | .77 | 257 | <.001 |
| item 23 | .78 | 257 | <.001 |
| item 24 | .76 | 257 | <.001 |
| item 25 | .84 | 257 | <.001 |
| item 26 | .55 | 257 | <.001 |
| item 27 | .86 | 257 | <.001 |
| item 28 | .56 | 257 | <.001 |
| item 29 | .85 | 257 | <.001 |
| item 30 | .83 | 257 | <.001 |
| item 31 | .73 | 257 | <.001 |
| item 32 | .89 | 257 | <.001 |
| item 33 | .83 | 257 | <.001 |
| item 34 | .91 | 257 | <.001 |
| item 35 | .33 | 257 | <.001 |
| item 36 | .86 | 257 | <.001 |
| item 37 | .78 | 257 | <.001 |
| item 38 | .91 | 257 | <.001 |
| item 39 | .88 | 257 | <.001 |
| item 40 | .88 | 257 | <.001 |
| item 41 | .58 | 257 | <.001 |
| item 42 | .91 | 257 | <.001 |
| item 43 | .88 | 257 | <.001 |
| item 44 | .89 | 257 | <.001 |
| item 45 | .76 | 257 | <.001 |
| item 46 | .90 | 257 | <.001 |
| item 47 | .79 | 257 | <.001 |

*Note*. df= degress of freedom, Sig.=Significance.

Cases were excluded listwise.

**eTable 4.**

*Item analysis of the German BPDCL*

|  | Mean | SD | r_tot_ | α _if item was deleted_ |
| --- | --- | --- | --- | --- |
| item 1 | 2.65 | 1.26 | .35 | .93 |
| item 2 | 4.12 | 0.84 | .53 | .93 |
| item 3 | 3.08 | 1.28 | .40 | .93 |
| item 4 | 2.74 | 1.32 | .54 | .93 |
| item 5 | 1.47 | 0.92 | .31 | .93 |
| item 6 | 2.40 | 1.30 | .32 | .93 |
| item 7 | 1.65 | 1.14 | .21 | .93 |
| item 8 | 1.15 | 0.53 | .01 | .93 |
| item 9 | 2.37 | 1.27 | .48 | .93 |
| item 10 | 3.47 | 1.27 | .55 | .93 |
| item 11 | 3.96 | 1.03 | .57 | .93 |
| item 12 | 1.81 | 1.19 | .25 | .93 |
| item 13 | 3.63 | 1.34 | .37 | .93 |
| item 14 | 2.92 | 1.39 | .54 | .93 |
| item 15 | 3.95 | 1.07 | .54 | .93 |
| item 16 | 2.89 | 1.12 | .52 | .93 |
| item 17 | 1.44 | 0.96 | .10 | .93 |
| item 18 | 3.36 | 1.21 | .55 | .93 |
| item 19 | 3.68 | 1.11 | .49 | .93 |
| item 20 | 3.54 | 1.20 | .52 | .93 |
| item 21 | 3.78 | 1.22 | .55 | .93 |
| item 22 | 2.01 | 1.28 | .34 | .93 |
| item 23 | 2.09 | 1.31 | .46 | .93 |
| item 24 | 1.86 | 1.11 | .49 | .93 |
| item 25 | 3.96 | 1.03 | .63 | .93 |
| item 26 | 1.51 | 1.05 | .40 | .93 |
| item 27 | 2.58 | 1.44 | .59 | .93 |
| item 28 | 1.54 | 1.08 | .44 | .93 |
| item 29 | 2.71 | 1.51 | .31 | .93 |
| item 30 | 4.02 | 1.01 | .55 | .93 |
| item 31 | 1.84 | 1.18 | .54 | .93 |
| item 32 | 2.72 | 1.33 | .53 | .93 |
| item 33 | 3.99 | 1.06 | .54 | .93 |
| item 34 | 3.37 | 1.19 | .53 | .93 |
| item 35 | 1.17 | 0.58 | .16 | .93 |
| item 36 | 3.91 | 0.99 | .61 | .93 |
| item 37 | 2.10 | 1.35 | .50 | .93 |
| item 38 | 2.83 | 1.25 | .45 | .93 |
| item 39 | 3.55 | 1.21 | .49 | .93 |
| item 40 | 3.40 | 1.35 | .56 | .93 |
| item 41 | 1.61 | 1.16 | .35 | .93 |
| item 42 | 3.17 | 1.23 | .60 | .93 |
| item 43 | 3.47 | 1.27 | .54 | .93 |
| item 44 | 3.29 | 1.37 | .61 | .93 |
| item 45 | 2.08 | 1.36 | .53 | .93 |
| item 46 | 2.87 | 1.32 | .57 | .93 |
| item 47 | 2.00 | 1.20 | .63 | .93 |

*Note.* SD= Standard deviation, r_tot_= corrected item-total correlation,

𝜶= Cronbach’s Alpha.

**eAppendix 3.** Reliability analyses

**eTable 5.**

*Reliability coefficients of each BPDCL subscale for the German sample (N=257)*

|  | Cronbach’s Alpha | Guttman Lambda2 | McDonald’s Omega |
| --- | --- | --- | --- |
| Fear of abandonment | .79 | .80 | .79 |
| Interpersonal relationships | .68 | .69 | .69 |
| Identity disturbance | .80 | .81 | .80 |
| Impulsivity | .64 | .66 | .63 |
| Parasuicidal behavior | .68 | .68 | .69 |
| Affective instability | .77 | .77 | .78 |
| Emptiness | - | - | - |
| Anger | .71 | .73 | .76 |
| Paranoid and dissociative behavior | .83 | .84 | .82 |
| Total scale | .93 | .94 | - |

*Note.* Reliability coefficients for the emptiness subscale could not be calculated, as it consists of only one item. McDonald’s Omega could not always be calculated, as the assumptions were violated.

**eAppendix 4.** Convergent validity

**eTable 6.**

*Means and standard deviations of each instrument*

|  | N | Min | Max | Mean | SD |
| --- | --- | --- | --- | --- | --- |
| BPDSI- Abandonment | 257 | .00 | 9.14 | 3.19 | 1.84 |
| BPDSI- Interpersonal  relationships | 257 | .00 | 7.00 | 2.97 | 1.39 |
| BPDSI- Identity disturbance | 257 | .00 | 9.06 | 5.14 | 1.88 |
| BPDSI- Impulsivity | 257 | .00 | 5.64 | 1.75 | 1.08 |
| BPDSI- Parasuicidal b. | 257 | .00 | 6.62 | 1.29 | .98 |
| BPDSI- Affective instability | 257 | 2.20 | 10.00 | 6.85 | 1.80 |
| BPDSI- Emptiness | 257 | .00 | 10.00 | 5.92 | 2.02 |
| BPDSI- Anger | 257 | .00 | 7.83 | 3.28 | 1.74 |
| BPDSI- Dissoziation | 257 | .00 | 8.50 | 2.76 | 1.60 |
| BPDSI- Sum Score | 257 | 12.81 | 58.80 | 33.15 | 8.16 |
| EQ-5D-3L | 257 | -.16 | .98 | .65 | .22 |
| Total score of WSAS | 255 | 7.00 | 38.00 | 23.32 | 7.28 |
| Somatization scale BSI | 257 | .00 | 3.33 | 1.36 | .88 |
| Obsessive scale BSI | 257 | .00 | 4.00 | 2.21 | .83 |
| Insecurity scale BSI | 257 | .00 | 4.00 | 2.42 | .92 |
| Depressive scale BSI | 257 | .00 | 4.00 | 2.38 | .89 |
| Anxiety scale BSI | 257 | .00 | 4.00 | 1.81 | .95 |
| Hostility scale BSI | 257 | .00 | 4.00 | 1.76 | .90 |
| Phobic anxiety scale BSI | 257 | .00 | 4.00 | 1.50 | 1.03 |
| Paranoid scale BSI | 257 | .00 | 3.80 | 1.86 | .87 |
| Psychoticism scale BSI | 257 | .00 | 4.00 | 1.80 | .85 |
| BSI total score | 228 | 24.00 | 190.00 | 100.69 | 35.41 |
| WHO self-esteem | 257 | 1.00 | 4.00 | 1.98 | .69 |
| WHO Negative feelingss | 257 | 1.00 | 5.00 | 2.82 | 1.18 |
| WHO Positive feelings | 257 | 1.00 | 4.25 | 2.22 | .66 |
| WHO Physical | 257 | 4.17 | 87.50 | 40.62 | 15.53 |
| WHO Psychological | 257 | .00 | 90.00 | 31.15 | 14.93 |
| WHO Social | 257 | .00 | 91.67 | 39.62 | 20.25 |
| WHO Environment | 257 | 15.63 | 96.88 | 55.98 | 15.39 |

*Note*. N= sample size, Min= Minimum score, Max=maximum score, SD= standard deviation.

### ***Convergent validity***

The BPDCL total score correlated strongly with the BPDSI total score (.56). Correlations of the BPDCL subscales with their corresponding BPDSI subscales were strong for the *Impulsive* (.61) and *Parasuicidal* (.59) subscale only. Acceptable correlations were found for the *Abandonment* (.49), *Anger* (.52) and *Paranoid* (.51) subscales. The lowest correlation with the BPDSI was found for the *Social relationship* subscales (.30).

The BPDCL total score correlated strongly with the BSI-53 total score (.74). The *Affective instability* subscale correlated the strongest with the BSI total score (.71) and showed very strong correlations with *the Insecurity* (.65), *Depression* (.66) and the *Hostility* (.58) subscales of the BSI. *Impulsivity* did not correlate with any BSI subscale in an acceptable way (<.45). The BPDCL subscale *Abandonment* correlated strongly with the BSI subscales *Insecurity* (.55) and *Hostility* (.56). *Identity disturbance* correlated strongly with the *Insecurity* (.58), *Depression* (.60) and *Psychotic* (.59) BSI subscales. *Parasuicidal behavior* correlated strongly with the BSI *Depression* subscale (.58).  *Emptiness* correlated strongly with the BSI *Depression* scale (.60). *Anger* showed a very strong correlation with its corresponding BSI subscale *Hostility* (.70). *Paranoid ideation* had the highest correlations with the *Insecurity* (.57), *Paranoid* (.63) and *Psychotic* scales of the BSI (.61).

*Affective instability* and *Identity disturbance* correlated moderately with the *Self-esteem* subscale, assessed by the WHO. Scoring high on *Affective instability* or *Identity disturbance* was associated with lower *Self-esteem* (-.48).  Both BPDCL subscales did also correlate moderately with the *Psychological quality of life* WHO scale (.48 and 4.6, respectively). The BPDCL did not correlate strongly with any other WHO subscale, nore with the EuroQoL, WSAS total score or the WCCL (<.45).

### ***Conclusion***

To sum up, the German BPDCL possesses good psychometric qualities. The total scale reached a Cronbach’s Alpha of .93. All subscales demonstrated good reliability, except for *Interpersonal relationships* (.68), *Impulsivity* (.64) and *Parasuicidal behaviors* (.68).

The BPDCL showed weak to strong correlations with other mental health questionnaires. The BPDCL correlated strongly with the BPD-specific questionnaire BPDSI (.56). Unfortunately, the correlations across the corresponding subscales were rather weak to moderate. Only five BPDCL subscales showed correlations above .45 with their corresponding BPDSI scales. The BPDCL showed stronger correlations with the non-BPD specific questionnaire BSI-53 (.74). Corresponding subscales could be identified (e.g. BPDCL *Anger* and BSI *Hostility*). Correlations with the other questionnaires were rather weak. Known-groups validity could not be assessed, as only data on BPD patients was available.

**eTable 7.**

|  | BPDSI Abandonment | BPDSI  Relation | BPDSI Identity | BPDSI Impulsive | BPDSI  Parasuicidal | BPDSI  Affective | BPDSI  Emptiness | BPDSI Anger | BPDSI  Dissociative | BPDSI  Total score |
| --- | --- | --- | --- | --- | --- | --- | --- | --- | --- | --- |
| BPDCL subscales |  |  |  |  |  |  |  |  |  |  |
| Abandonment | **.49**** | .24** | .18** | .12 | .25** | .29** | .26** | .16* | .23* | **.45**** |
| Interpersonal relationships | .31** | **.30**** | .26** | .14** | .26** | .29** | .26** | .14* | .35** | **.45**** |
| Identity disturbance | .22** | .21** | **.38**** | .22** | .29** | .25** | .31** | .13* | .36** | **.46**** |
| Impulsivity | .12 | .17** | .14* | **.61**** | .21** | .18** | .26** | .20** | .19** | **.38**** |
| Parasuicidal behavior | .14* | .08 | .00 | .15* | **.59**** | .16* | .09 | -.03 | .17** | **.21**** |
| Affective instability | .33** | .20** | .14* | .09 | .31** | **.39**** | .27** | .15* | .31** | **.44**** |
| Emptiness | .19** | .10 | .16** | .16** | .21** | .30** | **.36**** | .08 | .18** | **.36**** |
| Anger | .22* | .29** | .11 | .19** | .17** | .34** | .20** | **.52**** | .22** | **.47**** |
| Paranoid ideation | .20** | .28** | .15* | .18** | .16* | .34** | .27** | .19** | **.51**** | **.46**** |
| Total score | **.36**** | **.30**** | **.24**** | **.28**** | **.31**** | **.37**** | **.34**** | **.25**** | **.40**** | **.56**** |

*Spearman’s Rho correlations of the German BPDCL and BPDSI*

*Note.* ** Correlation is significant at the 0.01 level (two-tailed). * Correlation is significant at the 0.05 level (two-tailed). Correlations without a star are not significant. Important findings are highlighted (bold and underlined). BPDCL= Borderline Personality Disorder Checklist, BPDSI= Borderline Personality DIsorder Severity Index. 

**eTable 8.**

|  | BSI  Somatic | BSI  Obsession | BSI  Insecurity | BSI  Depression | BSI  Anxiety | BSI  Hostility | BSI  Phobia | BSI Paranoid | BSI Psychotic | BSI  Total |
| --- | --- | --- | --- | --- | --- | --- | --- | --- | --- | --- |
| BPDCL subscales |  |  |  |  |  |  |  |  |  |  |
| Abandonment | .24** | .34** | .55** | .51** | .43** | .56** | .33** | .51** | .48** | **.57**** |
| Interpersonal relationships | .34** | .37** | .56** | .41** | .45** | .47** | .35** | .57** | .50** | **.57**** |
| Identity disturbance | .31** | .50** | .58** | .60** | .40** | .43** | .30** | .44** | .59** | **.58**** |
| Impulsivity | .23** | .31** | .24** | .32** | .25** | .30** | .14** | .29** | .35** | **.34**** |
| Parasuicidal behavior | .22** | .29** | .30** | .58** | .30** | .31** | .33** | .30** | .47** | **.49**** |
| Affective instability | .33** | .54** | **.65**** | **.66**** | **.54**** | .58** | .44** | .47** | .54** | **.71**** |
| Emptiness | .32** | .53** | .43** | .60** | .36** | .36** | .27** | .36** | .46** | **.54**** |
| Anger | .22** | .27** | .33** | .31** | .29** | **.70**** | .21** | .33** | .34** | **.43**** |
| Paranoid ideation | .46** | .54** | .57** | .46** | .53** | .47** | **.51**** | **.63**** | **.61**** | **.70**** |
| Total score | **.39**** | **.54**** | **.64**** | **.64**** | **.53**** | **.63**** | **.43**** | **.61**** | **.65**** | **.74**** |

*Spearman's Rho correlations of the German BPDCL and the BSI-53*

*Note.* ** Correlation is significant at the 0.01 level (two-tailed). * Correlation is significant at the 0.05 level (two-tailed). Correlations without a star are not significant. Important findings are highlighted (bold and underlined). BSI= Brief Symptom Inventory-53.

**eTable 9.**

*Spearman's Rho correlations of the German BPDCL and the WHOQoL subscales*

|  | WHO Self-esteem | WHO Negative feelings | WHO Positive feelings | WHO Physical | WHO Psychological | WHO   Social | WHO Environment |
| --- | --- | --- | --- | --- | --- | --- | --- |
| BPDCL subscales |  |  |  |  |  |  |  |
| Abandonment | -.33** | -.14* | -.22** | -.28** | -.27** | -.15* | -.19** |
| Interpersonal relationships | -.27** | -.15* | -.20** | -.18** | -.27** | -.23** | -.20** |
| Identity disturbance | **-.48**** | -.20** | -.37** | -.35** | **-.46**** | -.33** | -.21** |
| Impulsivity | -.18** | -.11 | -.17** | .,21** | -.28** | -.20** | -.12 |
| Parasuicidal behavior | -.25** | -.15* | -.29** | -.29** | -.33** | -.21 | -.08 |
| Affective instability | **-.48**** | -.21** | -.36** | -.41** | **-.48**** | -.12 | -.13* |
| Emptiness | -.33** | -.26** | -.35** | -.37** | -.38** | -.23** | -.19** |
| Anger | -.18** | -.22** | -.18** | -.20** | -.21** | -.09 | -.13* |
| Paranoid ideation | -.24** | -.24** | -.24** | -.24** | -.31** | -.16** | -.32** |
| Total score | **-.40**** | **-.23**** | **-.33**** | **-.36**** | **-.42**** | **-.24**** | **-.24**** |

*Note.* ** Correlation is significant at the 0.01 level (two-tailed). * Correlation is significant at the 0.05 level (two-tailed). Correlations without a star are not significant. Important findings are highlighted (bold and underlined). WHO= WHOQoL questionnaire.

**eTable 10.**

*Spearman's Rho correlations of the German BPDCL and the DBT-WCCL, WSAS and the EuroQoL*

*Note.* ** Correlation is significant at the 0.01 level (two-tailed). * Correlation is significant at the 0.05 level (two-tailed). Correlations without a star are not significant. Important findings are highlighted (bold and underlined). The German WCCL was rated on a different response scale compared to the Dutch and the English WCCL. The convergent validity across the BPDCL and the WCCL for the total sample was based on the English and Dutch WCCL only.

|  | WCCL  Skill use | WCCL  Dysfunct. coping | WCCL  Blaming others | WSAS  Total score | EQ-5D-3L |
| --- | --- | --- | --- | --- | --- |
| BPDCL subscales |  |  |  |  |  |
| Abandonment | .19* | .17* | .16* | .24** | -.26** |
| Interpersonal relationships | .22 | .14 | .23** | .22** | -.31** |
| Identity disturbance | .12 | .08 | .30** | .30** | -.27** |
| Impulsivity | .01 | .14 | .06 | .18** | -.17** |
| Parasuicidal behavior | -.04 | -.04 | .08 | .19** | -.26** |
| Affective instability | .08 | -.01 | .14 | .38** | -.44** |
| Emptiness | .04 | -.03 | .05 | .26** | -.31** |
| Anger | .14 | .15 | .18* | .17** | -.28** |
| Paranoid ideation | .16 | .11 | .28** | .30** | -.38** |
| Total score | **.17*** | **.14** | **.24**** | **.33**** | **-.37**** |
