# Supplementary material for: Psychometric Evaluation of the Borderline Personality Disorder Checklist
Source: Int J Methods Psychiatr Res. 2025 Sep 25;34(3):e70029. doi: 10.1002/mpr.70029 (PMC12461754; doi:10.1002/mpr.70029)
Supplement: Supplementary file 1 — Supporting Information S1 [file MPR-34-e70029-s001.zip › Wiley_SM6-English.docx]

**Supplementary material for the English dataset**

**eAppendix 1.** Descriptives of the English sample

**eTable 1.** Demographics

**eTable 2.** Clinical information

**eAppendix 2.** Item analyses of the English BPDCL

**eTable 3.** Results of Shapiro Wilk test

**eTable 4**. Results of individual item analyses

**eAppendix 3.** Reliability analyses of the English BPDCL

**eTable 5.** Reliability coefficients of each BPDCL subscales

**eAppendix 4**. Convergent validity of the English BPDCL

**eTable 6.** Means and standard deviations of each instrument

**eTable 7.** BPDCL and BPDSI

**eTable 8.** BPDCL and BSI-53

**eTable 9.** BPDCL and WHO-BREF

**eTable 10.** BPDCL and DBT-WCCL, WSAS and EuroQoL

This supplementary material has been provided by the authors to give readers additional information about their work.

**eAppendix 1.** Descriptives of the English sample

The English sample consisted of 97 BPD patients. The age ranged from 19 to 61, with a mean of 34.31 (SD=10.12).  The majority identified as female, came from Western Europe, was currently single and received sickness benefits. Comorbidities with other mental disorders were high, especially with affective-, anxiety- and substance use disorders.

**eTable 1.**

*Sociodemographic data of the English sample*

|  | *n (%* |
| --- | --- |
| Gender |  |
| Female | 79 (81.44) |
| Male | 18 (18.56) |
| Education |  |
| Primary | 10 (10.42) |
| Secondary | 34 (35.42) |
| Tertiary | 44 (45.83) |
| Other | 8 (8.33) |
| Marital status |  |
| Single | 63 (64.95) |
| Married or lasting relationship | 32 (32.99) |
| Other | 2 (2.06) |
| Ethnicity |  |
| Western Europe | 90 (92.78) |
| South America | 1 (1.03) |
| Africa | 1 (1.03) |
| Asia | 3 (3.09) |
| Mixed | 2 (2.06) |
| Employment |  |
| Homemaker | 15 (15.79) |
| Student | 6 (6.32) |
| Sickness benefits | 46 (48.42) |
| Working | 14 (14.74) |
| Unemployed | 8 (8.42) |
| Other | 6 (6.32) |
|  | *Mean (SD)* |
| Age | *34.31 (10.12)* |

*Note.* n=frequency, SD= standard deviation.

**eTable 2.**

*Clinical information of the English sample*

|  | n (%) |
| --- | --- |
| Axis I disorder |  |
| Affective | 89 (100) |
| Anxiety | 83 (100) |
| Substance use | 60 (100) |
| Eating | 22 (100) |
| Other | 41 (100) |
| Axis II disorder |  |
| Avoidant PD | 38 (43.18) |
| Dependent PD | 8 (9.09) |
| OCPD | 20 (22.73) |
| Paranoid PD | 26 (29.89) |
| Schizotyp PD | 1 (1.14) |
| Schizoid PD | 1 (1.14) |
| Borderline PD | 97 (100) |
| Unspecified PD | 13 (14.77) |

*Note.* PD= Personality Disorder,

n=Frequency.

**eAppendix 2.** Item analyses of the English BPDCL

According to the Shapiro-Wilk’s test and the visual inspection of the data, the assumption of normality of the data is not met (p<.001, see Table 3). The item analysis can be seen in Table 4. Item means range from 1.17 (item 35) to 4.17 (item 11) for the English sample. Some items were centered at one end of the response scale. All item responses from 1 to 5 were scored for each item. The mean inter item correlation is 0.27, which is within the predefined range of 0.20 to 0.40. The variance of the mean-inter-item correlation (s²=.03) is rather small. Thus, the individual inter-item correlations do not vary strongly. Item 7, 8, 12, 17 and 35 have corrected item total correlations below 0.30. Cronbach’s Alpha if those items were deleted do not differ from the initial Cronbach’s Alpha of the total scale (Cronbach’s Alpha=0.95).  Thus, this small deviation from .3 can be ignored, as the scale reliability is not affected by those items. The total scale mean was 125.35 (SD=31.14). The highest mean score of the total scale was 204 and the lowest was 59.

Reliability coefficients for the English sample can be seen in Table 5. Reliability coefficients of each subscale were higher than the predefined value of 0.70, except for the *Impulsivity* subscale (Cronbach’s Alpha= 0.66). Cronbach’s Alpha, Guttman’s Lamda2 and McDonal’s Omega did not differ greatly from one another. McDonald’s Omega should be looked at with caution, as the assumption of normality of the data is not met.

**eTable 3.**

*Test of normality of the English BPDCL*

| Shapiro Wilk | | | |
| --- | --- | --- | --- |
|  | Statistic | df | Sig. |
| item 1 | .91 | 97 | <,001 |
| item 2 | .81 | 97 | <,001 |
| item 3 | .86 | 97 | <,001 |
| item 4 | .89 | 97 | <,001 |
| item 5 | .47 | 97 | <,001 |
| item 6 | .84 | 97 | <,001 |
| item 7 | .75 | 97 | <,001 |
| item 8 | .34 | 97 | <,001 |
| item 9 | .89 | 97 | <,001 |
| item 10 | .88 | 97 | <,001 |
| item 11 | .79 | 97 | <,001 |
| item 12 | .77 | 97 | <,001 |
| item 13 | .89 | 97 | <,001 |
| item 14 | .89 | 97 | <,001 |
| item 15 | .86 | 97 | <,001 |
| item 16 | .90 | 97 | <,001 |
| item 17 | .63 | 97 | <,001 |
| item 18 | .90 | 97 | <,001 |
| item 19 | .90 | 97 | <,001 |
| item 20 | .78 | 97 | <,001 |
| item 21 | .87 | 97 | <,001 |
| item 22 | .64 | 97 | <,001 |
| item 23 | .68 | 97 | <,001 |
| item 24 | .77 | 97 | <,001 |
| item 25 | .88 | 97 | <,001 |
| item 26 | .71 | 97 | <,001 |
| item 27 | .84 | 97 | <,001 |
| item 28 | .59 | 97 | <,001 |
| item 29 | .87 | 97 | <,001 |
| item 30 | .90 | 97 | <,001 |
| item 31 | .84 | 97 | <,001 |
| item 32 | .88 | 97 | <,001 |
| item 33 | .89 | 97 | <,001 |
| item 34 | .89 | 97 | <,001 |
| item 35 | .36 | 97 | <,001 |
| item 36 | .80 | 97 | <,001 |
| item 37 | .76 | 97 | <,001 |
| item 38 | .91 | 97 | <,001 |
| item 39 | .90 | 97 | <,001 |
| item 40 | .89 | 97 | <,001 |
| item 41 | .66 | 97 | <,001 |
| item 42 | .90 | 97 | <,001 |
| item 43 | .89 | 97 | <,001 |
| item 44 | .90 | 97 | <,001 |
| item 45 | .75 | 97 | <,001 |
| item 46 | .91 | 97 | <,001 |
| item 47 | .76 | 97 | <,001 |

*Note*. df= degress of freedom, Sig.=Significance. Cases were excluded

listwise.

**eTable 4.**

*Item analysis for the English BPDCL*

|  | Mean | SD | r_tot_ | 𝜶 if Item Deleted |
| --- | --- | --- | --- | --- |
| item 1 | 3.14 | 1.21 | .36 | .95 |
| item 2 | 4.03 | 1.07 | .59 | .95 |
| item 3 | 2.43 | 1.33 | .68 | .95 |
| item 4 | 2.58 | 1.24 | .55 | .95 |
| item 5 | 1.43 | 1.04 | .55 | .95 |
| item 6 | 2.31 | 1.33 | .52 | .95 |
| item 7 | 2.00 | 1.31 | .27 | .95 |
| item 8 | 1.19 | .62 | .09 | .95 |
| item 9 | 2.54 | 1.23 | .47 | .95 |
| item 10 | 3.64 | 1.21 | .69 | .95 |
| item 11 | 4.16 | .92 | .59 | .95 |
| item 12 | 1.96 | 1.23 | .19 | .95 |
| item 13 | 3.37 | 1.30 | .47 | .95 |
| item 14 | 2.82 | 1.38 | .77 | .94 |
| item 15 | 3.76 | 1.11 | .47 | .95 |
| item 16 | 2.71 | 1.22 | .54 | .95 |
| item 17 | 1.70 | 1.21 | .22 | .95 |
| item 18 | 3.10 | 1.36 | .67 | .95 |
| item 19 | 3.40 | 1.23 | .66 | .95 |
| item 20 | 2.02 | 1.24 | .51 | .95 |
| item 21 | 3.52 | 1.32 | .65 | .95 |
| item 22 | 1.67 | 1.13 | .31 | .95 |
| item 23 | 1.70 | 1.09 | .35 | .95 |
| item 24 | 1.87 | 1.10 | .63 | .95 |
| item 25 | 3.73 | 1.06 | .64 | .95 |
| item 26 | 1.76 | 1.13 | .41 | .95 |
| item 27 | 2.43 | 1.41 | .63 | .95 |
| item 28 | 1.53 | 1.01 | .54 | .95 |
| item 29 | 2.64 | 1.42 | .33 | .95 |
| item 30 | 3.21 | 1.35 | .64 | .95 |
| item 31 | 2.35 | 1.32 | .63 | .95 |
| item 32 | 3.04 | 1.41 | .62 | .95 |
| item 33 | 3.39 | 1.30 | .57 | .95 |
| item 34 | 3.32 | 1.30 | .56 | .95 |
| item 35 | 1.16 | .51 | -.14 | .95 |
| item 36 | 4.08 | 1.04 | .52 | .95 |
| item 37 | 1.88 | 1.15 | .64 | .95 |
| item 38 | 3.00 | 1.26 | .35 | .95 |
| item 39 | 3.05 | 1.35 | .54 | .95 |
| item 40 | 3.13 | 1.42 | .51 | .95 |
| item 41 | 1.84 | 1.34 | .42 | .95 |
| item 42 | 2.87 | 1.33 | .66 | .95 |
| item 43 | 3.55 | 1.13 | .69 | .95 |
| item 44 | 3.26 | 1.22 | .63 | .95 |
| item 45 | 2.11 | 1.44 | .58 | .95 |
| item 46 | 3.00 | 1.25 | .63 | .95 |
| item 47 | 1.96 | 1.26 | .46 | .95 |

*Note.* SD= Standard deviation, r_tot_= corrected item-total correlation, 𝜶= Cronbach’s Alpha.

**eAppendix 3.** Reliability analyses of the English BPDCL

**eTable 5.**

*Reliability coefficients of each BPDCL subscale for the English sample*

|  | Cronbach’s Alpha | Guttman Lamda2 | McDonald’s Omega |
| --- | --- | --- | --- |
| Fear of Abandonment | .84 | .85 | .84 |
| Interpersonal relationships | .75 | .75 | .75 |
| Identity disturbance | .84 | .85 | .85 |
| Impulsivity | .66 | .69 | - |
| Parasuicidal behavior | .79 | .79 | .80 |
| Affective instability | .76 | .77 | .76 |
| Emptiness | - | - | - |
| Anger | .80 | .81 | .81 |
| Paranoid and dissociative behavior | .81 | .83 | .79 |
| Total scale | .95 | .95 | .96 |

*Note.* McDonald’s Omega should be looked at cautiously, as the assumption of normality of the data is violated. The reliability coefficients for the emptiness subscale could not be calculated, as it consists of only one item.

**eAppendix 4**. Convergent validity of the English BPDCL

### ***Convergent validity***

Means and standard deviations of the other psychological instruments are presented in table 6. Since the normality of the data was not met, Spearman’s Rho was calculated to estimate the convergent validity of the English BPDCL. Results can be seen in Table 6 to 10.

The BPDCL total score correlated strongly with the BPDSI total score (.66). Correlations of the BPDCL subscales with their corresponding BPDSI subscales were strong for the *Abandonment* (.59), *Impulsivity* (.67), *Parasuicidal* (.60) and *Anger* (.58) subscales. Acceptable correlations were found for the *Identity disturbance* (.48), *Affective instability* (.50) and *Paranoid ideation* (.45) subscales. The lowest correlation with the corresponding BPDSI subscale was found for the *Emptiness* subscale (.30). Correlations with non-corresponding subscales were weak in most of the times (<.45), except for BPDCL *Anger* and BPDSI *Affective instability* (.54), as well as BPDCL *Affective instability* and BPDSI *identity disturbance* (.51).

The BPDCL total score correlated strongly with the BSI-53 total score (.83). The *Affective instability* subscale correlated the strongest with the BSI total score (.76) and showed very strong correlations with almost all BSI subscales. *Impulsivity* did not correlate with any BSI subscale in an acceptable way (<.45). The BPDCL subscale *Abandonment* correlated the strongest with the BSI subscales *Insecurity* (.71) and *Psychotic* (.74). *Interpersonal relationships* correlated the strongest with BSI *Insecurity* (.68) and *Psychotic* (.69). *Identity disturbance* correlated the strongest with the *Insecurity* (.67) and *Psychotic* (.68) BSI subscales. *Parasuicidal* behavior correlated strongly with the BSI *Depression* subscale (.55). *Affective instability* correlated the strongest with BSI *Depression* (.71) and BSI *Anxiety* (.73). *Emptiness* correlated the strongest with the BSI *Depression* scale (.61). *Anger* showed a very strong correlation with its corresponding BSI subscale *Hostility* (.77). *Paranoid ideation* had the highest correlation with its corresponding BSI scale *Paranoid* (.69).

The WHO *Physical, Psychological, Social, self-esteem and Positive* feelings scales did not correlate strongly with the BPDCL total score (<.45). The BPDCL *Anger* score correlated strongly and negatively with the WHO *environment scale* (-.50). The BPDCL *interpersonal relationship* did not correlate acceptably with the *Social quality of life* (.41). *The* WHO *negative feeling* scale did correlate the strongest with the *Affective instability* (.63) and the *Anger* scale (-.50).

The WCCL scale *Blaming others* correlated strongly with the BPDCL *Anger* scale (.70). The BPDCL *Anger* scale correlated moderately with the WCCL *Dysfunctional coping* scale (.51). The BPDCL did not correlate acceptably with the EuroQoL, nor with the WSAS total score.

**eTable 6.**

*Means and standard deviations of other psychological instruments for the English sample*

|  | N | Minimum | Maximum | Mean | SD |
| --- | --- | --- | --- | --- | --- |
| BPDSI Abandonment | 97 | 0.00 | 8.86 | 3.14 | 2.17 |
| BPDSI Interpersonal | 97 | 0.00 | 7.63 | 2.69 | 1.62 |
| BPDSI Identity | 97 | 1.25 | 9.69 | 5.38 | 1.81 |
| BPDSI Impulsivity | 97 | 0.27 | 4.91 | 2.05 | 1.04 |
| BPDSI Parasuicidal | 97 | 0.00 | 6.62 | 1.61 | 1.14 |
| BPDSI Affective | 97 | 2.20 | 10.00 | 7.81 | 1.83 |
| BPDSI Emptiness | 97 | 0.00 | 10.00 | 6.94 | 2.00 |
| BPDSI Anger | 97 | 0.00 | 9.83 | 2.73 | 2.09 |
| BPDSI Dissociation | 97 | 0.00 | 10.00 | 3.33 | 1.92 |
| BPDSI Total Score | 97 | 20.09 | 69.63 | 35.68 | 9.90 |
| Somatization scale BSI | 97 | .00 | 3.86 | 1.31 | .91 |
| Obsessive scale BSI | 97 | .33 | 4.00 | 2.54 | .84 |
| Insecurity scale BSI | 97 | .00 | 4.00 | 2.52 | .95 |
| Depressive scale BSI | 97 | .50 | 4.00 | 2.70 | .81 |
| Anxiety scale BSI | 97 | .17 | 4.00 | 2.17 | .90 |
| Hostility scale BSI | 97 | .00 | 4.00 | 1.62 | 1.00 |
| Phobic anxiety scale BSI | 97 | .00 | 4.00 | 1.91 | 1.16 |
| Paranoid scale BSI | 97 | .00 | 4.00 | 1.92 | .99 |
| Psychoticism scale BSI | 97 | .40 | 4.00 | 2.03 | .80 |
| Sumscore BSI | 97 | 20.00 | 209.00 | 109.97 | 38.11 |
| WHO Self-esteem | 97 | 1.00 | 4.00 | 1.91 | 0.67 |
| WHO Positive feelings | 97 | 1.00 | 4.00 | 1.95 | 0.53 |
| WHO Negative feelings | 97 | 2.25 | 5.00 | 4.12 | 0.62 |
| WHO Physical | 97 | 14.29 | 67.86 | 38.00 | 10.48 |
| WHO Psychological | 97 | 12.50 | 66.67 | 32.99 | 11.28 |
| WHO Social | 97 | 0.00 | 83.33 | 31.49 | 21.42 |
| WHO Environment | 97 | 6.25 | 87.50 | 47.97 | 15.97 |
| DBT-WCCL Skill Use | 63 | 0.13 | 2.89 | 1.38 | 0.52 |
| DBT-WCCL Dysfunctional coping | 63 | 0.67 | 3.00 | 2.43 | 0.42 |
| DBT-WCCL Blaming others | 63 | 0.00 | 3.00 | 1.73 | 0.73 |
| Total score of WSAS | 97 | 4.00 | 40.00 | 25.59 | 7.45 |
| EQ-5D-3L | 97 | -0.06 | 0.97 | .61 | 0.19 |

**eTable 7.**

*Spearman’s Rho correlations of the English BPDCL and the BPDSI*

|  | BPDSI Abandonment | BPDSI  Relation | BPDSI Identity | BPDSI Impulsive | BPDSI  Parasuicid. | BPDSI  Affective | BPDSI  Emptiness | BPDSI Anger | BPDSI  Dissociative | BPDSI  Total |
| --- | --- | --- | --- | --- | --- | --- | --- | --- | --- | --- |
| BPDCL subscales |  |  |  |  |  |  |  |  |  |  |
| Abandonment | **.59**** | .43** | .40** | .27** | .41** | .37** | .21** | .34** | .28** | **.59**** |
| Interpersonal relationships | .23** | **.43**** | .30** | .17** | .33** | .21* | .19 | .29** | .27** | **.42**** |
| Identity disturbance | .34** | .36** | **.48**** | .26** | .38** | .31** | .29** | .28** | .32** | **.55**** |
| Impulsivity | .20* | .33* | .31** | **.67**** | .20 | .20 | .19 | .29** | .27** | **.42**** |
| Parasuicidal behavior | .25* | .25* | .27** | .14 | **.60**** | .24* | .20 | .16 | .20 | **.40** |
| Affective instability | .25* | .34** | .51** | .16 | .39** | **.50**** | .31** | .33** | .25* | **.57**** |
| Emptiness | .20 | .24* | .34** | .09 | .30** | .28** | **.30**** | .29** | .20* | **.44**** |
| Anger | .37** | .38** | .41** | .31** | .30** | .54** | .30** | **.58**** | .33** | **.63**** |
| Paranoid ideation | .16 | .37** | .28** | .17 | .29** | .30** | .25** | .39** | **.45**** | **.49**** |
| Total score | **.40**** | **.46**** | **.49**** | **.35**** | **.43**** | **.43**** | **.30**** | **.42**** | **.38**** | **.66**** |

**Note.** ** Correlation is significant at the 0.01 level (two-tailed). * Correlation is significant at the 0.05 level (two-tailed). Correlations without a star are not significant. Important findings are highlighted (bold and underlined). BPDCL= Borderline Personality Disorder Checklist, BPDSI= Borderline Personality Disorder Severity Index.

**eTable 8.**

*Spearman's Rho correlations of the English BPDCL and the BSI-53*

|  | BSI Somatization | BSI Obsession | BSI Insecurity | BSI Depression | BSI  Anxiety | BSI  Hostility | BSI Phobia | BSI Paranoid | BSI Psychotic | BSI Total |
| --- | --- | --- | --- | --- | --- | --- | --- | --- | --- | --- |
| BPDCL subscales |  |  |  |  |  |  |  |  |  |  |
| Abandonment | .41** | .53** | **.71**** | .59** | .62** | .57** | .43** | .53** | .74** | .71** |
| Interpersonal relationships | .46** | .53** | .68** | .53** | .56** | .55** | .37** | .64** | .69** | .70** |
| Identity disturbance | .44** | .55** | **.67**** | .50** | .49** | .50** | .31** | .56** | .**68**** | .65** |
| Impulsivity | .22* | .41** | .30** | .18 | .36** | .35** | .16** | .30** | .38** | .36** |
| Parasuicidal behavior | .26* | .39** | .41** | **.55**** | .38** | .37** | .18 | .27** | .49** | .45** |
| Affective instability | .46** | .63** | .66** | **.71**** | **.73**** | .60** | .47** | .47** | .68** | .76** |
| Emptiness | .42** | .50** | .56** | .61** | .42** | .49** | .29** | .47** | .58** | .59** |
| Anger | .37** | .52** | .63** | .51** | .60** | **.77**** | .48** | .61** | .65** | .71** |
| Paranoid ideation | .55** | **.65**** | .60** | .48** | .52** | .65** | .47** | **.69**** | .63** | .73** |
| Total score | **.52**** | **.69**** | **.77**** | **.65**** | **.69**** | **.70**** | **.47**** | **.66**** | **.81**** | **.83**** |

*Note.* ** Correlation is significant at the 0.01 level (two-tailed). * Correlation is significant at the 0.05 level (two-tailed). Correlations without a star are not significant. Important findings are highlighted (bold and underlined). BSI= Brief Symptom Inventory-53.

**eTable 9.**

*Spearman’s Rho correlations of the English BPDCL and WHO subscales*

|  | WHO  Physical | WHO Psychological | WHO  Social | WHO  Environment | WHO  Self-esteem | WHO Negative | WHO  Positive |
| --- | --- | --- | --- | --- | --- | --- | --- |
| BPDCL subscales |  |  |  |  |  |  |  |
| Abandonment | -.26* | -.35** | -.38** | -.44** | -.37** | .47** | -.25** |
| Interpersonal relationships | -.20* | -.29** | **-.41**** | -.37** | -.18 | .40** | -.22* |
| Identity disturbance | -.31** | -.29** | -.22* | -.37** | -.39** | .44** | -.36** |
| Impulsivity | -.22* | -.08 | -.06 | -.38** | -.09 | .13 | -.01 |
| Parasuicidal behavior | -.16 | -.27** | -.21* | -.31** | -.35** | .27** | -.16 |
| Affective instability | -.29** | -.27** | -.23* | -.34** | -.34** | **.63**** | -.33** |
| Emptiness | -.25 | -.36** | -.30** | -.30** | -.31** | -.40** | -.34** |
| Anger | -.28** | -.34** | -.25* | **-.50**** | -.36** | **-.50**** | -.36** |
| Paranoid ideation | -.24* | -.31** | -.19 | -.40** | -.19 | .45** | -.30** |
| Total score | **-.34**** | **-.37**** | **-.32**** | **-.50**** | **-.38**** | **.53**** | **-.36**** |

*Note.* ** Correlation is significant at the 0.01 level (two-tailed). * Correlation is significant at the 0.05 level (two-tailed). Correlations without a star are not significant. Important findings are highlighted (bold and underlined).BPDCL= Borderline Personality Disorder Checklist, WHO= World Health Organization Quality of Life

**eTable 10.**

*Spearman’s Rho correlations of the English BPDCL and DBT-WCCL subscales, WSAS Total Score and the EuroQoL index*

|  | WCCL Skill Use | WCCL Dysfunct. | WCCL Blaming | WSAS  Total score | EQ5D  Index |
| --- | --- | --- | --- | --- | --- |
| BPDCL subscales |  |  |  |  |  |
| Abandonment | -.06 | .47** | .44** | .11 | -.18 |
| Interpersonal relationships | .14 | .48** | .46** | .08 | -.26** |
| Identity disturbance | -.17 | .47** | .43** | .05 | -.18 |
| Impulsivity | -.12 | .09 | .35** | .14 | .10 |
| Parasuicidal behavior | -.13 | .08 | .11 | .01 | -.04 |
| Affective instability | .00 | .41** | .39** | .12 | -.31** |
| Emptiness | -.12 | .34** | .30* | .14 | -.21* |
| Anger | .02 | .**51**** | **.70**** | .14 | -.30** |
| Paranoid ideation | .12 | .43** | .50** | .05 | -.31** |
| Total score | **-.04** | **.50**** | **.55**** | **.11** | **-.24*** |

*Note.* ** Correlation is significant at the 0.01 level (two-tailed). * Correlation is significant at the 0.05 level (two-tailed). Correlations without a star are not significant. Important findings are highlighted (bold and underlined).BPDCL= Borderline Personality Disorder Checklist, DBT-WCCL= Dialectical Behavioral Therapy- Ways of Coping Checklist, WSAS= Work and Social Adjustment Scale, EQ5D= European Quality of Life index.

### ***Conclusion***

The English sample consisted of 97 BPD patients. The Cronbach’s Alpha of the total scale was .94. The reliability coefficients of the subscales ranged from .66 (*Impulsivity*) to .84 (*Fear of abandonment* and *Identity disturbance*). Convergent validity was mainly good to excellent with corresponding scales of the BSI, BPDSI, WHO and WCCL. Further investigation with respect to the known-groups validity of the English BPDCL is needed.
