# Supplementary material for: Psychometric Evaluation of the Borderline Personality Disorder Checklist
Source: Int J Methods Psychiatr Res. 2025 Sep 25;34(3):e70029. doi: 10.1002/mpr.70029 (PMC12461754; doi:10.1002/mpr.70029)
Supplement: Supplementary file 1 — Supporting Information S1 [file MPR-34-e70029-s001.zip › Wiley_SM7- Greek .docx]

**Supplementary material for the Greek dataset**

**eAppendix 1.** Descriptives of the Greek sample

**eTable 1.** Demographics

**eTable 2.** Clinical information

**eAppendix 2.** Item analyses of the Greek BPDCL

**eTable 3.** Results of Shapiro Wilk test

**eTable 4**. Results of individual item analyses

**eAppendix 3.** Reliability analyses of the Greek BPDCL

**eTable 5.** Reliability coefficients of each BPDCL subscales

**eAppendix 4.** Convergent validity analyses of the Greek BPDCL

**eTable 6.** Means and standard deviations of the psychological instruments

**eTable 7.** BPDCL and BPDSI

**eTable 8.** BPDCL and BSI-53

**eTable 9.** BPDCL and WHO-BREF

**eTable 10.** BPDCL and WSAS

This supplementary material has been provided by the authors to give readers additional information about their work.

**eAppendix 1.** Descriptives of the Greek sample

The Greek sample consists of 32 BPD patients. Age ranged from 19 to 49. All participants identified as female and came from western Europe. The majority was single and was currently working. Comorbidities with other mental health disorders were high, especially with current affective and anxiety disorders.

**eTable 1.**

*Sociodemographic data of the Greek sample*

|  | BPPD  N=32 |
| --- | --- |
|  | n |
| Gender |  |
| Female | 32 (100) |
| Education |  |
| Primary | 1 (3.13) |
| Secondary | 12 (37.50) |
| Tertiary | 19 (59.38) |
| Marital status |  |
| Single | 30 (93.75) |
| Married or lasting  relationship | 2 (6.25) |
| Ethnicity |  |
| Western Europe | 32 (100) |
| Employment |  |
| Homemaker | 5 (15.63) |
| Student | 7 (21.88) |
| Working | 19 (59.38) |
| No legal income or benefits | 1 (3.13) |
|  | Mean (SD) |
| Age | 32.72 (8.24) |

*Note.* n=frequency, SD= standard deviation. Only

valid percentages are reported.

**eTable 2.**

*Clinical information of the Greek sample*

|  | BPD  N=32 |
| --- | --- |
|  | n |
| Axis I disorder |  |
| Affective | 20 (100) |
| Anxiety | 12 (100) |
| Substance use | 9 (100) |
| Eating | 9 (100) |
| Other | 8 (100) |
| Axis II disorder |  |
| Avoidant | 9 (29.03) |
| Dependent | 4 (12.50) |
| OCPD | 3 (9.38) |
| Paranoid | 4 (12.00) |
| Schizoid | 1 (3.13) |
| Histrionic | 1 (3.13) |
| Borderline | 32 (100) |
| Unspecified PD | 3 (9.38) |

*Note.* OCPD= Obsessive-Compulsive Personality

Disorder. Only valid percentages are reported.

**eAppendix 2.** Item analyses of the Greek BPDCL

According to the Shapiro-Wilk’s test and the visual inspection of the data, the assumption of normality of the data is not met (p<.008, see Table 3). The item analysis is presented in Table 4. The item means range from 1.03 (item 8) to 4.34 (item 36). Not all response options from 1 to 5 have been used (e.g. Item 2 was scored from 2 to 5 only). The item means were centered for some items. The mean inter item correlation is 0.28, which is within the predefined range of 0.20 to 0.40. Items 22, 23, 24, 26, 29 and 35 have corrected item correlations below 0.30. Cronbach’s Alpha if those items were deleted does not differ from the initial Cronbach’s Alpha of the total scale (Cronbach’s Alpha=0.95). The total scale score ranged from 68 to 192, with a mean of 131.97 (SD= 30.50). Reliability coefficients were above 0.70 for each BPDCL subscale, except parasuicidal behavior (Cronbach’s Alpha= 0.63). Guttman’s Lamda was slightly higher than Cronbach’s Alpha. McDonald’s Omega should be looked at with caution, as the assumption of normality is violated. Reliability coefficients can be seen in Table 5. Validity analyses were not applied, as the sample size was too small, in order to obtain valid results.

**eTable 3.**

*Normal distribution of the Greek BPDCL (N=32)*

| Shapiro-Wilk | | | |
| --- | --- | --- | --- |
| BPDCL Items | Statistic | df | Sig. |
| item 1 | .90 | 32.00 | .005 |
| item 2 | .81 | 32.00 | <.001 |
| item 3 | .89 | 32.00 | .003 |
| item 4 | .89 | 32.00 | .003 |
| item 5 | .83 | 32.00 | <.001 |
| item 6 | .70 | 32.00 | <.001 |
| item 7 | .80 | 32.00 | <.001 |
| item 8 | .17 | 32.00 | <.001 |
| item 9 | .80 | 32.00 | <.001 |
| item 10 | .82 | 32.00 | <.001 |
| item 11 | .77 | 32.00 | <.001 |
| item 12 | .58 | 32.00 | <.001 |
| item 13 | .83 | 32.00 | <.001 |
| item 14 | .84 | 32.00 | <.001 |
| item 15 | .83 | 32.00 | <.001 |
| item 16 | .86 | 32.00 | <.001 |
| item 17 | .49 | 32.00 | <.001 |
| item 18 | .78 | 32.00 | <.001 |
| item 19 | .86 | 32.00 | <.001 |
| item 20 | .89 | 32.00 | .005 |
| item 21 | .88 | 32.00 | .002 |
| item 22 | .63 | 32.00 | <.001 |
| item 23 | .72 | 32.00 | <.001 |
| item 24 | .71 | 32.00 | <.001 |
| item 25 | .82 | 32.00 | <.001 |
| item 26 | .51 | 32.00 | <.001 |
| item 27 | .70 | 32.00 | <.001 |
| item 28 | .65 | 32.00 | <.001 |
| item 29 | .87 | 32.00 | .001 |
| item 30 | .87 | 32.00 | .001 |
| item 31 | .36 | 32.00 | <.001 |
| item 32 | .86 | 32.00 | <.001 |
| item 33 | .86 | 32.00 | <.001 |
| item 34 | .83 | 32.00 | <.001 |
| item 35 | .37 | 32.00 | <.001 |
| item 36 | .67 | 32.00 | <.001 |
| item 37 | .76 | 32.00 | <.001 |
| item 38 | .90 | 32.00 | .008 |
| item 39 | .90 | 32.00 | .005 |
| item 40 | .89 | 32.00 | .005 |
| item 41 | .62 | 32.00 | <.001 |
| item 42 | .91 | 32.00 | .010 |
| item 43 | .86 | 32.00 | <.001 |
| item 44 | .86 | 32.00 | <.001 |
| item 45 | .86 | 32.00 | <.001 |
| item 46 | .84 | 32.00 | <.001 |
| item 47 | .47 | 32.00 | <.001 |

*Note.* df=degree of freedom, Sig=Significance.

**eTable 4.**

*Item analysis of the Greek BPDCL*

| BPDCL item | Mean | SD | r_tot_ | 𝜶 _if Item Deleted_ |
| --- | --- | --- | --- | --- |
| item 1 | 2.81 | 1.31 | .45 | .95 |
| item 2 | 4.16 | .88 | .71 | .95 |
| item 3 | 3.41 | 1.21 | .53 | .95 |
| item 4 | 2.44 | 1.22 | .48 | .95 |
| item 5 | 2.47 | 1.50 | .59 | .95 |
| item 6 | 1.94 | 1.37 | .59 | .95 |
| item 7 | 2.03 | 1.20 | .36 | .95 |
| item 8 | 1.03 | .18 | .35 | .95 |
| item 9 | 2.31 | 1.49 | .49 | .95 |
| item 10 | 3.72 | 1.35 | .66 | .95 |
| item 11 | 4.25 | .95 | .58 | .95 |
| item 12 | 1.44 | .84 | .32 | .95 |
| item 13 | 3.84 | 1.14 | .50 | .95 |
| item 14 | 3.50 | 1.32 | .71 | .94 |
| item 15 | 3.94 | 1.08 | .74 | .95 |
| item 16 | 3.41 | 1.34 | .58 | .95 |
| item 17 | 1.44 | 1.05 | .55 | .95 |
| item 18 | 4.09 | 1.00 | .74 | .95 |
| item 19 | 3.84 | 1.08 | .83 | .94 |
| item 20 | 3.03 | 1.28 | .54 | .95 |
| item 21 | 3.63 | 1.10 | .73 | .95 |
| item 22 | 1.84 | 1.42 | .10 | .95 |
| item 23 | 1.72 | 1.05 | .20 | .95 |
| item 24 | 1.88 | 1.21 | .19 | .95 |
| item 25 | 4.06 | .95 | .42 | .95 |
| item 26 | 1.38 | .83 | .15 | .95 |
| item 27 | 1.97 | 1.43 | .60 | .95 |
| item 28 | 1.69 | 1.18 | .47 | .95 |
| item 29 | 2.94 | 1.41 | .21 | .95 |
| item 30 | 3.59 | 1.04 | .74 | .95 |
| item 31 | 1.19 | .59 | .37 | .95 |
| item 32 | 3.44 | 1.34 | .61 | .95 |
| item 33 | 3.53 | 1.14 | .59 | .95 |
| item 34 | 3.88 | 1.04 | .75 | .95 |
| item 35 | 1.25 | .76 | .23 | .95 |
| item 36 | 4.34 | 1.07 | .66 | .95 |
| item 37 | 2.34 | 1.60 | .56 | .95 |
| item 38 | 2.69 | 1.26 | .35 | .95 |
| item 39 | 3.19 | 1.26 | .69 | .95 |
| item 40 | 3.63 | 1.07 | .42 | .95 |
| item 41 | 1.91 | 1.51 | .52 | .95 |
| item 42 | 3.22 | 1.21 | .81 | .94 |
| item 43 | 3.22 | 1.50 | .49 | .95 |
| item 44 | 3.19 | 1.51 | .76 | .94 |
| item 45 | 2.28 | 1.28 | .53 | .95 |
| item 46 | 3.44 | 1.41 | .55 | .95 |
| item 47 | 1.47 | 1.14 | .39 | .95 |

*Note.* SD= Standard deviation, rtot= corrected item-total correlation,

𝜶= Cronbach’s Alpha.

**eAppendix 3.** Reliability analyses of the Greek BPDCL

**eTable 5.**

*Reliability coefficients of each BPDCL subscale for the Greek sample*

|  | Cronbach’s Alpha | Guttman Lamda2 | McDonald’sOmega |
| --- | --- | --- | --- |
| Fear of Abandonment | .81 | .81 | .80 |
| Interpersonal relationships | .69 | .70 | .72 |
| Identity disturbance | .86 | .87 | .86 |
| Impulsivity | .69 | .73 | .67 |
| Parasuicidal behaviour | .63 | .64 | .67 |
| Affective instability | .74 | .75 | .76 |
| Emptiness | - | - | - |
| Anger | .87 | .88 | .89 |
| Paranoid and dissociative behavior | .81 | .82 | .79 |
| Total scale | .95 | .95 | .94 |

*Note.* McDonald’s Omega should be looked at cautiously, as the assumption of normality of the data is violated. The reliability coefficients for the emptiness subscale could not be calculated, as it consists of only one item.

**eAppendix 4.** Convergent validity analyses of the Greek BPDCL

### ***Convergent validity***

Means and standard deviations of the other psychological instruments are presented in table 6. Since the normality of the data was not met, Spearman’s Rho was calculated to estimate the convergent validity of the Greek BPDCL. Results can be seen in Table 6 to 10.

The BPDCL total score correlated strongly with the BPDSI total score (.78). Correlations of the BPDCL subscales with their corresponding BPDSI subscales were strong for the *Abandonment* (.70), *Impulsivity* (.62), *Parasuicidal* (.81) and *Anger* (.77) subscales. Acceptable correlations were found for the *Identity disturbance* (.51) and *Paranoid ideation* (.46) subscales. The lowest correlation with the corresponding BPDSI subscale was found for the *Emptiness* subscale (.24). Correlations with non-corresponding subscales were weak in many cases (<.45).

The BPDCL total score correlated strongly with the BSI-53 total score (.59). The *Affective instability* subscale correlated the strongest with the BSI total score (.76) and showed very strong correlations with several BSI subscales. *Impulsivity* did not correlate with any BSI subscale in an acceptable way (<.45). The BPDCL subscale *Abandonment* correlated the strongest with the BSI subscales *Insecurity* (.59). *Interpersonal relationships* correlated the strongest with BSI *Paranoid ideation* (.68) and *Psychotic* (.68). *Identity disturbance* correlated the strongest with the *Insecurity* (.62) and Psychotic (.62) BSI subscales. *Parasuicidal* behavior correlated strongly with the BSI *Obsession* subscale (.65) and *Depression* (.60). *Affective instability* correlated the strongest with BSI *Obsession* (.67) and several other scales, such as *Depression, Hostility* and *Paranoid ideation*. BPDCL *Emptiness* correlated the strongest with the BSI *Depression* scale (.58). *Anger* showed a very strong correlation with its corresponding BSI subscale *Hostility* (.85). *Paranoid ideation* had the highest correlation with the *Obsession* scale (.68), as well with its corresponding BSI scale *Paranoid* (.64).

The WHO scales *Positive feelings, Negative feelings* and *Social* correlated acceptably with the total BPDCL score. The BPDCL did not correlate acceptably with the WSAS total score.

**eTable 6.**

*Means and standard deviations of other psychological instruments for the Greek sample*

|  | N | Minimum | Maximum | Mean | SD |
| --- | --- | --- | --- | --- | --- |
| BPDSI Abandonment | 32 | 0.43 | 8.71 | 3.60 | 2.01 |
| BPDSI Interpersonal | 32 | 0.38 | 7.63 | 3.41 | 1.77 |
| BPDSI Identity | 32 | 3.13 | 9.38 | 6.73 | 1.66 |
| BPDSI Impulsivity | 32 | 0.00 | 3.82 | 1.73 | 1.02 |
| BPDSI Parasuicidal | 32 | 0.00 | 4.85 | 1.08 | 1.13 |
| BPDSI Affective | 32 | 4.80 | 10.00 | 7.89 | 1.33 |
| BPDSI Emptiness | 32 | 3.00 | 9.75 | 6.71 | 1.82 |
| BPDSI Anger | 32 | 0.17 | 6.83 | 2.71 | 1.87 |
| BPDSI Dissociation | 32 | 0.00 | 5.63 | 2.04 | 1.36 |
| BPDSI Total Score | 32 | 20.86 | 56.73 | 35.91 | 8.71 |
| WSAS total | 32 | 3.00 | 37.00 | 23.84 | 7.76 |
| Somatization scale BSI | 32 | 0.00 | 3.57 | 0.93 | 0.84 |
| Obsessive scale BSI | 32 | 0.50 | 3.50 | 2.11 | 0.68 |
| Insecurity scale BSI | 32 | 0.50 | 3.50 | 2.14 | 0.79 |
| Depressive scale BSI | 32 | 0.67 | 3.83 | 2.64 | 0.80 |
| Anxiety scale BSI | 32 | 0.17 | 3.67 | 1.53 | 0.95 |
| Hostility scale BSI | 32 | 0.00 | 4.00 | 2.01 | 1.18 |
| Phobic anxiety scale BSI | 32 | 0.00 | 4.00 | 0.91 | 0.92 |
| Paranoid scale BSI | 32 | 0.20 | 4.00 | 1.88 | 0.93 |
| Psychoticism scale BSI | 32 | 0.20 | 3.20 | 1.93 | 0.78 |
| Sumscore BSI | 32 | 29.00 | 174.00 | 93.03 | 30.13 |
| WHO Self-esteem | 32 | 1.00 | 3.50 | 2.11 | 0.68 |
| WHO Positive feelings | 32 | 2.25 | 5.00 | 3.91 | 0.59 |
| WHO Negative feelings | 32 | 1.00 | 3.50 | 2.03 | 0.55 |
| WHO Physical | 32 | 14.29 | 57.14 | 35.94 | 11.79 |
| WHO Psychological | 32 | 16.67 | 58.33 | 36.33 | 11.11 |
| WHO Social | 32 | 0.00 | 75.00 | 25.52 | 18.32 |
| WHO Environment | 32 | 18.75 | 75.00 | 49.80 | 14.29 |

**eTable 7.**

*Spearman’s Rho correlations of the Greek BPDCL and the BPDSI*

|  | BPDSI Abandonment | BPDSI  Relation | BPDSI Identity | BPDSI Impulsive | BPDSI  Parasuicid. | BPDSI  Affective | BPDSI  Emptiness | BPDSI Anger | BPDSI  Dissociative | BPDSI  Total |
| --- | --- | --- | --- | --- | --- | --- | --- | --- | --- | --- |
| BPDCL subscales |  |  |  |  |  |  |  |  |  |  |
| Abandonment | **.70**** | .55** | .34 | .49** | .45** | .39* | .35 | .53** | .07 | **.68**** |
| Interpersonal relationships | .46** | **.27** | .30 | .29 | .43* | .29 | .29 | .56** | .10 | **.52**** |
| Identity disturbance | .53** | .46** | **.51**** | .33 | .46** | .39* | .34 | .49** | .06 | **.69**** |
| Impulsivity | .39* | .32 | .38* | **.62**** | .34 | .35 | .33 | .30 | .12 | **.59**** |
| Parasuicidal behavior | .37* | .20 | .54** | .34 | **.81**** | .35* | .72** | .43* | .05 | **.64**** |
| Affective instability | .46** | .37* | .30 | .44* | .60** | **.38*** | .41* | .61** | .27 | **.67**** |
| Emptiness | .06 | .15 | .31 | -.02 | .35* | .29 | **.24** | .31 | .00 | **.35** |
| Anger | .61** | .51** | .23 | .23 | .29 | .47** | .28 | **.77**** | -.01 | **.62**** |
| Paranoid ideation | .26 | .26 | .40* | .25 | .45** | .41* | .40* | .44* | **.46**** | **.54**** |
| Total score | **.57**** | **.51**** | **.54**** | **.45**** | **.57**** | **.47**** | **.45*** | **.59**** | **.19** | **.78**** |

**Note.** ** Correlation is significant at the 0.01 level (two-tailed). * Correlation is significant at the 0.05 level (two-tailed). Correlations without a star are not significant. BPDCL= Borderline Personality Disorder Checklist, BPDSI= Borderline Personality Disorder Severity Index.

**eTable 8.**

*Spearman's Rho correlations of the Greek BPDCL and the BSI-53*

|  | BSI Somatization | BSI Obsession | BSI Insecurity | BSI Depression | BSI  Anxiety | BSI  Hostility | BSI Phobia | BSI Paranoid | BSI Psychotic | BSI Total |
| --- | --- | --- | --- | --- | --- | --- | --- | --- | --- | --- |
| BPDCL subscales |  |  |  |  |  |  |  |  |  |  |
| Abandonment | -.07 | .49** | **.59**** | .49** | .08 | .49** | .17 | .51** | .51** | **.47**** |
| Interpersonal relationships | .00 | .52** | .57** | .38* | .04 | .58** | .19 | .68** | .68** | **.58**** |
| Identity disturbance | -.08 | .43* | .62** | .49** | -.05 | .43* | .20 | .53** | .62** | **.43*** |
| Impulsivity | -.29 | .15 | .22 | .30 | -.27 | .16 | .18 | .35 | .43* | **.07** |
| Parasuicidal behavior | .18 | .65** | .44* | .60** | .15 | .29 | .15 | .27 | .63** | **.55**** |
| Affective instability | .21 | .67** | .58** | .58** | .34 | .59** | .14 | .60** | .66** | **.76**** |
| Emptiness | -.30 | .24 | .44* | .58** | -.17 | .24 | -.14 | .40* | .44* | **.25** |
| Anger | .19 | .41* | .36* | .26 | .41* | **.85**** | .12 | .55** | .46** | **.61**** |
| Paranoid ideation | .25 | .68** | .38* | .27 | .23 | .41* | .17 | **.64**** | **.61**** | **.64**** |
| Total score | **.02** | **.57**** | **.56**** | **.51**** | **.10** | **.58**** | **.18** | **.64**** | **.69**** | **.59**** |

*Note.* ** Correlation is significant at the 0.01 level (two-tailed). * Correlation is significant at the 0.05 level (two-tailed). Correlations without a star are not significant. BSI= Brief Symptom Inventory-53.

**eTable 9.**

*Spearman’s Rho correlations of the Greek BPDCL and WHO subscales*

|  | WHO  Self-esteem | WHO  Negative | WHO  Positive | WHO  Physical | WHO  Psychological | WHO  Social | WHO  Environment |
| --- | --- | --- | --- | --- | --- | --- | --- |
| BPDCL subscales |  |  |  |  |  |  |  |
| Abandonment | -.38* | .53** | -.21 | -.47** | -.29 | -.46** | -.11 |
| Interpersonal relationships | -.30 | .36* | -.26 | -.38* | -.21 | -.55** | -.29 |
| Identity disturbance | -.34 | .33 | -.51** | -.47** | -.36* | -.53** | -.01 |
| Impulsivity | -.38* | .17 | -.29 | -.21 | -.48** | -.45* | -.21 |
| Parasuicidal behavior | -.22 | .40* | -.63** | -.26 | -.42* | -.38* | -.38* |
| Affective instability | -.32 | .62** | -.32 | -.39* | -.22 | -.50** | -.16 |
| Emptiness | -.19 | .35 | -.36* | -.57** | -.41* | -.46** | -.02 |
| Anger | -.15 | .39* | -.41* | -.33 | -.24 | -.42* | -.04 |
| Paranoid ideation | -.09 | .40* | -.26 | -.14 | -.14 | -.33 | -.32 |
| Total score | **-.32** | **.47**** | **-.46**** | **-.38*** | **-.38*** | **-.54**** | **-.12** |

*Note.* ** Correlation is significant at the 0.01 level (two-tailed). * Correlation is significant at the 0.05 level (two-tailed). Correlations without a star are not significant. BPDCL= Borderline Personality Disorder Checklist, WHO= World Health Organization Quality of Life.

**eTable 10.**

*Spearman’s Rho correlations of the Greek BPDCL and WSAS Total Score*

|  | WSAS  Total score |
| --- | --- |
| BPDCL subscales |  |
| Abandonment | .22 |
| Interpersonal relationships | .16 |
| Identity disturbance | .40* |
| Impulsivity | .36* |
| Parasuicidal behavior | .49* |
| Affective instability | .16 |
| Emptiness | .16 |
| Anger | .17 |
| Paranoid ideation | .10 |
| Total score | **.35** |

*Note.* ** Correlation is significant at the 0.01 level (two-tailed).

* Correlation is significant at the 0.05 level (two-tailed).

Correlations without a star are not significant.

BPDCL= Borderline Personality Disorder Checklist,

WSAS= Work and Social Adjustment Scale.

### ***Conclusion***

The Greek sample consisted of 32 BPD patients. The Cronbach’s Alpha was .95. The reliability coefficients of the subscales ranged from .63(*Parasuicidal behavior*) to .87 (*Anger*). Validity coefficients were estimated. However. the results should be looked at cautiously, given the small sample size (n=32). Further investigation on the psychometric qualities (e.g. known-groups validity) of the Greek BPDCL is needed.
